# On the Question of the Regio-Orientation, Stereo-Orientation and Molecular Mechanism in the Cascade Cycloaddition/Rearrangement/Elimination Processes Leading to Nitro-Substituted Thiopyran Analogs: DFT Computational Study

**DOI:** 10.3390/ijms26188948

**Published:** 2025-09-14

**Authors:** Mikołaj Sadowski, Ewa Dresler, Radomir Jasiński

**Affiliations:** 1 Cracow University of Technology, CUT Doctoral School, Faculty of Chemical Engineering and Technology, Warszawska 24, 31-155 Cracow, Poland; mikolaj.sadowski@doktorant.pk.edu.pl; 2 Łukasiewicz Research Network—Institute of Heavy Organic Synthesis “Blachownia”, Energetyków 9, 47-225 Kędzierzyn-Koźle, Poland; ewa.dresler@icso.lukasiewicz.gov.pl; 3 Cracow University of Technology, Department of Organic Chemistry and Technology, Warszawska 24, 31-155 Cracow, Poland

**Keywords:** cycloaddition, hetero Diels–Alder reaction, nitroalkene, stepwise mechanism, deamination, density functional theory, Lewis acid (LA) catalysis

## Abstract

Sulfur-containing heterocyclic structures play an important role in modern biotechnology. Their synthesis is made possible by means of the hetero Diels–Alder reaction involving unsaturated sulfur compounds. In the framework of this paper, the molecular mechanism of the cycloaddition reactions between tioanalogs of the butadiene generated in situ with the participation of the *Lawesson* reagent and the E-2-phenyl-1-nitroethene was evaluated on the basis of the DFT quantum chemical calculations. It was found that the most favored reaction path is realized according to a stepwise mechanism with the participation of the zwitterionic intermediate. To study this further, the molecular mechanism of the deamination process of the primary cycloadducts was also analyzed. It was found that this mechanism is substantially different to the case of other known β-elimination processes and is achieved via a stepwise scheme. In addition to these investigations, the LA catalysis of the deamination process was also explored.

## 1. Introduction

**Scheme 1:**
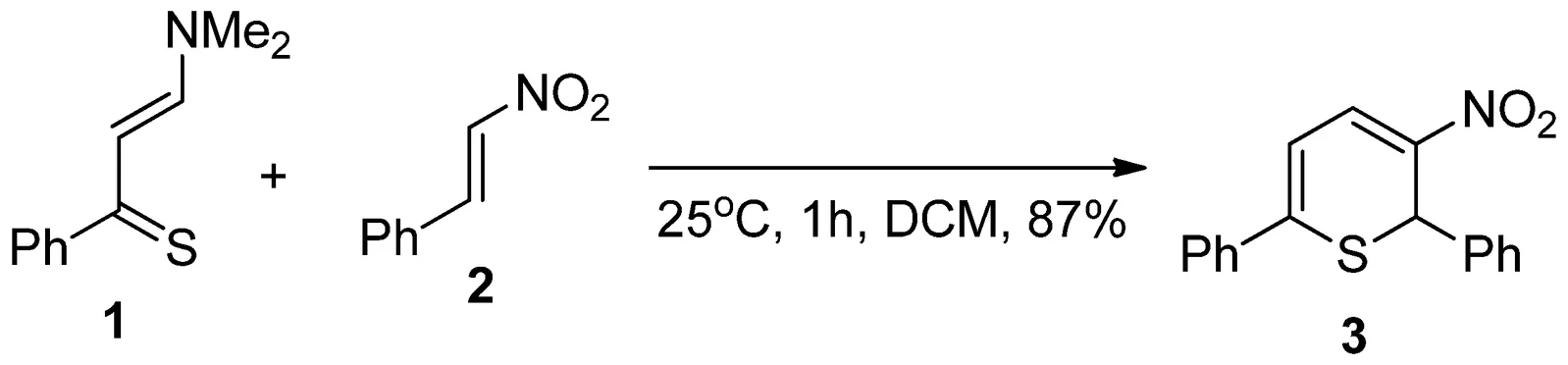
Experimentally observed results of the title reaction of 2-phenylnitroethene **2** in the DCM (dichloromethane) solution.

Sulfur-containing heterocyclic structures play an important role in modern biotechnology, pharmacy, and related areas because of their different bioactivities such as anticancer [1], antiviral [1,2], antifungal [3,4,5], insecticidal [1,6], and herbicidal activity [1,7]. Some compounds from this group can be obtained from natural matrices [8,9]. However, a more popular way to obtain sulfur-containing heterocycles is organic synthesis. In particular, the most universal approach is based on the application of (2 + 2)- [10,11], (3 + 2) [12,13], and (4 + 2) cycloaddition processes [12,14]. The hetero Diels–Alder (HDA) reaction can be used for the preparation of thiopyrane analogs starting from conjugated dienes and tioketones or tioanalogs of the buta-1,3-diene and alkenes as 2-π-components. These processes should be treated formally as (4 + 2) cycloadditions. In contrast to the “carbo” Diels–Alder (DA) reactions [15,16], in most cases, the hetero Diels–Alder processes require the presence of Lewis acid (LA) catalysts [17,18]. Only some incidental cycloaddition reactions of this type occur under thermal conditions [19,20]. Recently, *Karpov* and coworkers [21] described a protocol for the preparation of new nitrofunctionalized analogs of tiopyrane on the basis of the reaction between tioanalogs of butadiene generated in situ with the participation of the *Lawesson* reagent (Scheme 1). The key stage of this process, used for the construction of the heterocyclic segment, is probably the (4 + 2) cycloaddition reaction.

**Scheme 2:**
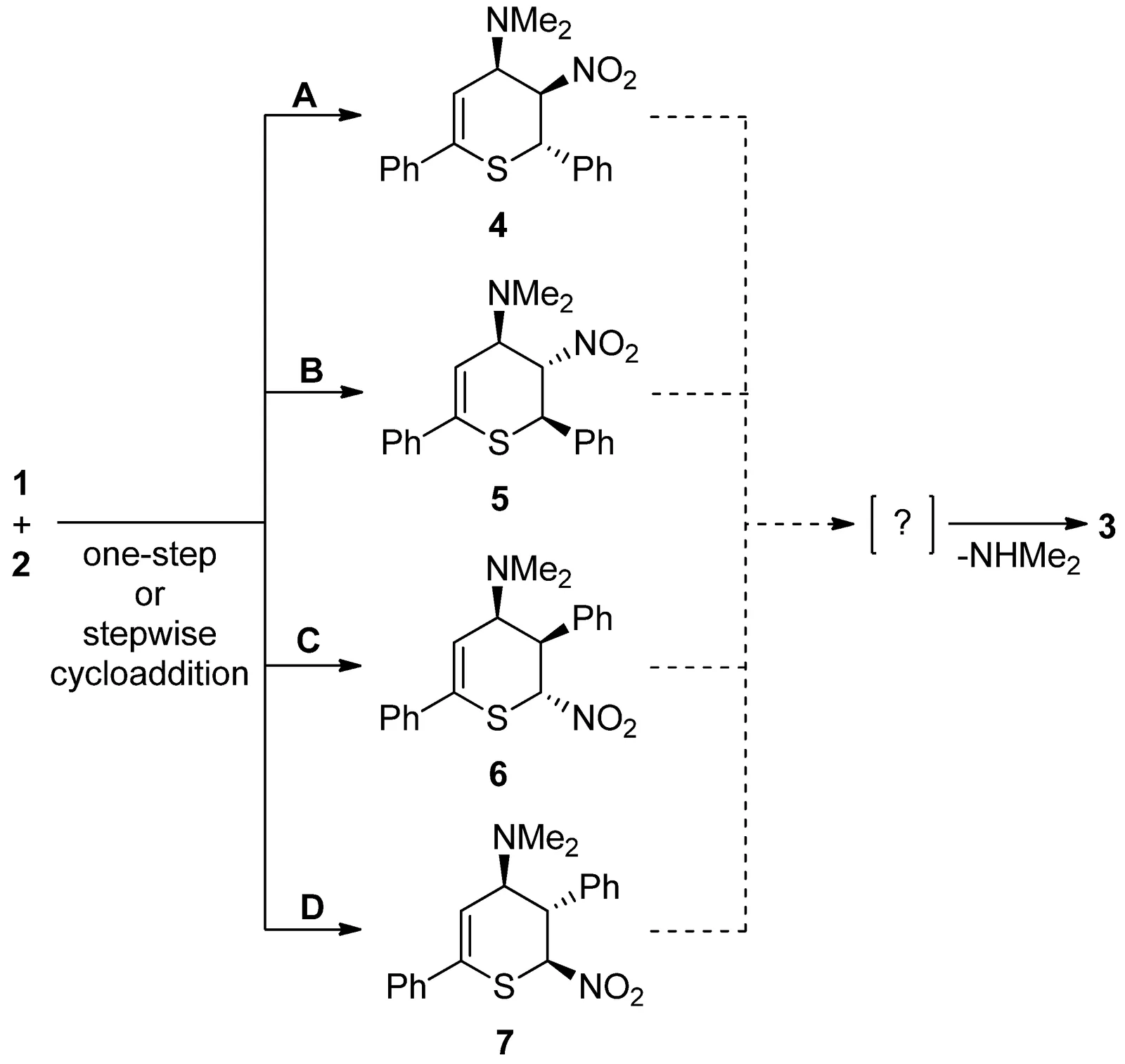
Theoretically possible, regio- and stereoisomeric HDA **1** + **2** cycloaddition paths.

The reaction course and mechanism are probably difficult and include an undetermined addition/elimination sequence (Scheme 2). Thus, it is not possible to explain it without additional, comprehensive mechanistic studies. In particular, several important problems were detected:

- The question of the regioselectivity of the cycloaddition reaction (Scheme 1). The regio-orientation of the detected final products suggests that the 1,5-diphenyl-3-dimethylamino-4-nitrohexane-6-tio-cyclohexa-1-ene (**3** or **4**) should be considered the primary cycloaddition product. It is, however, possible that this product is formed as a result of the cycloreversion of less thermodynamically stable 1,4-diphenyl-3-dimethylamino-5-nitrohexane-6-tio-cyclohexa-1-ene (**5** or **6**) that forms more easily from the kinetic point of view. This type of balance between thermodynamic and kinetic factors regarding the problem of cycloaddition regioselectivity was observed in the case of reactions with participation of conjugated nitroalkenes [22,23,24,25].
- The problem of the stereoselectivity of the cycloaddition reaction (Scheme 1). In the framework of both regioisomeric approaches, two stereoisomeric reaction channels are possible due to the tendency to form 3,4-cis- and 3,4-trans cycloadducts. Assuming the syn-mechanism of the deamination stage, the last one should be more probable. However, this mechanism was not analyzed in any way. In the case of the stepwise elimination mechanism, the final product can be formed from both stereoisomeric 1,5-diphenyl-3-dimethylamino-4-nitrohexane-6-tio-cyclohexa-1-enes **4** and **5**. Recently, different types of stepwise mechanisms were documented for elimination reactions earlier defined as a single-step [26,27,28].
- Mechanistic aspect of the cycloaddition process. Three decades ago, the single-step mechanism with the pericyclic reorganization of the electron density was widely accepted [29]. However, recent discoveries undermine this point of view [30]. At this moment it is known that in many cases, the single-step mechanism is not pericyclic [31,32,33]. Moreover, the single-step scheme can compete with stepwise mechanism with biradical [34] or zwitterionic intermediates [35,36]. These issues are not clear regarding this title process.
- The deamination reaction is generally considered a difficult process in comparison to other 1,2-elimination reactions. The theoretically possible, competitive channel of the extrusion of the nitrous acid cannot be, however, achieved realistically. These types of extrusion are generally realized as single-step, pseudocyclic processes [37,38] under mild reaction conditions.
- The deamination process of 2-amino-1-nitroethyl molecular segments is generally significantly accelerated by the presence of the LA-catalysts such as boron hydride or boron trifluoride [39,40,41]. Therefore, in the last part of our research we decided to shed light on the kinetic aspects and the molecular mechanism of the transfer of respective LA to the cycloaddition product and the further decomposition via elimination stage.

Due to issues explained below, the mechanistic aspects of the reactions between tioanalogs of buta-1,3-diene and conjugated nitroalkenes require a comprehensive exploration and interpretation. We decided to resolve these problems on the basis of the density functional theory (DFT) calculations and the analysis in the framework of the molecular electron density theory (MEDT) [42]. These studies were performed for the model process with the participation of 2-phenyl-4-dimethylamino-1-tio-buta-1,3-diene (**1**) and E-2-phenylnitroethene (**2**) (Scheme 1 and Scheme 2).

## 2. Results and Discussion

In the first part of our study, we decided to carry out a detailed analysis of electronic properties of components for model reactions in framework of the conceptual functional density theory (CDFT) [43,44]. On this basis, the discussion about the global and local aspects of organic reactivity was performed. Recently, a similar approach has been successfully applied to the interpretation of a wide range of bimolecular reactions [45,46,47,48].

Electronic properties of E-2-phenylnitroethene **2** were discussed in detail very recently [49]. It was found that the nitroalkene **2** should be treated as a strong electrophile (ω = 2.67 eV), characterized by electrophilically activated 2 position of the nitrovinyl molecular segment. On the other hand, the global electrophilicity of the second addition component is substantially lower (ω = 1.00 eV). Therefore, in the light of Domingo terminology [50], the difference between electrophilicities requires treating analyzed processes as clearly polar, determined by the forward electron density flux (FEDF) [51]. Subsequently, it was found that molecule **1** is characterized by a great value of the global nucleophilicity index (N = 1.76 eV). The difference between local nucleophilicities within molecule **1** is, however, not significant. Therefore, the local reactivity of this reaction component will be a result of the balance between local electronic factors and steric repulsions. In conclusion, in this case, the CDFT approach does not offer the possibility of predicting the reaction’s regioselectivity.

**Figure 1:**
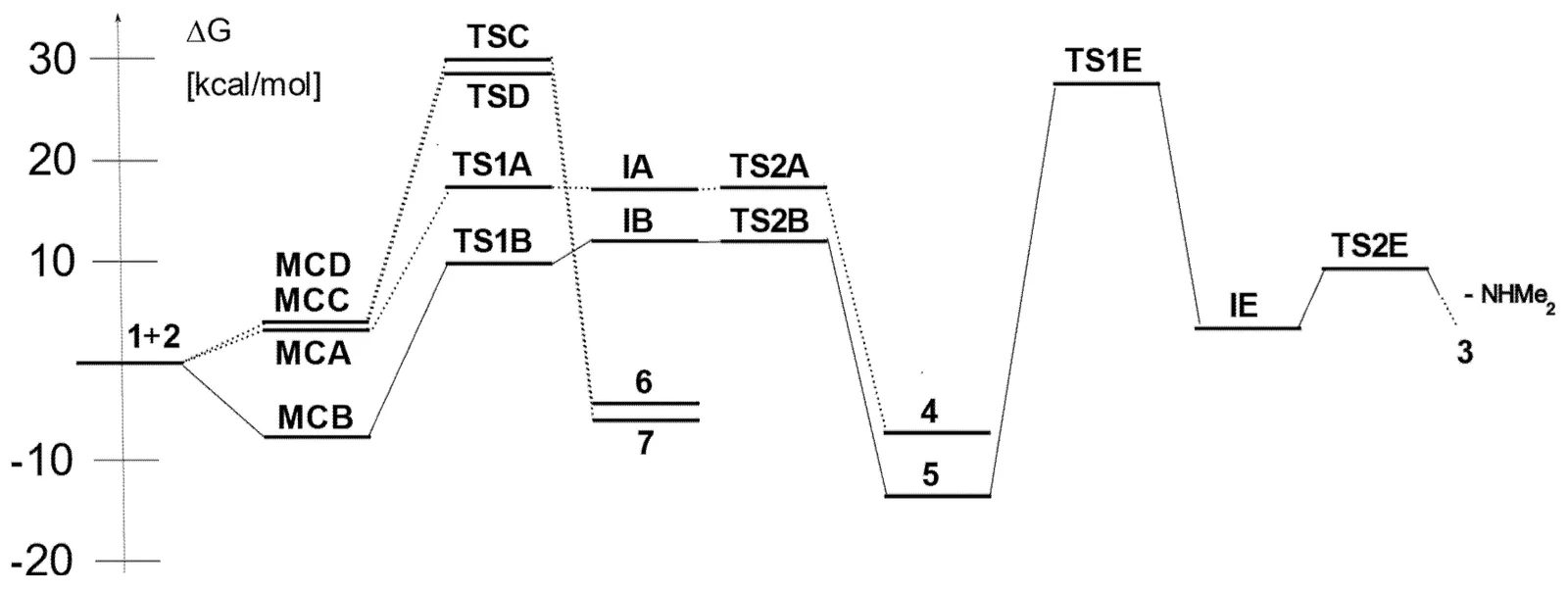
Enthalpy profile for the **1** + **2** HDA reaction in the DCM solution and the further deamination process according to ωB97XD/6-311G(d) (PCM) quantum chemical calculations.

**Figure 2:**
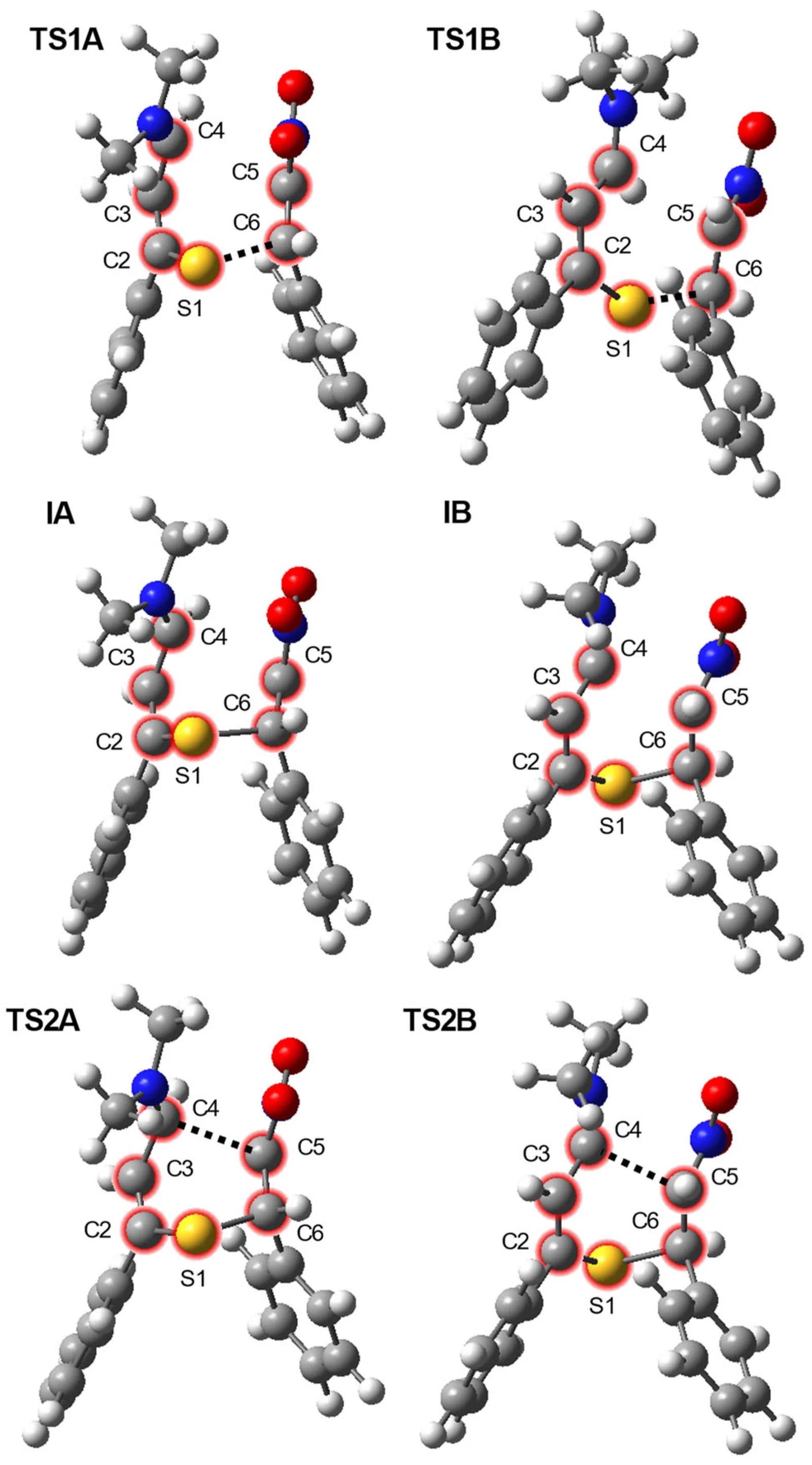
Views of critical structures of paths **A** and **B** of the **1** + **2** HDA reaction in the DCM solution according to ωB97XD/6-311G(d) (PCM) quantum chemical calculations.

**Figure 3:**
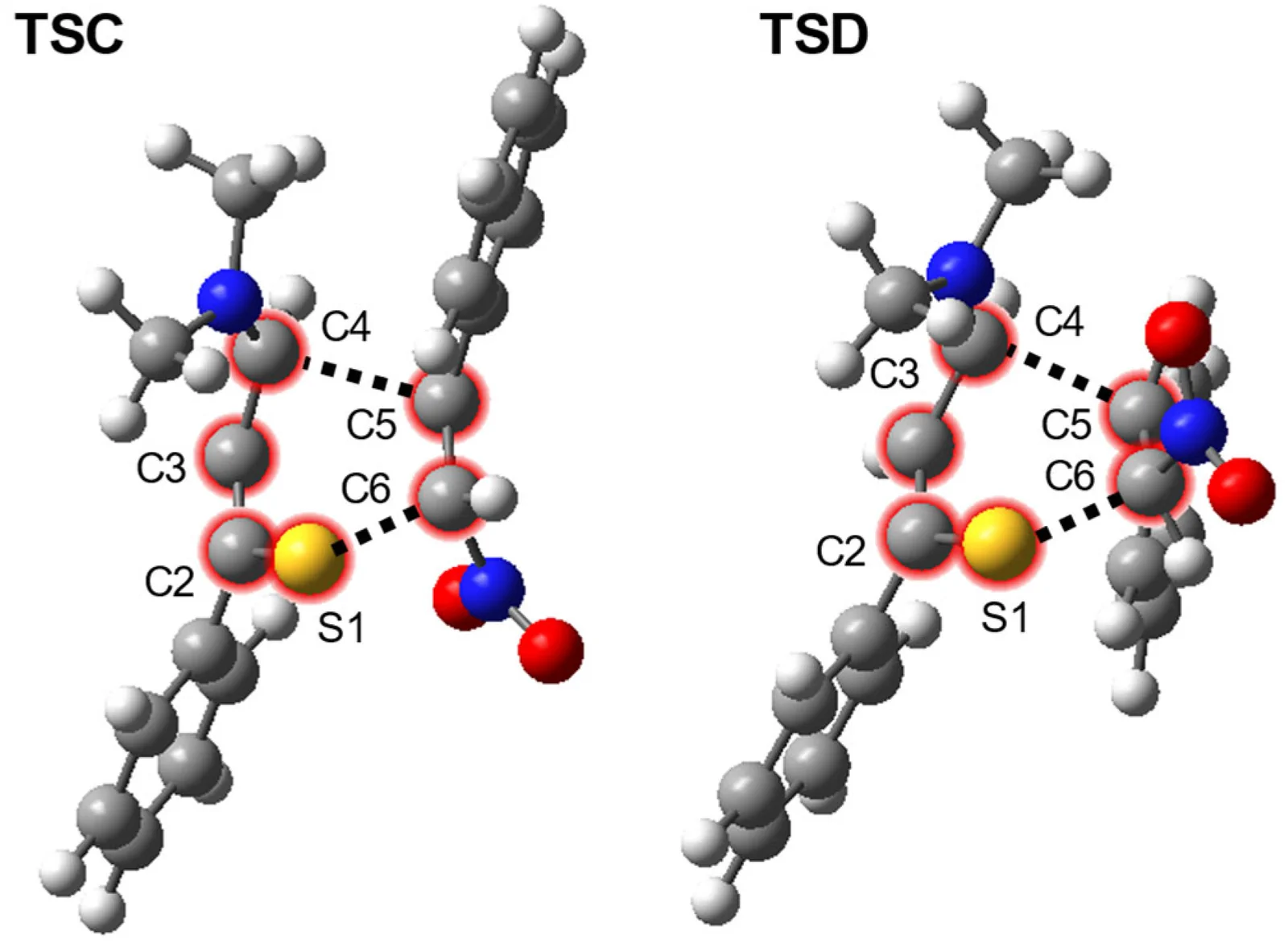
Views of critical structures of paths **C** and **D** of the **1** + **2** HDA reaction in the DCM solution according to ωB97XD/6-311G(d) (PCM) quantum chemical calculations.

Next, the reaction profiles for all possible cycloaddition paths (Scheme 2) were analyzed, based on the results of the ωB97XD/6-311G(d) calculations. The presence of the DCM in the reaction was simulated via the polarizable continuum model (PCM) using the integral equation formalism variant (IEFPCM) as the default SCRF (self-consistent reaction field) method (see the Computational Details section). It was found that the nature of reaction profiles is completely different for reactions leading to regioisomeric 1,5-diphenyl-3-dimethylamino-4-nitrohexane-6-tio-cyclohexa-1-enes **4**/**5** (paths **A** and **B**) and 1,4-diphenyl-3-dimethylamino-5-nitrohexane-6-tio-cyclohexa-1-enes **6/7** (paths **C** and **D**) (Figure 1, Figure 2 and Figure 3). 

Within paths **A** and **B**, the intermolecular interactions lead initially to the formation of pre-reaction complexes (**MCA** and **MCB**, respectively). This transformation is realized as barrierless and is associated with the reduction in the enthalpy of the reaction system. For path **A**, this change is equal to 10 kcal/mol, whereas for path **B**, analogous change is greater and equals more than 20 kcal/mol (Table 1). The influence of entropic factors on the relative stability of mentioned intermediates is very important. This factor is approximately 40 cal/molK for both considered transformations. In the case of path A, this fact determines the positive value of the Gibbs free energy of the formation of **MCA**. This excludes the possibility of existence of the **MCA** as a stable intermediate. In the case of path **B**, the Gibbs free energy of the formation of **MCB** is still negative, which stimulates the potential stability of the pre-reaction complex as a reaction intermediate. This fact favors the way of the title reaction via path **B** to a larger extent than via path **A**. Independently of different energetic characteristic, both pre-reaction complexes exhibit similar structural character. In particular, interatomic distances S1–C2, C2–C3, C3–C4, and C5–C6 (Figure 2) distances derived from addend molecules have not changed in comparison to geometry within individual **1** and **2** structures (Table 2). In the framework of MCs, **1** and **2** substructures adopt orientation, which determines further conversion to respective transition state. Therefore, **MCA** and **MCB** should be treated as orientation complexes. It should be, however, underlined that at this stage, key distances between reaction centers (Table 2) do not adapt values typical for new sigma bonds in transition states [52,53,54,55]. Lastly, within MCs, the electron density transfer between substructures is not achieved (GEDT = 0.00 e). Therefore, the optimized structures should not be considered charge transfer complexes. Similar pre-reaction intermediates were very recently detected regarding to different type cycloaddition processes [56,57,58].

The further conversion of pre-reaction intermediates **MCA** and **MCB** leads directly to respective transition state (**TS1A** and **TS1B**, respectively). Within these transition states, only one (C6–S1 bond) of the two sigma bonds necessary for the completion of target heterocyclic ring is formed. The formation of **TS1A** and **TS1B** is associated with the great transfer of the electron density (see GEDT (global electron density transfer) values in Table 2). This confirms the polar nature of explored process. The IRC (intrinsic reaction coordinate) calculations connect mentioned TSs with the transition valley of the respective reaction intermediate (**IA** and **IB** for paths **A** and **B**, respectively). The nature of these intermediates was explored on the basis of detailed ELFs (electron localization functions) experiments. We performed this study for the **IB** structure.

**Figure 4:**
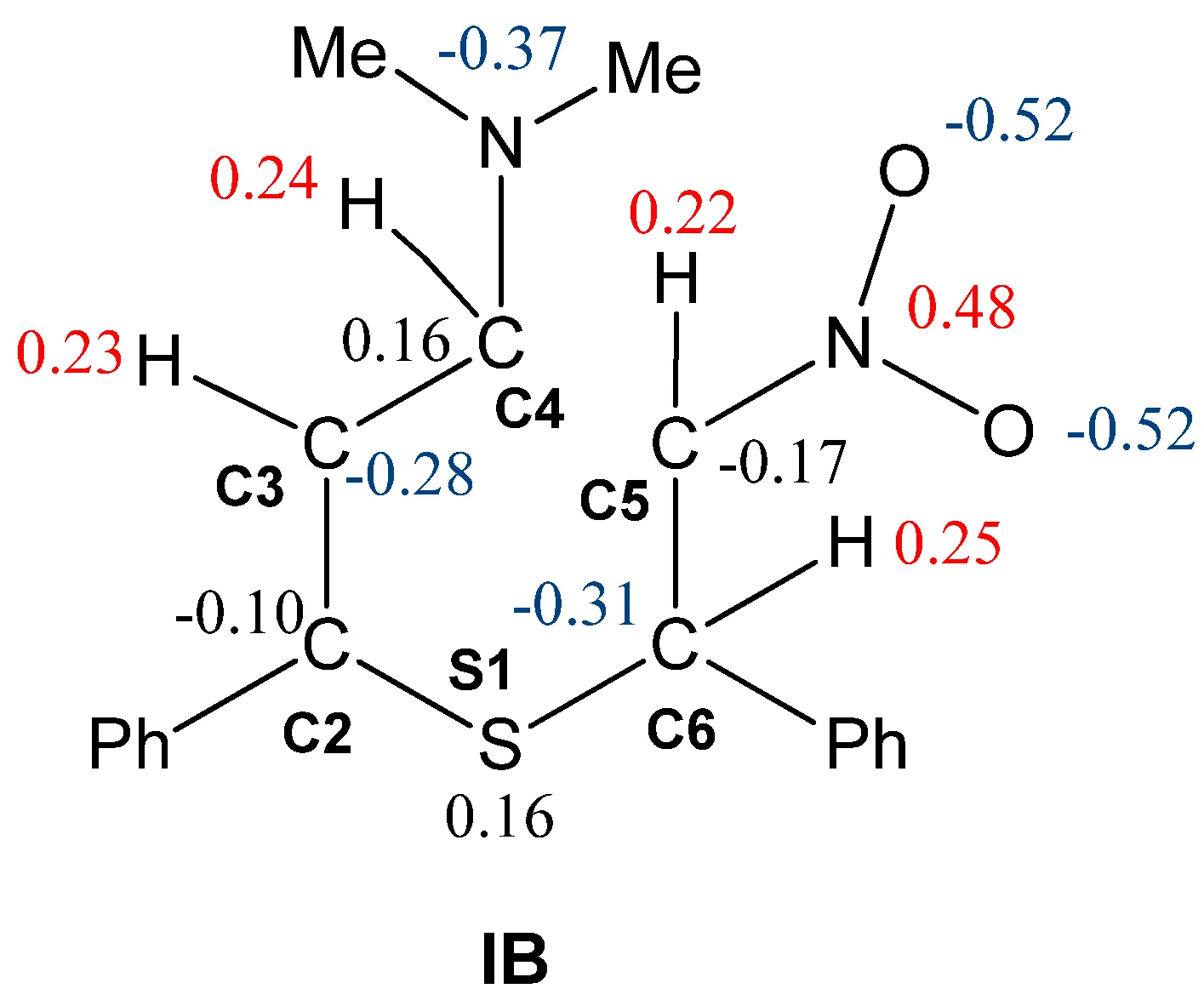
Natural charges of significant atoms (in electrons) in the **IB** as computed at the ground state via ωB97X-D/6-311G(d,p) (PCM) quantum chemical calculations; charges > 0.2 e are given in red, charges < −0.2 e are given in blue. For clarity: bond order and bond lengths were neglected; insignificant parts are shown in gray and abbreviated.

In the NPA (natural population analysis (Figure 4), in the reaction site, only one carbon atom (C4) has a slightly positive charge (+ 0.16); the atom corresponding to C4 in the assumed reaction course (C5) has a charge of −0.17. All non-hydrogen atoms neighboring the C4 atom have negative charges.

**Figure 5:**
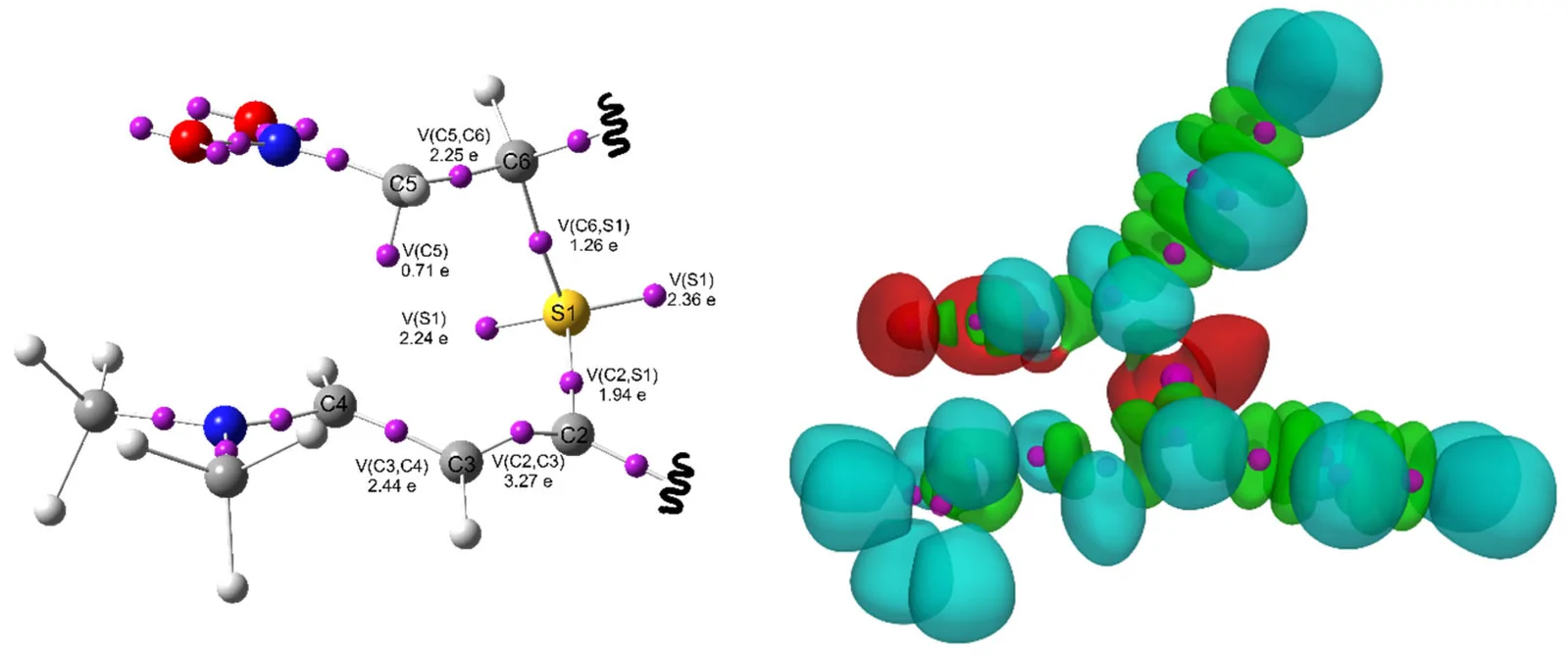
ELF results for **IB** as computed via ωB97X-D/6-311G(d,p) (PCM) calculations. To the right: topology of the ELF function, isovalue 0.75. Monosynaptic valence basins are given in red, disynaptic in green, protonated basins in cyan, and core basins in magenta. To the left: positions of ELF attractors (in magenta) and populations of significant basins. For clarity, some parts of the system are omitted.

In the ELF analysis, a monosynaptic valence basin V(C5) with population of 0.71 e is present. The basin’s attractor is located on a line connecting C4 and C5 atoms (Figure 5).

**Figure 6:**
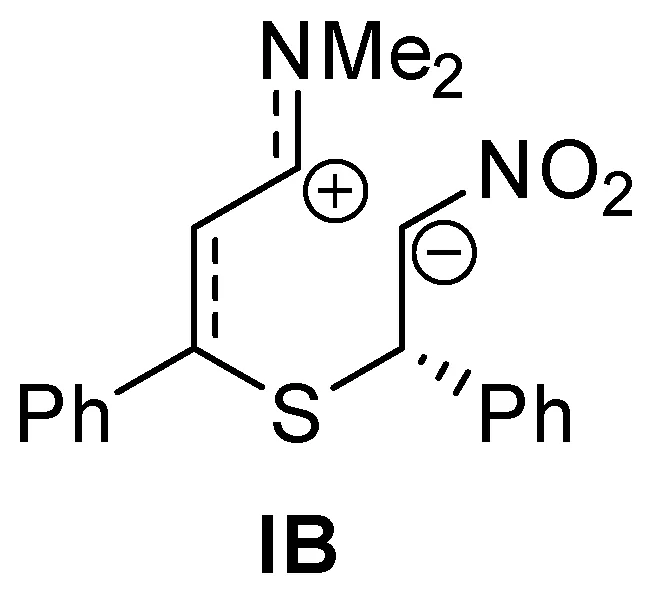
Lewis-like structure of **IB**, for clarity insignificant parts are abbreviated.

The monosynaptic V(C5) basin when interpreted with the C5 atom natural charge allows us to assume that the intermediate **IB** has excessive electron density located at the C5 carbon atom. This paired with the positive charge at C4 carbon atom shows that the intermediate **IB** exhibits zwitterionic nature (Figure 6).

The cyclization of **IA** and **IB** intermediates is carried out as a single-step process via **TS2A** and **TS2B** transition states, respectively. This only requires very little energy and is connected with the formation of the C4–C5 new sigma bond. Both considered TSs exhibit a polar nature. The IRC calculations confirm that **TS2A** and **TS2B** are directly connected with respective cycloadducts (**4** and **5**, respectively).

The initial phase of reaction according to paths **C** and **D** is analogous to the case of paths **A** and **B**. In this phase, pre-reaction complexes (**MCC** and **MCD**, respectively) are formed. The nature of localized intermediates is generally similar to **MCA** and **MCB**. The scenario of their further conversion is, however, different. In both cases, the transition of the reaction system along the reaction coordinate is realized via single transition state (**TSC** and **TSD**, respectively, for paths **C** and **D**). Within these TSs, two new sigma bonds are formed (C4–C5 and C6–S1). The asynchronicity of the formation of new bonds within localized structures is not high (Table 2). Independently of this fact, **TSC** and **TSD** exhibit great polar nature. This is evident in the light of the GEDT values. The nature of both TSs was confirmed by the IRC calculations. IRC experiments connect localized transition states directly with valleys of respective cycloadducts.

**Scheme 3:**
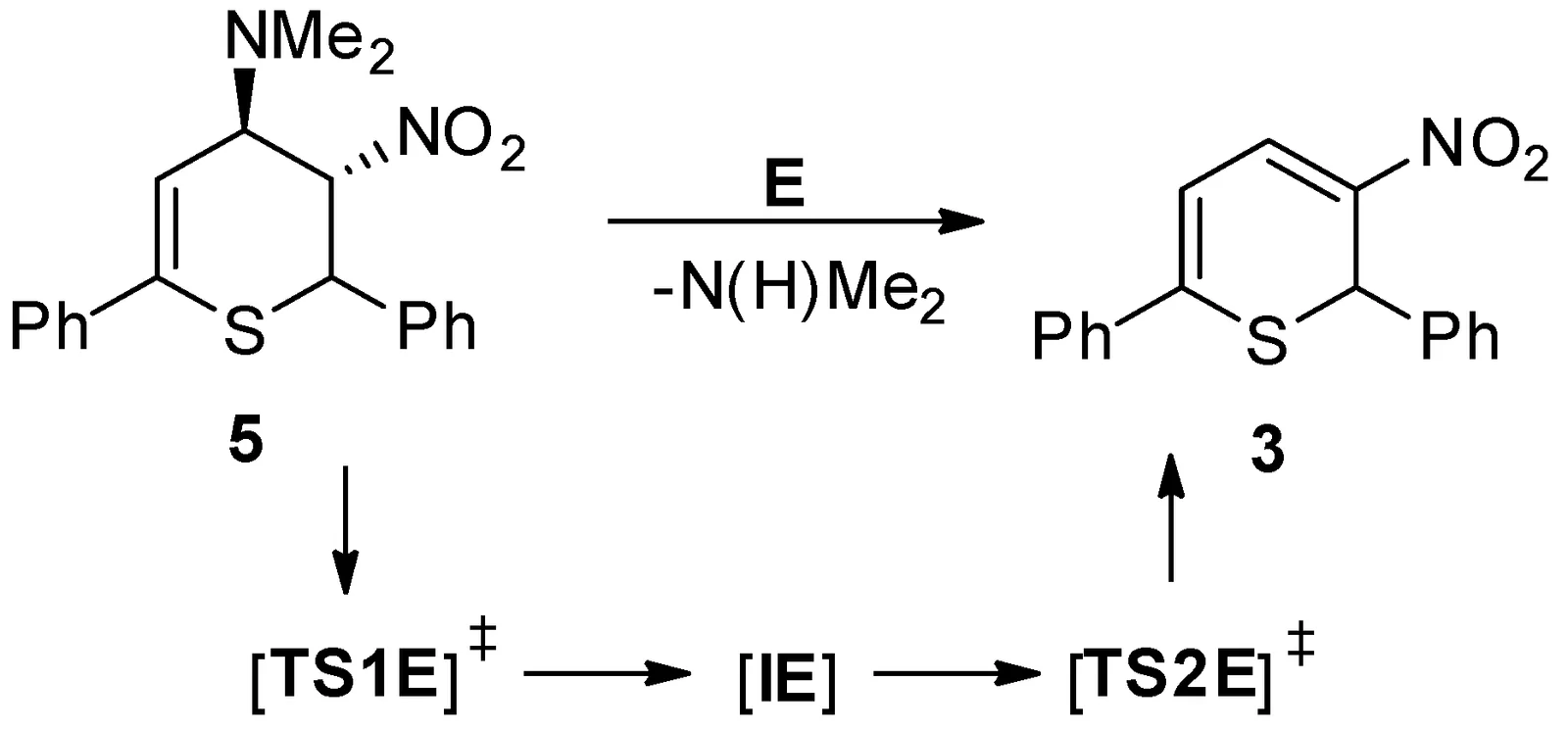
The scheme of the thermal deamination of the adduct **3**.

In general, only one isomeric channel of conversion of the starting molecular system is possible from the kinetic point of view. This is the reaction via path **B**. Competitive reaction ways (**A**, **C**, and **D**) should be treated as forbidden. Therefore, the adduct **5** can only be treated as a starting material for the transformation into target molecule **3** (Scheme 3). This fact excellently correlates with the selectivity observed experimentally (Scheme 1). Next, the reaction according to path **B** is achieved via a very low activation barrier. In consequence, these transformations can occur at room temperature, which perfectly correlates with experimental facts [21]. We found that in contrast to other known thermal β-elimination reactions [37,38,59], the elimination of the dimethylamine from molecule **5** is not realized as a single-step process with the participation of the pseudocyclic transition state.

**Figure 7:**
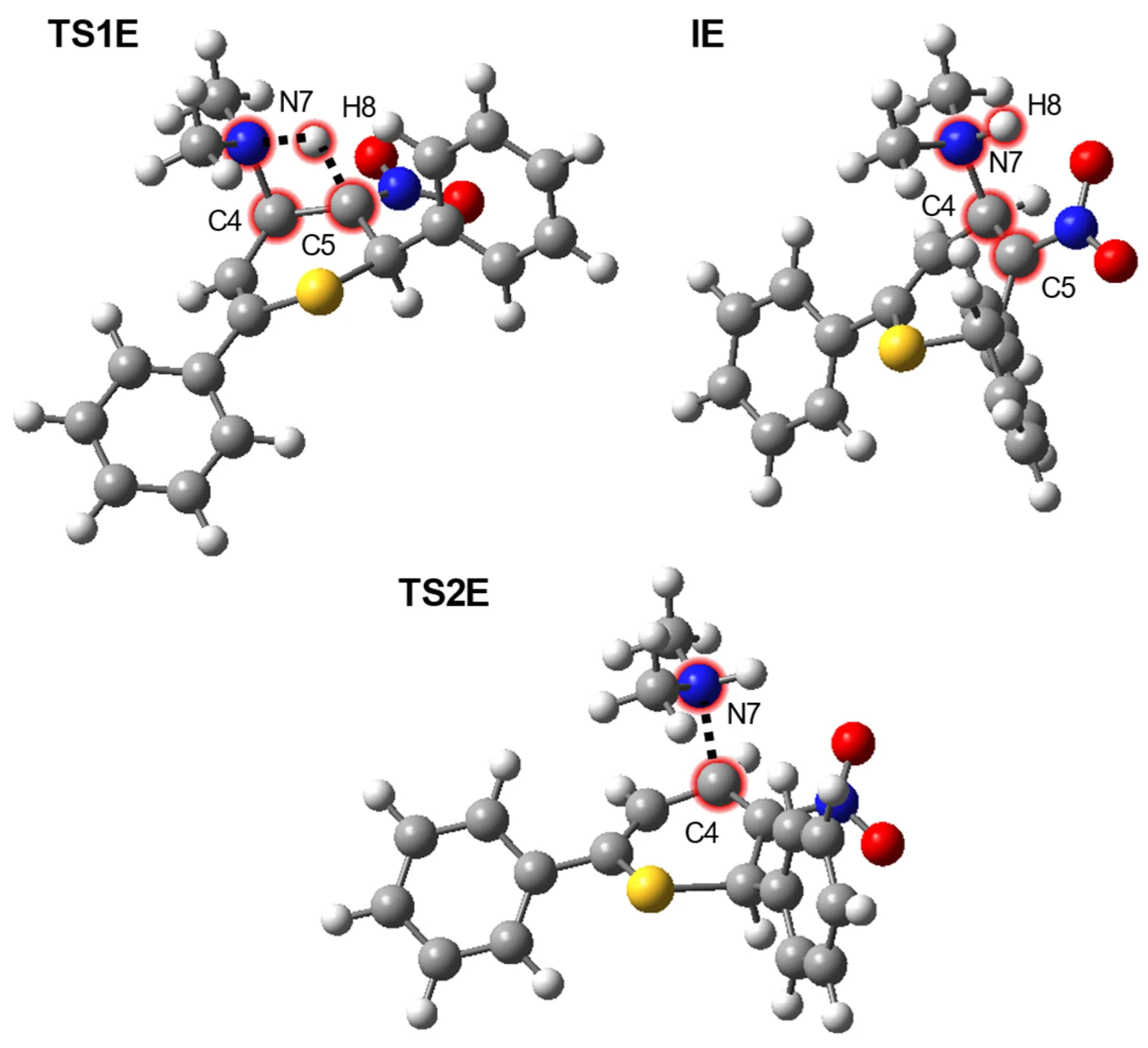
Views of critical structures of reaction paths **E** illustrated on the Scheme 3 (deamination process) in the DCM solution according to ωB97XD/6-311G(d) (PCM) quantum chemical calculations.

The intermediate **IE** (Scheme 3, Figure 7) is unstable from the thermodynamic point of view and easily converts into target molecule **3**. This process is carried out via the **TS2E** transition state and is associated with the dissociation of the single bond between the N7 nitrogen atom from the ammonium group and the C4 carbon atom of the heterocyclic ring (Figure 7).

Within the first reaction stage, the hydrogen atom from the C6 carbon is transferred to the nitrogen atom of the dimethylamine group. This process is realized via the single transition state. This is the **TS1E** structure (Table 3). Within this stage, the C5–H8 single bond dissociates. At the same time, the new N7–H8 single bond is formed. The geometry of the optimized transition state exhibits the nature typical for proton [1.3]-sigmatropic rearrangement [60,61,62].

The obtained transitional state was characterized by ELF and NPA analyses.

**Figure 8:**
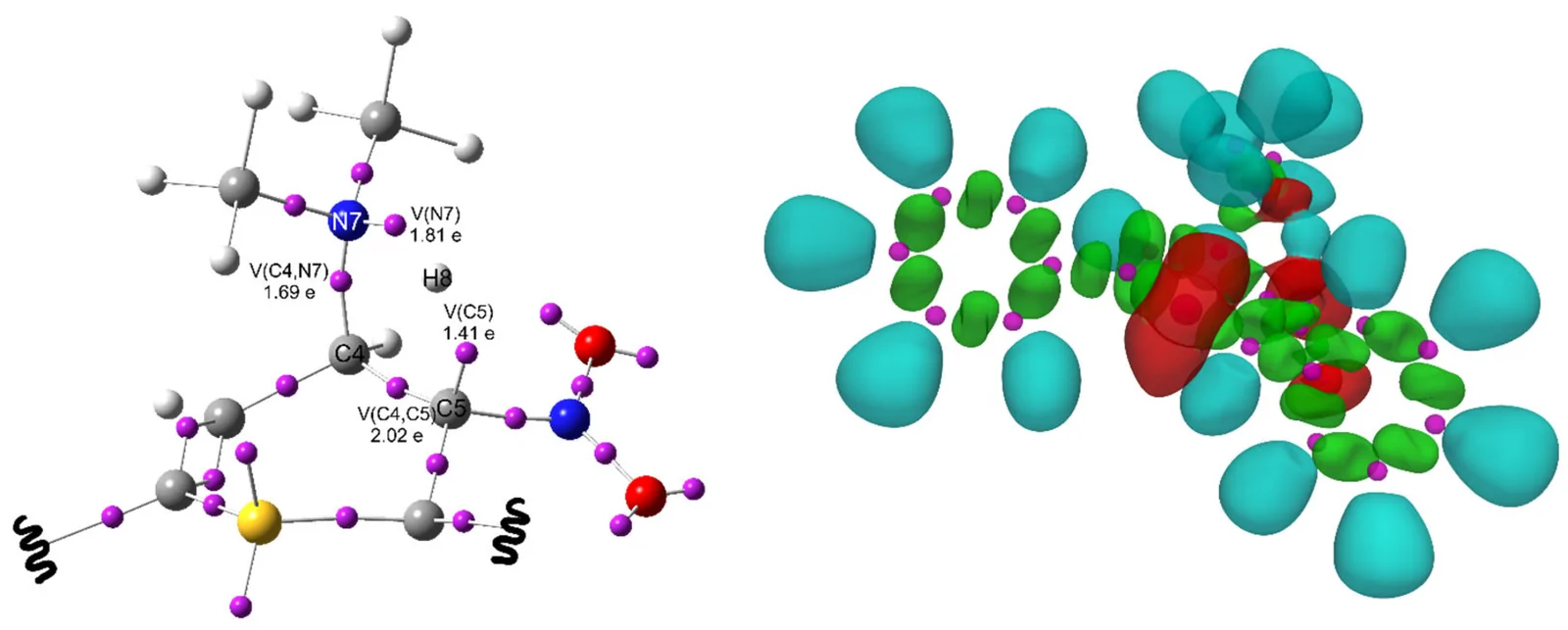
ELF results for **TS1E** as computed via ωB97X-D/6-311G(d,p) (PCM) calculations. To the right: topology of the ELF function, isovalue 0.75. Monosynaptic valence basins are given in red, disynaptic in green, protonated basins in cyan, and core basins in magenta. To the left: positions of ELF attractors (in magenta) and populations of significant basins. For clarity, some parts of the system are omitted.

The **TS1E** ELF analysis (Figure 8) shows monosynaptic basins at the C5 and N7 atoms V(C5) and V(N7) integrating, respectively, 1.41 e and 1.81 e. The attractors present at the disynaptic basin V(C4,N7) and V(C4,C5) are shifted from the regular position observed for attractors of irreducible disynaptic basins. Normally, the attractor lies on a line connecting the centers of the synapses the basin lies at. Here, positions of the attractors suggest that the bonds represented by the basins V(C4,N7) and V(C4,C5) are slightly strained, with populations of 1.69 e and 2.02 e, respectively.

**Figure 9:**
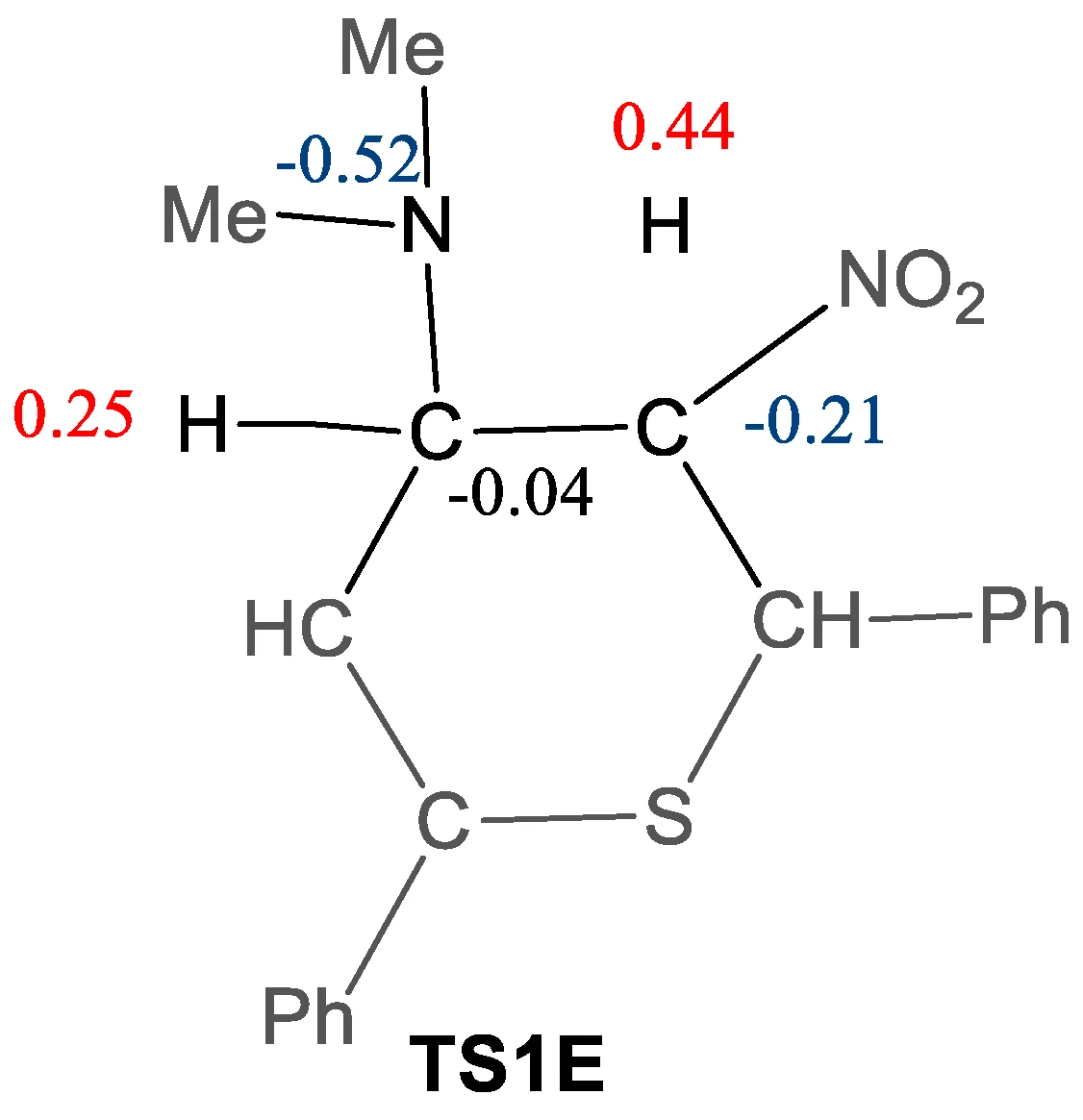
Natural charges of significant atoms (in electrons) in the **TS1E** as computed at the ground state via ωB97X-D/6-311G(d,p) (PCM) quantum chemical calculations; charges > 0.2 e are given in red, charges < −0.2 e are given in blue. For clarity, bond order and bond lengths were neglected; insignificant parts are shown in gray and abbreviated.

According to the NPA results (Figure 9), the C4 and N7 atoms have assigned charges of −0.21 and −0.52, respectively. The H8 atom has a positive charge of 0.44.

**Figure 10:**
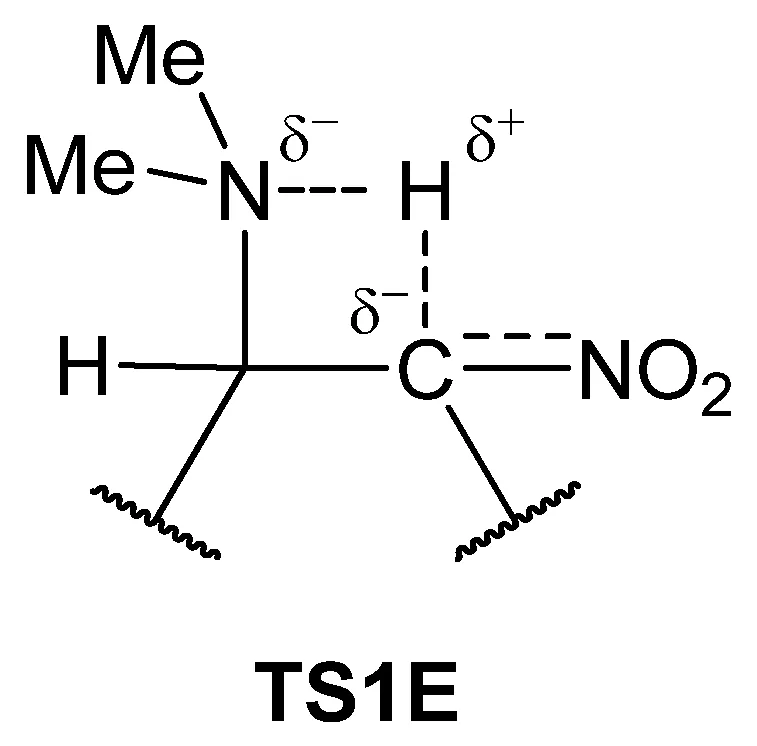
Lewis-like structure of **TS1E**. For clarity, insignificant parts are omitted.

The **TS1E** seems to be of polar nature and to be geometrically strained, as seen in Figure 7, by the bond–bond repulsion, seen as a shift of attractors from their normal positions and by the “buckling” in the topology of V(C4,N7) and V(C4,C5). The structure of the **TS1E** is shown in Figure 10.

**Figure 11:**
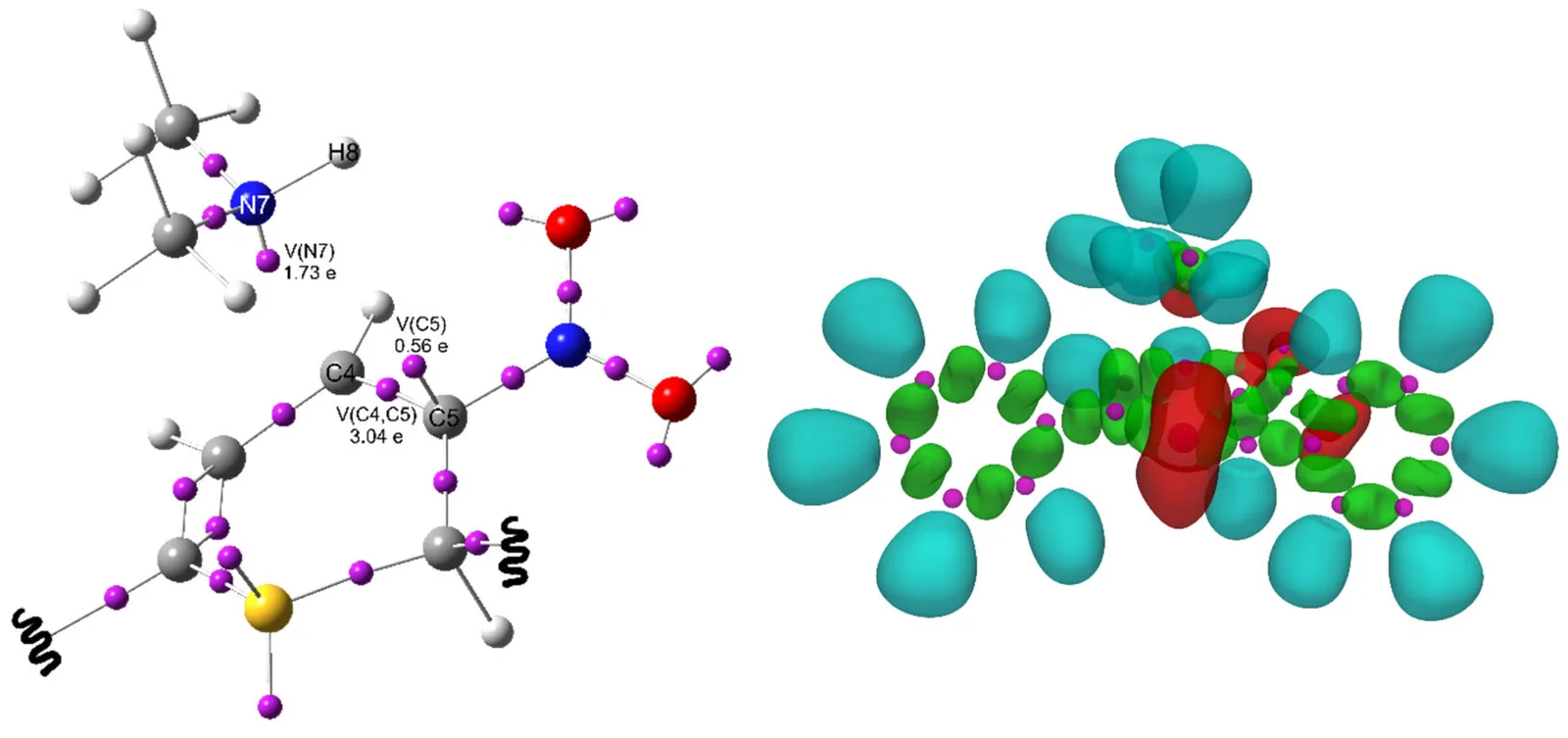
ELF results for **TS2E** as computed via ωB97X-D/6-311G(d,p) (PCM) calculations. To the right: topology of the ELF function, isovalue 0.75. Monosynaptic valence basins are given in red, disynaptic in green, protonated basins in cyan, and core basins in magenta. To the left: positions of ELF attractors (in magenta) and populations of significant basins. For clarity, some parts of the system are omitted.

**Figure 12:**
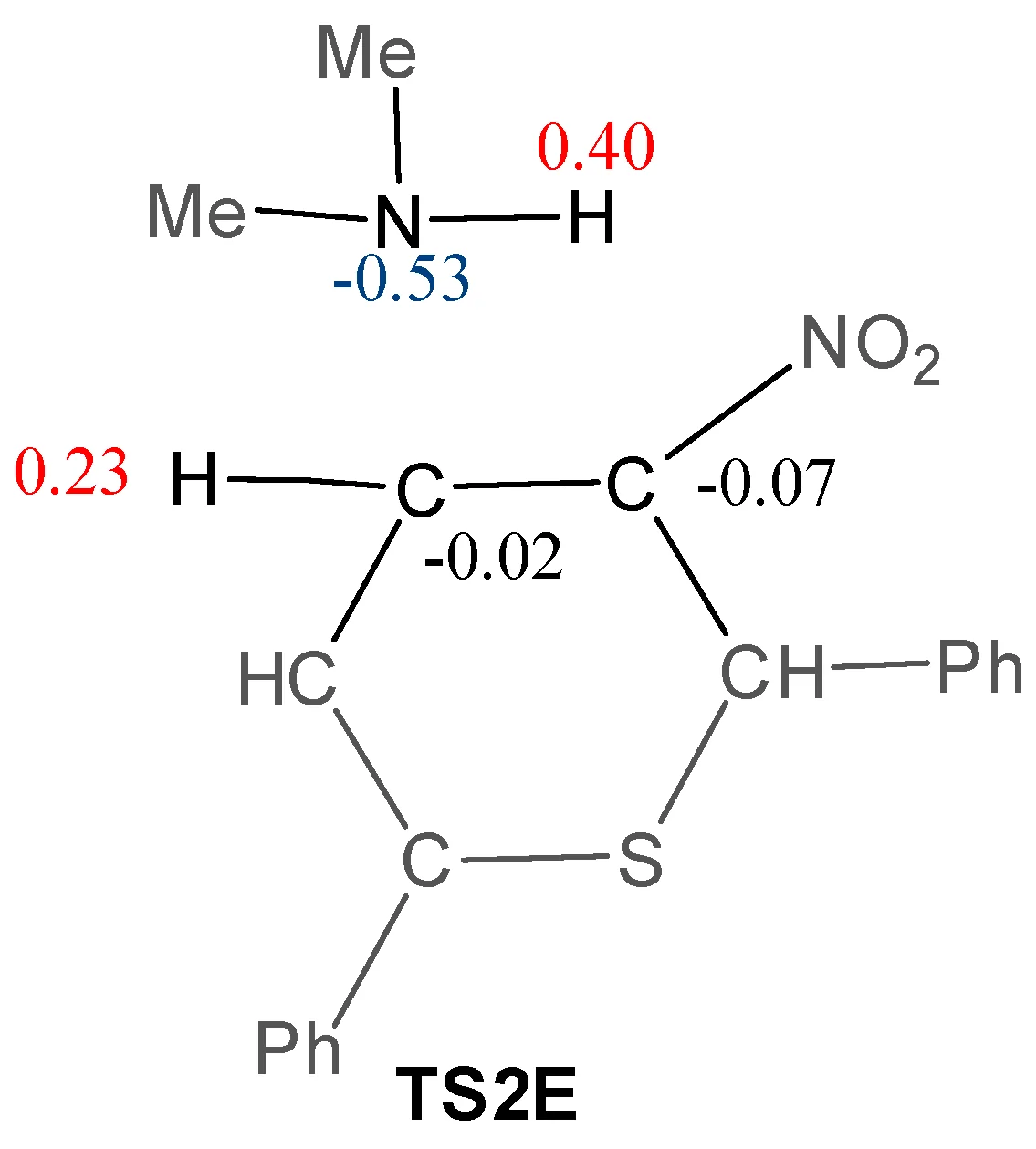
Natural charges of significant atoms (in electrons) in the **TS2E** as computed at the ground state via ωB97X-D/6-311G(d,p) (PCM) quantum chemical calculations; charges > 0.2 e are given in red, charges < −0.2 e are given in blue. For clarity, bond order and bond lengths were neglected; insignificant parts are shown in gray and abbreviated.

**Figure 13:**
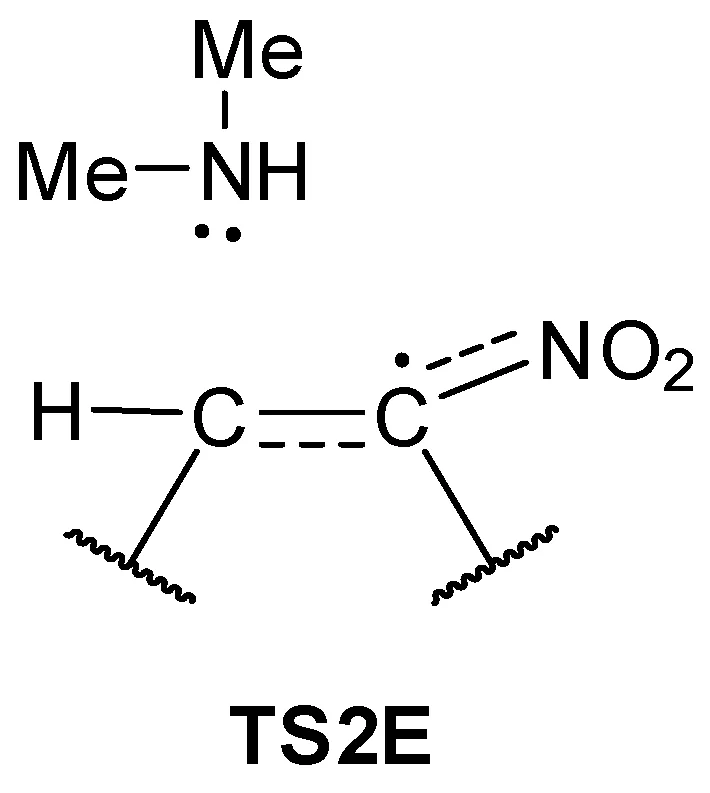
Lewis-like structure of **TS2E**. For clarity, insignificant parts are omitted.

The **TS2E** was analyzed by ELF (Figure 11). The irreducible valence basin V(C4,C5) with population of 3.04 e signifies the presence of the 1.05 order bond. In the NHMe_2_ region there is no region between the C4 and N7 atoms. The region that was responsible for the C4–N7 single bond in the **TS1E** is now a monosynaptic basin V(N7) with population of 1.73 e representing a lone pair of the nitrogen atom. The C5 atom has a monosynaptic valence basin V(C5) containing 0.56 e. According to the NPA (Figure 12), the charge of the C5 carbon atom integrates to −0.07, leading us to interpret the **TS2E** as a partially recombined pseudoradical (Figure 13).

**Figure 14:**
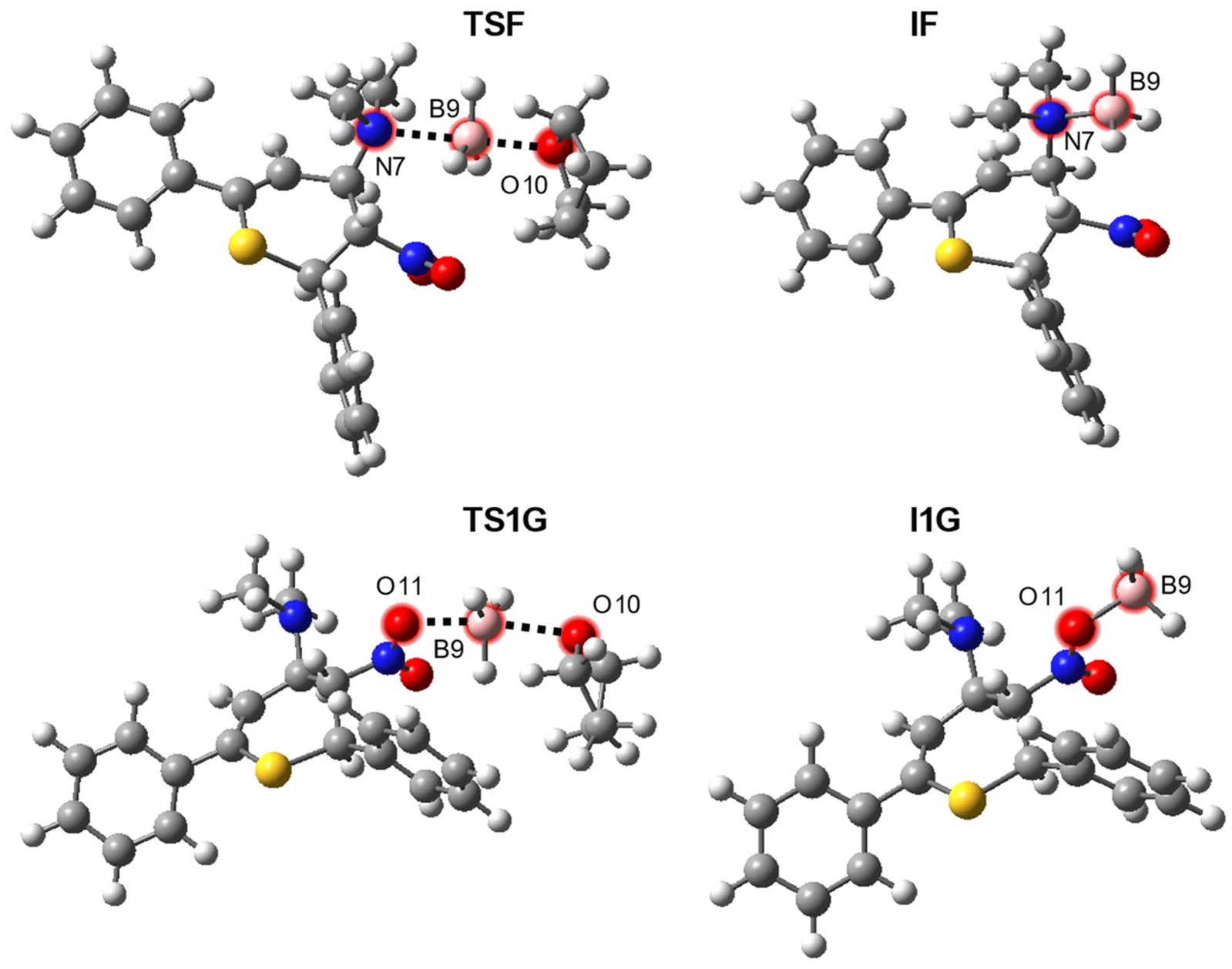
Views of critical structures of substitution reactions on the boron atom illustrated in Scheme 4 in the DCM solution according to ωB97XD/6-311G(d) (PCM) quantum chemical calculations.

Obtained parameters of the activation for the considered deamination process show that this process should be treated as allowed under considered conditions. This conclusion correlates well with generally known experimental observations regarding these types of processes [39,40,41]. In particular, it is evident that these processes are rather difficult from the kinetic point of view. In the light of our study, this is a consequence of energetical aspects of the first step of the deamination process, realized according to the [1.3]-sigmatropic shift mechanism. Therefore, we decided to analyze the influence of catalytic impact of the boron hydride on the reaction course. Earlier, we explored several different types of the LA catalyzed processes with the participation of the BH_3_ [27,63,64]. Within this analysis, the THF·BH_3_ complex was used as a source of the boron hydride, because it is a very popular synthon [65,66,67,68]. We detected two nucleophilic centers, which potentially can be considered good boron hydride acceptor: the nitrogen atom of the dimethylamino group and the oxygen atom of the nitro group. The transmission of the BH_3_ segment from the THF·BH_3_ complex is achieved as single-step process (Figure 14), and requires the enthalpy of activation at the level of several kcal/mol. In the case of the first considered reaction (transmission of the BH_3_ segment on the nitrogen atom of the dimethylamino group), within the localized transition state (**TSF**), one new single bond is formed between the N7 nitrogen atom and the B9 boron atom. In the case of the second considered reaction (transmission of the BH_3_ segment on the oxygen atom of the nitro group via **TSG1**), one new single bond is formed between the O11 oxygen atom and the B9 boron atom. In both cases, the formation of one new bond is conjugated with the dissociation of the bond between the B9 boron atom and the oxygen O10 atom from the THF segment. Therefore, from the structural point of view, the localized transition state is very similar to well-known transition states in the SN2 substitution reactions realized at the sp_3_ carbon atom [69,70,71]. Similar structures were localized earlier in the case of reaction between the THF·BH_3_ complex and the trimethylphosphine [72]. The electronic structures of the localized TSs were explored regarding the BH_3_ substitution, and **TS1G** was chosen as a model transition state.

**Scheme 4:**
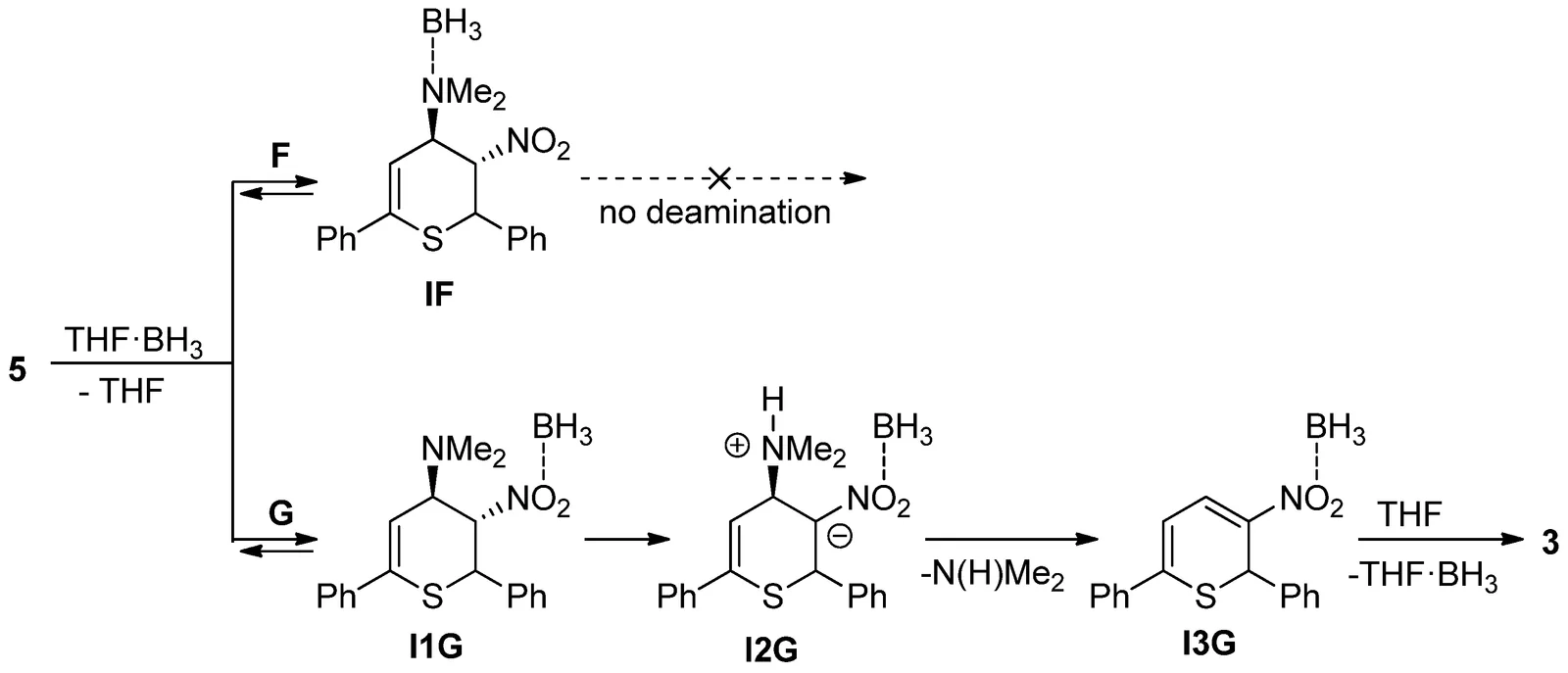
The scheme of the LA-catalyzed deamination of the adduct **3**.

**Figure 15:**
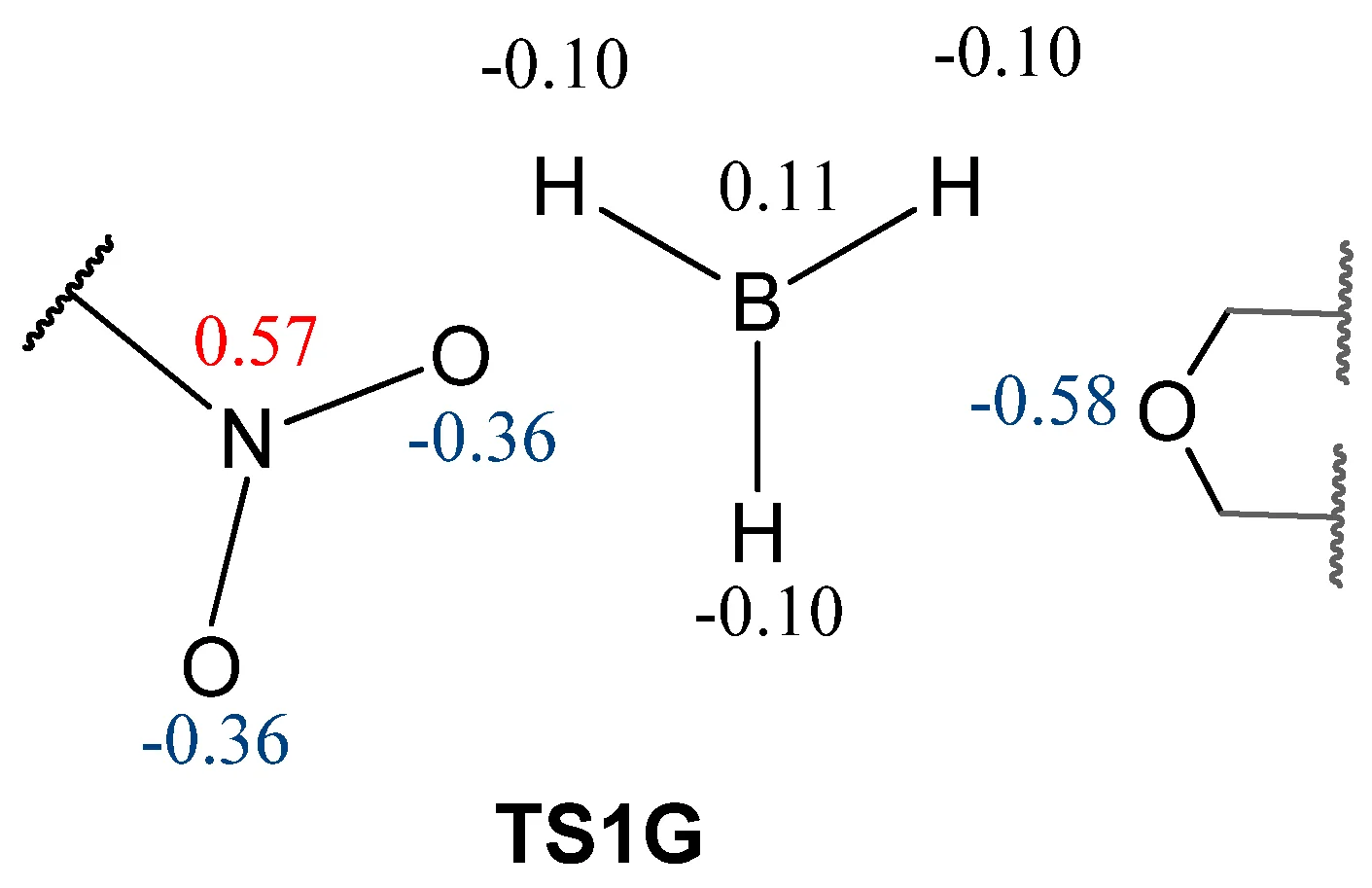
Natural charges of significant atoms (in electrons) in the **TS1G** as computed at the ground state via ωB97X-D/6-311G(d,p) (PCM) quantum chemical calculations; charges > 0.2 e are given in red, charges < −0.2 e are given in blue. For clarity, bond order and bond lengths were neglected; insignificant parts are shown in gray and abbreviated. For clarity, some parts of the system are omitted.

**Figure 16:**
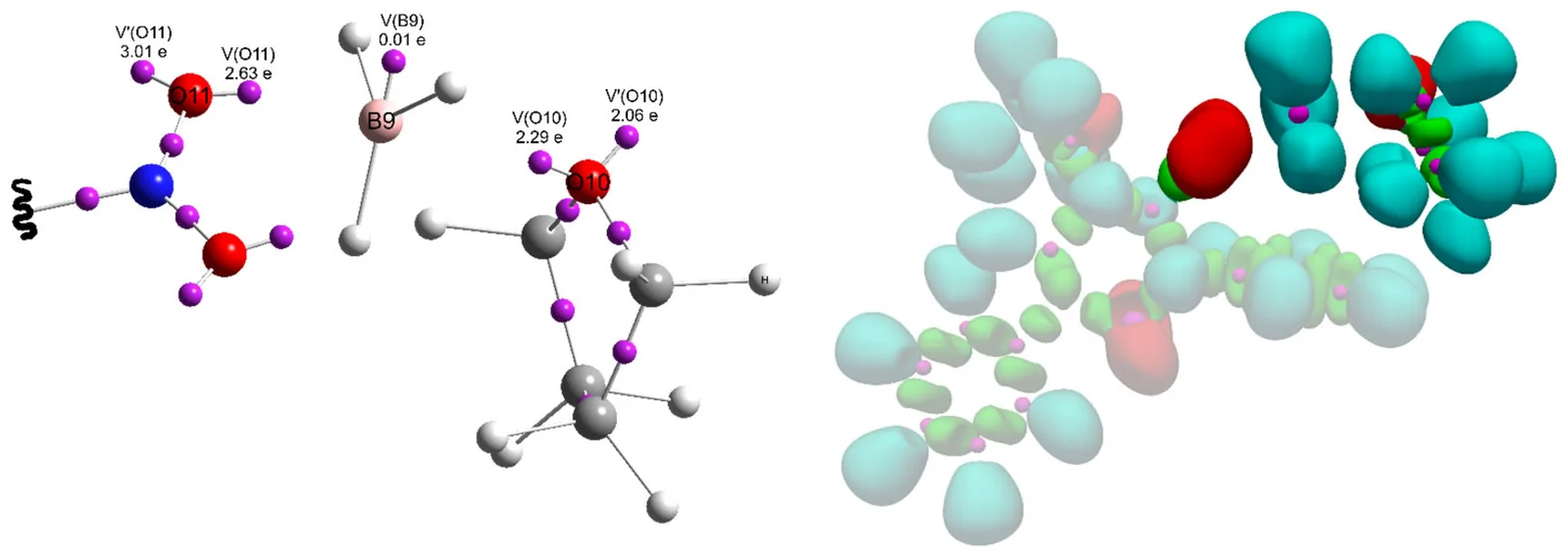
ELF results for **TS1G** as computed via ωB97X-D/6-311G(d,p) (PCM) calculations. To the right: topology of the ELF function, isovalue 0.75, and the plot of ELF surrounding the B9 atom, at isovalue of 0.1, with led monosynaptic basin visible. Monosynaptic valence basins are given in red, disynaptic in green, protonated basins in cyan, and core basins in magenta. To the left: positions of ELF attractors (in magenta) and populations of significant basins.

Only the active site in the **TS1G** was considered (Figure 15 and Figure 16). The boron atom (B9) has a negligible positive charge of 0.11, while both O10 and O11 oxygen atoms are negatively charged (−0.58 and −0.36, respectively) (Figure 14). In the ELF analysis of the **TS1G** it can be seen that both O10 and O11 atoms have monosynaptic basins directed at the B9 atom (V(O10) = 2.29 e; V(O11) = 2.63 e), while the boron atom has an almost monosynaptic basin (V(B9) = 0.01 e) not visible at the 0.75 ELF isovalue plot, but barely visible at ELF isovalue 0.1 (Figure 16). The attractor of the V(B9) basin is clearly visible though (Figure 15), leading us to believe that, here, the interaction between a lone pair of oxygen atoms and an “empty orbital” of the boron atom is observed via ELF and DFT methods.

Although the formation of both **IF** and **I1G** complexes is fast from the kinetic point of view, their ability to deaminate is completely different. In particular, in the case of the first complex, we did not detect any reaction channels connected with the deamination processes. In contrast, in the case of the **I1G** complexes, we optimized the reaction path leading to the target molecule **3**. This process occurs as a cascade reaction with three transition states.

**Figure 17:**
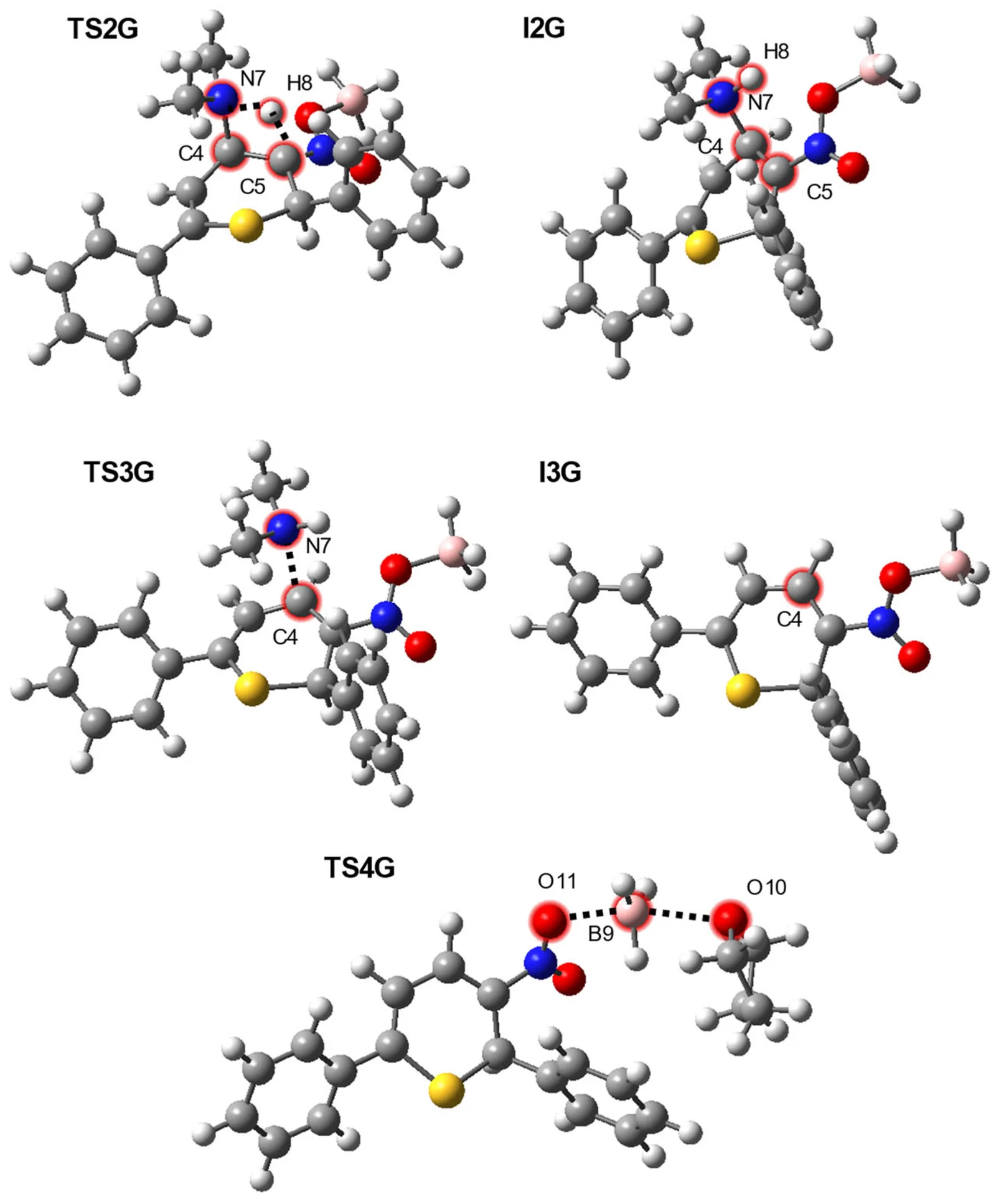
Views of critical structures of transformation of the **I1G** intermediate into target product (see Scheme 4) in the DCM solution according to ωB97XD/6-311G(d) (PCM) quantum chemical calculations.

The first stage of the transformation of the **I1G** into **3** is the hydrogen [1.3]-sigmatropic shift process. Similarly to the case of non-catalyzed process, within this stage, the C5–H8 single bond is broken. At the same time, the new N7–H8 single bond is formed. However, the enthalpy of activation for this transformation is almost 6 kcal/mol lower in comparison to the non-catalyzed reaction. As a result of this transformation, the **I2G** intermediate is formed. This was confirmed by the IRC calculations. The further conversion of the **I2G** intermediate is realized via the **TS3G** transition state. This transformation is associated with the dissociation of the dimethylamine molecule from the **I2G** intermediate. The obtained **I3G** intermediate can convert to the target molecule by transferring the boron hydride segment to a THF molecule. This process is achieved according to the description above of the SN_2_-like mechanism via **TS4G** transition state (Figure 17).

## 3. Computational Details

The exploration of the reaction profiles was performed on the basis of the quantum chemical DFT calculations. The ωB97XD/6-311G(d) level of theory from the Gaussian 16 software package [73,74] was used. The same level of theory was used in our research group regarding other, different types of addition, elimination, and rearrangement processes [35,36,75,76]. In all cases, satisfactory correlation between experimental results and quantum chemical calculations were obtained. Parallelly, however, analogous calculations were performed using the 6-311 + G(d,p) basis set for model process of the deamination of **5**, because diffuse and polarization function can be important for describing anions, zwitterions, and highly polar transition states, including proton-transfer stages. It was found that differences between Gibbs free energies of the activation of the analyzed path obtained via ωB97XD/6-311G(d) and ωB97XD/6-311 + G(d,p) do not exceed 1–1.5 kcal/mol. At the same time, the qualitative nature of Gibbs free energy profiles are identical (Figure S1 in the Supplementary Materials). Next, we compared key geometrical parameters for critical structures. This analysis (Table S1 in the Supplementary Materials) does not exhibit an important difference between data from ωB97XD/6-311G(d) and ωB97XD/6-311 + G(d,p). Therefore, we concluded that the application of the ωB97XD/6-311G(d) level of theory is satisfactorily justified, and is more attractive taking into account the computation time and the cost of computing power. 

All localized and optimized stationary points were characterized using vibrational analysis. It was found that starting molecules, intermediates, and products had positive Hessian matrices. On the other hand, all transition states (**TS**) showed only one negative eigenvalue in their Hessian matrices. The calculations were performed for T = 297K. Transition states are described as **TS**, pre-reaction complexes as **MC**, and reaction intermediates as **I**.

Intrinsic reaction coordinate (IRC) calculations were performed for the verification of all localized transition states. The presence of the solvent in the reaction environment (DCM) was included using the IEFPCM algorithm [77]. The global electron density transfer (GEDT) [78] was calculated according to the following formula (1):

GEDT = −ΣqA

where qA is the net charge, and the sum is taken over all the atoms of nitroalkene.

The same level of theory was used within ELF analysis.

Global electronic properties of reactants were estimated according to the equations recommended earlier by Parr and Domingo [79,80,81]. According to Domingo’s recommendation, for this purpose, the ωB97XD/6-311G(d) level of theory was used. All molecules were fully optimized. Next, the electronic chemical potentials (μ) and chemical hardness (η) were evaluated in terms of one-electron energies of FMO (E_HOMO_ and E_LUMO_) using the following equations:

Μ ≈ (E_HOMO_ + E_LUMO_)/2 η ≈ E_LUMO_ − E_HOMO_

Next, the values of µ and η were then used for the calculation of the global electrophilicity index (ω) according to the following formula:

ω = μ^2^/2η

Subsequently, global nucleophilicity (N) [82] can be expressed with the following equation:

N = E_HOMO_ − E_HOMO (tetracyanoethene)_

The local electrophilicity (ω_k_) condensed to atom *k* was calculated by projecting the index ω onto any reaction center *k* in the molecule using Parr functions P^+^_k_ [83]:

ω_k_ = P^+^_k_·ω

The local nucleophilicity (N_k_) condensed to atom *k* was calculated using global nucleophilicity N and Parr functions P^−^_k_ [83] according to the following formula:

N_k_ = P^−^_k_·N

## 4. Conclusions

The ωB97XD/6-311G(d) (PCM) quantum chemical calculations shed light on the molecular mechanism of the reaction between tioanalogs of the butadiene generated in situ with the participation of the *Lawesson* reagent and 2-phenylnitroethene. The first reaction stage is a (4 + 2) polar cycloaddition process. It was found, however, that only one of four theoretically possible cycloaddition reaction paths can be achieved. Additionally, the cycloaddition process is realized not via the “classical” single-step mechanism, but via the stepwise one, with the participation of the zwitterionic intermediate. Next, the mechanism of elimination of the N,N-dimethylamine from the primary cycloadduct was also examined. We found that this transformation is also carried out via the atypical scheme, according to the stepwise mechanism. Lastly, we analyzed the Lewis-acid promoted catalyst effect on the elimination course. In general, the title reaction should be classified as a rare-type, of the domino, multi-step processes with the participation of the stepwise cycloaddition reaction and the stepwise elimination process.

## Figures and Tables

**Table 1:** Kinetic and thermodynamic parameters for the **1** + **2** HDA reaction in the DCM solution according to ωB97XD/6-311G(d) (PCM) quantum chemical calculations (∆H, ∆G are in kcal/mol; ∆S are in cal/molK.

Path	Transition	∆H	∆S	∆G
**A**	**1 + 2**→**MCA**	−10.0	−39.4	1.7
	**1 + 2**→**TS1A**	2.2	−52.5	17.9
	**1 + 2**→**IA**	2.6	−50.9	17.8
	**1 + 2**→**TS2A**	2.7	−52.5	18.3
	**1 + 2**→**4**	−24.3	−53.1	−8.5
**B**	**1 + 2**→**MCB**	−20.3	−41.6	−7.9
	**1 + 2**→**TS1B**	−5.1	−53.1	10.7
	**1 + 2**→**IB**	−3.6	−52.6	12.0
	**1 + 2**→**TS2B**	−3.6	−52.6	12.1
	**1 + 2**→**5**	−27.9	−50.0	−13.0
**C**	**1 + 2**→**MCC**	−9.6	−40.8	2.5
	**1 + 2**→**TSC**	13.9	−52.9	29.6
	**1 + 2**→**6**	−19.6	−53.2	−3.8
**D**	**1 + 2**→**MCD**	−10.6	−44.9	2.8
	**1 + 2**→**TSD**	12.6	−52.3	28.2
	**1 + 2**→**7**	−22.6	−54.4	−6.4

**Table 2:** Key parameters for critical structures of the **1** + **2** HDA reaction in the DCM solution according to ωB97XD/6-311G(d) (PCM) quantum chemical calculations.

Path	Structure	Interatomic Distances [Å]						GEDT [e]
		S1–C2	C2–C3	C3–C4	C4–C5	C5–C6	C6–S1	
	**1**	1.691	1.403	1.396				
	**2**					1.332		
**A**	**MCA**	1.692	1.402	1.397	3.684	1.333	3.665	
	**TS1A**	1.740	1.363	1.436	2.913	1.412	2.167	−0.50
	**IA**	1.763	1.352	1.449	2.801	1.448	1.985	−0.66
	**TS2A**	1.767	1.346	1.460	2.399	1.471	1.924	−0.60
	**4**	1.773	1.335	1.505	1.562	1.528	1.832	
**B**	**MCB**	1.693	1.409	1.385	3.762	1.334	3.901	
	**TS1B**	1.754	1.364	1.424	2.802	1.445	1.987	−0.48
	**IB**	1.756	1.357	1.437	2.362	1.460	1.958	−0.55
	**TS2B**	1.756	1.357	1.437	2.362	1.460	1.958	−0.55
	**5**	1.776	1.335	1.508	1.530	1.532	1.830	
**C**	**MCC**	1.689	1.403	1.396	3.241	1.330	3.497	
	**TSC**	1.734	1.360	1.439	2.370	1.423	2.071	−0.40
	**6**	1.775	1.333	1.505	1.563	1.526	1.825	
**D**	**MCD**	1.689	1.404	1.396	3.473	1.333	3.469	
	**TSD**	1.724	1.361	1.438	2.237	1.409	2.172	−0.31
	**7**	1.777	1.333	1.502	1.563	1.546	1.784	

**Table 3:** Kinetic and thermodynamic parameters for different schemes of the deamination of **5** in the DCM solution according to ωB97XD/6-311G(d) (PCM) quantum chemical calculations (∆H, ∆G are in kcal/mol; ∆S are in cal/molK).

Path	Transition	∆H	∆S	∆G
**E**	**5**→**TS1E**	41.7	−3.3	42.7
	**5**→**IE**	15.9	−4.8	17.4
	**5**→**TS2E**	21.9	−2.4	22.6
	**5**→**3** + N(H)Me_2_	21.1	44.7	7.8
**F**	THF·BH_3_ + **5**→**TSF**	5.0	−43.4	18.0
	THF·BH_3_ + **5**→**IF** + THF	−4.7	−13.8	−0.6
**G**	THF·BH_3_ + **5**→**TS1G**	8.7	−39.9	20.6
	THF·BH_3_ + **5**→**I1G** + THF	11.2	−5.6	12.8
	**I1G**→**TS2G**	36.0	−5.1	37.5
	**I1G**→**I2G**	2.0	−6.0	3.8
	**I1G**→**TS3G**	13.2	−1.0	13.4
	**I1G**→**I3G** + N(H)Me_2_	19.7	43.4	6.7
	THF + **I3G**→**TS4G**	0.9	−31.6	10.3
	THF + **I3G**→**3** + THF·BH_3_	−9.7	6.8	−11.7

## Data Availability

The original contributions presented in this study are included in the article/supplementary material. Further inquiries can be directed to the corresponding author.

## Supplementary Materials

The following supporting information can be downloaded at: https://www.mdpi.com/article/10.3390/ijms26188948/s1.
