# Supplementary material for: On the Question of the Regio-Orientation, Stereo-Orientation and Molecular Mechanism in the Cascade Cycloaddition/Rearrangement/Elimination Processes Leading to Nitro-Substituted Thiopyran Analogs: DFT Computational Study
Source: Int J Mol Sci. 2025 Sep 14;26(18):8948. doi: 10.3390/ijms26188948 (PMC12469585; doi:10.3390/ijms26188948)
Supplement: Supplementary file 1 [file ijms-26-08948-s001.zip › ijms-3853341-supplementary.pdf]

SUPPLEMENTARY MATERIAL

for the manuscript

On the question of the regio-orientation, stereo-orientation and molecular mechanism in the cascade cycloaddition/rearrangement/elimination processes leading to nitro-substituted thiopyran analogs: DFT computational study

Mikołaj Sadowski [1], Ewa Dresler [2] and Radomir Jasiński [3,*]

[1] Cracow University of Technology, CUT Doctoral School, Faculty of Chemical Engineering and Technology Warszawska 24, 31-155 Kraków, Poland
[2] Łukasiewicz Research Network—Institute of Heavy Organic Synthesis "Blachownia", Energetyków 9, 47-225 Kędzierzyn-Koźle, Poland
[3] Cracow University of Technology, Department of Organic Chemistry and Technology, Warszawska 24, 31-155 Kraków, Poland

* Correspondence: radomir.jasinski@pk.edu.pl

Table S1. The comparison of key geometrical parameters of critical structures for the process of deamination of **5** in the DCM solution according to ωB97XD/6-311G(d) (PCM) and ωB97XD/6-311+G(d,p) (PCM) quantumchemical calculations (all distances are in Å; numeration of atoms is presented on the Figure 6).

Distance	ωB97XD/6-311G(d)				ωB97XD/6-311+G(d,p)			
	**5**	**TS1E**	**IE**	**TS2E**	**5**	**TS1E**	**IE**	**TS2E**
C4-C5	1.530	1.528	1.490	1.395	1.530	1.529	1.493	1.396
C4-N7	1.462	1.548	1.551	2.030	1.463	1.546	1.548	2.040
N7-H8	2.674	1.305	1.038		2.683	1.310	1.041	
C5-H8	1.088	1.443	2.450		1.088	1.430	2.452	

Figure S1. Gibbs free energy profile for the process of deamination of **5** in the DCM solution according to ωB97XD/6-311+G(d,p) (PCM) quantumchemical calculations.

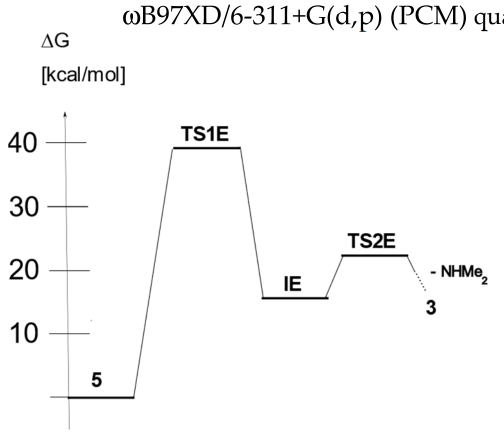

Cartesian coordinates and energetical parameters for critical structures:

1

Zero-point correction= 0.217432 (Hartree/Particle)
Thermal correction to Energy= 0.230052
Thermal correction to Enthalpy= 0.230996
Thermal correction to Gibbs Free Energy= 0.177538
Sum of electronic and zero-point Energies= -879.634814
Sum of electronic and thermal Energies= -879.622194
Sum of electronic and thermal Enthalpies= -879.621250
Sum of electronic and thermal Free Energies= -879.674708

Center Number	Atomic Number	Atomic Type	Coordinates (Angstroms) X	Y	Z
1	6	0	-0.007428	0.096152	-0.312201
2	6	0	-0.778456	-1.015076	0.060229
3	6	0	-2.161043	-1.205060	0.062477
4	1	0	-2.510026	-2.221549	-0.106918
5	1	0	-0.228002	-1.928672	0.267041
6	16	0	-0.545464	1.482624	-1.116199
7	6	0	1.464367	-0.042874	-0.072349
8	6	0	2.164705	0.958018	0.605099
9	6	0	2.162866	-1.167079	-0.519254
10	6	0	3.525630	0.827941	0.847124
11	1	0	1.633365	1.839316	0.947294
12	6	0	3.530139	-1.285452	-0.296718
13	1	0	1.641509	-1.943091	-1.070259
14	6	0	4.215022	-0.292478	0.393473
15	1	0	4.051299	1.608138	1.387628
16	1	0	4.060236	-2.156804	-0.666748
17	1	0	5.280564	-0.387928	0.573205
18	7	0	-3.133791	-0.350178	0.319080
19	6	0	-4.525596	-0.723403	0.098317
20	1	0	-4.987628	-0.012239	-0.589001
21	1	0	-4.583564	-1.718103	-0.338662
22	1	0	-5.071970	-0.707533	1.043707
23	6	0	-2.924301	0.942881	0.942392
24	1	0	-3.738430	1.112085	1.649007
25	1	0	-1.974997	0.939402	1.475022
26	1	0	-2.903819	1.744825	0.202775

2

Zero-point correction= 0.138127 (Hartree/Particle)
Thermal correction to Energy= 0.147147
Thermal correction to Enthalpy= 0.148091
Thermal correction to Gibbs Free Energy= 0.102509
Sum of electronic and zero-point Energies= -513.959172
Sum of electronic and thermal Energies= -513.950152
Sum of electronic and thermal Enthalpies= -513.949208
Sum of electronic and thermal Free Energies= -513.994789

Center Number	Atomic Number	Atomic Type	Coordinates (Angstroms) X	Y	Z
1	6	0	0.615153	-0.448497	0.000048
2	1	0	0.913257	-1.492996	0.000112
3	6	0	1.593350	0.455485	-0.000009
4	1	0	1.502657	1.530838	-0.000083
5	6	0	-0.820327	-0.174794	0.000027
6	6	0	-1.349860	1.122877	0.000051
7	6	0	-1.700111	-1.262669	-0.000020
8	6	0	-2.720962	1.319701	0.000024
9	1	0	-0.693250	1.985839	0.000098
10	6	0	-3.074339	-1.063658	-0.000050
11	1	0	-1.301746	-2.272234	-0.000034
12	6	0	-3.586714	0.227828	-0.000028
13	1	0	-3.119315	2.328119	0.000046
14	1	0	-3.743388	-1.916776	-0.000088
15	1	0	-4.659448	0.387160	-0.000048
16	7	0	2.979279	0.032901	0.000003
17	8	0	3.251739	-1.154835	0.000045
18	8	0	3.811904	0.925099	-0.000081

MCA

Zero-point correction= 0.357210 (Hartree/Particle)
Thermal correction to Energy= 0.380461
Thermal correction to Enthalpy= 0.381405
Thermal correction to Gibbs Free Energy= 0.301067
Sum of electronic and zero-point Energies= -1393.610587
Sum of electronic and thermal Energies= -1393.587336
Sum of electronic and thermal Enthalpies= -1393.586392
Sum of electronic and thermal Free Energies= -1393.666730

Center Number	Atomic Number	Atomic Type	Coordinates (Angstroms) X	Y	Z
1	6	0	-0.580844	-1.409500	-0.004933
2	6	0	-1.442079	-0.996098	-1.031591
3	6	0	-2.763400	-0.545688	-0.984402
4	1	0	-3.033116	0.209693	-1.720057
5	1	0	-0.978801	-0.815020	-1.996410
6	16	0	-0.918801	-1.433384	1.652879
7	6	0	0.823496	-1.712718	-0.428976
8	6	0	1.505116	-2.809783	0.103682
9	6	0	1.497875	-0.883805	-1.330159
10	6	0	2.815511	-3.079427	-0.266604
11	1	0	0.997628	-3.454689	0.812002
12	6	0	2.817702	-1.137364	-1.679874
13	1	0	1.007259	-0.002093	-1.726781
14	6	0	3.480040	-2.240182	-1.155485
15	1	0	3.322513	-3.944886	0.147264
16	1	0	3.331668	-0.460689	-2.353986
17	1	0	4.510035	-2.441303	-1.430691
18	7	0	-3.768902	-0.901702	-0.211121
19	6	0	-4.990960	-0.106175	-0.193223
20	1	0	-5.259620	0.105447	0.842840
21	1	0	-4.825655	0.840725	-0.702383
22	1	0	-5.807860	-0.654539	-0.668728
23	6	0	-3.818752	-2.143405	0.538909
24	1	0	-4.803704	-2.588035	0.379129
25	1	0	-3.050880	-2.821893	0.175167
26	1	0	-3.660778	-1.971500	1.603537
27	6	0	-0.625217	2.389378	-0.363705
28	6	0	0.121497	1.932459	0.642015
29	1	0	-0.289953	2.705545	-1.339764
30	1	0	-0.395416	1.587558	1.531993
31	7	0	-2.063330	2.414861	-0.252134
32	8	0	-2.599188	2.148917	0.808973
33	8	0	-2.680563	2.690074	-1.273997

34	6	0	1.574330	1.789325	0.613850
35	6	0	2.170984	0.912839	1.525582
36	6	0	2.380083	2.452252	-0.319964
37	6	0	3.538290	0.681021	1.488216
38	1	0	1.548388	0.383223	2.238822
39	6	0	3.746505	2.225102	-0.350087
40	1	0	1.942655	3.158308	-1.017978
41	6	0	4.327250	1.333781	0.548856
42	1	0	3.986130	-0.015471	2.188050
43	1	0	4.363150	2.744956	-1.075060
44	1	0	5.396251	1.152711	0.517305

Harmonic frequencies (cm**-1), IR intensities (KM/Mole), Raman scattering activities (A**4/AMU), depolarization ratios for plane and unpolarized incident light, reduced masses (AMU), force constants (mDyne/A), and normal coordinates:

		1			2			3	
		A			A			A	
Frequencies --		4.9134			31.9113			37.1767	
Red. masses --		6.1848			6.4007			4.8734	
Frc consts --		0.0001			0.0038			0.0040	
IR Inten --		1.4813			0.2047			1.5466	
Atom	AN	X	Y	Z	X	Y	Z	X	Y	Z
1	6	0.05	-0.02	-0.02	0.08	0.02	0.03	-0.03	0.02	0.03
2	6	0.08	-0.04	-0.06	0.09	0.09	0.05	0.00	0.09	0.03
3	6	0.09	0.00	-0.11	0.08	0.07	0.08	-0.01	0.05	0.02
4	1	0.13	-0.03	-0.15	0.06	0.11	0.13	-0.01	0.11	0.09
5	1	0.10	-0.10	-0.06	0.08	0.14	0.06	0.03	0.17	0.06
6	16	0.01	0.04	-0.03	0.09	-0.03	0.03	-0.08	-0.06	0.02
7	6	0.05	-0.05	0.01	0.07	-0.03	0.00	-0.02	0.01	0.05
8	6	0.03	-0.04	0.05	0.02	-0.08	-0.04	-0.05	0.00	0.08
9	6	0.08	-0.07	0.01	0.09	-0.04	0.01	0.01	-0.01	0.06
10	6	0.03	-0.07	0.08	-0.00	-0.14	-0.07	-0.04	-0.02	0.11
11	1	0.01	-0.02	0.05	0.00	-0.08	-0.05	-0.07	0.01	0.07
12	6	0.09	-0.09	0.05	0.07	-0.10	-0.02	0.01	-0.03	0.10
13	1	0.10	-0.07	-0.01	0.13	-0.01	0.04	0.04	-0.00	0.04
14	6	0.06	-0.09	0.08	0.03	-0.15	-0.06	-0.01	-0.03	0.12
15	1	0.02	-0.06	0.11	-0.04	-0.18	-0.11	-0.07	-0.02	0.13
16	1	0.11	-0.11	0.04	0.09	-0.11	-0.01	0.04	-0.04	0.10
17	1	0.07	-0.11	0.10	0.01	-0.19	-0.09	-0.01	-0.05	0.15
18	7	0.07	0.09	-0.10	0.08	-0.02	0.04	-0.03	-0.07	-0.05
19	6	0.10	0.14	-0.16	0.06	-0.06	0.09	-0.06	-0.13	-0.02
20	1	0.05	0.15	-0.18	0.08	-0.08	0.10	-0.10	-0.24	-0.01
21	1	0.17	0.14	-0.15	0.00	-0.06	0.09	-0.09	-0.08	0.07
22	1	0.10	0.18	-0.21	0.06	-0.09	0.11	-0.02	-0.13	-0.09
23	6	-0.00	0.13	-0.04	0.13	-0.07	-0.03	0.01	-0.15	-0.17
24	1	-0.02	0.16	-0.05	0.13	-0.08	-0.03	0.03	-0.16	-0.21

25	1	-0.02	0.08	0.01	0.13	-0.03	-0.09	0.03	-0.09	-0.24
26	1	-0.03	0.17	-0.04	0.16	-0.13	-0.02	0.02	-0.24	-0.16
27	6	-0.10	-0.03	0.07	-0.07	0.16	-0.02	0.03	0.11	0.03
28	6	-0.05	0.00	0.05	-0.09	0.08	-0.04	0.04	0.00	-0.03
29	1	-0.15	-0.07	0.04	-0.05	0.22	0.01	0.01	0.22	0.07
30	1	-0.01	0.04	0.09	-0.12	0.03	-0.07	0.06	-0.11	-0.06
31	7	-0.09	-0.01	0.15	-0.07	0.16	-0.05	0.03	0.07	0.05
32	8	-0.04	0.04	0.19	-0.09	0.09	-0.07	0.04	-0.05	0.03
33	8	-0.15	-0.05	0.18	-0.05	0.22	-0.04	0.01	0.15	0.08
34	6	-0.05	-0.00	-0.01	-0.10	0.03	-0.01	0.04	0.02	-0.06
35	6	-0.01	0.01	-0.03	-0.14	0.04	0.02	0.06	-0.03	-0.12
36	6	-0.09	-0.02	-0.06	-0.06	-0.02	-0.02	0.02	0.08	-0.03
37	6	-0.01	0.02	-0.08	-0.15	-0.02	0.05	0.07	-0.02	-0.16
38	1	0.03	0.02	0.01	-0.17	0.08	0.03	0.08	-0.08	-0.14
39	6	-0.09	-0.01	-0.11	-0.07	-0.08	0.01	0.02	0.09	-0.07
40	1	-0.13	-0.03	-0.05	-0.02	-0.03	-0.05	0.01	0.12	0.02
41	6	-0.05	0.01	-0.12	-0.12	-0.08	0.04	0.05	0.04	-0.13
42	1	0.02	0.03	-0.09	-0.19	-0.02	0.07	0.08	-0.06	-0.21
43	1	-0.13	-0.02	-0.15	-0.04	-0.13	-0.00	0.01	0.13	-0.05
44	1	-0.05	0.01	-0.17	-0.12	-0.13	0.05	0.05	0.05	-0.16

	4	5	6
	A	A	A
Frequencies --	52.7717	58.5158	63.7505
Red. masses --	4.2595	5.3151	5.0861
Frc consts --	0.0070	0.0107	0.0122
IR Inten --	3.8956	0.6378	7.5873

Atom	AN	X	Y	Z	X	Y	Z	X	Y	Z
1	6	0.00	-0.09	0.01	0.01	-0.01	-0.00	0.02	0.09	0.01
2	6	0.00	-0.07	0.02	0.01	-0.02	0.00	0.01	0.04	-0.01
3	6	0.02	-0.01	0.02	0.01	0.00	0.02	-0.00	-0.01	-0.02
4	1	0.06	-0.02	0.00	0.02	0.02	0.02	-0.04	-0.04	-0.03
5	1	0.00	-0.10	0.02	-0.00	-0.01	-0.00	0.01	0.01	-0.02
6	16	0.02	-0.11	0.02	-0.01	-0.05	-0.01	0.03	0.19	0.01
7	6	0.00	-0.08	0.00	0.03	0.05	0.01	0.01	0.05	0.01
8	6	0.01	-0.09	-0.03	0.12	0.14	0.10	-0.02	0.03	0.02
9	6	0.00	-0.04	0.04	-0.02	0.02	-0.05	0.03	0.01	-0.01
10	6	0.02	-0.06	-0.02	0.14	0.22	0.14	-0.04	-0.02	-0.00
11	1	0.01	-0.12	-0.06	0.16	0.16	0.16	-0.04	0.06	0.03
12	6	0.01	-0.00	0.05	-0.00	0.09	-0.02	0.01	-0.04	-0.03
13	1	-0.00	-0.03	0.07	-0.07	-0.04	-0.12	0.05	0.03	-0.02
14	6	0.02	-0.01	0.02	0.08	0.19	0.08	-0.02	-0.06	-0.03
15	1	0.02	-0.07	-0.05	0.21	0.29	0.22	-0.06	-0.04	0.00
16	1	0.01	0.03	0.09	-0.05	0.08	-0.07	0.03	-0.07	-0.04
17	1	0.03	0.01	0.03	0.10	0.25	0.10	-0.03	-0.10	-0.04
18	7	0.01	0.07	0.04	0.01	0.02	0.03	0.02	-0.04	-0.01
19	6	0.06	0.15	0.03	0.04	0.06	0.05	-0.03	-0.11	-0.03
20	1	0.12	0.27	0.02	0.09	0.10	0.06	-0.08	-0.16	-0.03

21	1	0.08	0.09	-0.08	0.04	0.04	0.02	-0.05	-0.08	0.01
22	1	0.00	0.15	0.12	0.00	0.07	0.10	0.02	-0.14	-0.08
23	6	-0.07	0.10	0.09	-0.02	0.01	0.01	0.09	-0.02	0.03
24	1	-0.08	0.14	0.08	-0.02	0.03	-0.02	0.10	-0.07	0.06
25	1	-0.09	0.05	0.13	-0.02	0.01	0.01	0.11	-0.00	0.03
26	1	-0.08	0.14	0.08	-0.04	-0.00	0.01	0.11	0.01	0.02
27	6	-0.01	0.04	-0.02	-0.02	0.01	-0.02	0.00	-0.05	0.04
28	6	-0.02	-0.04	-0.05	-0.03	-0.06	-0.04	-0.02	0.02	0.09
29	1	0.01	0.13	0.01	-0.01	0.04	-0.01	0.04	-0.16	0.01
30	1	-0.04	-0.13	-0.09	-0.05	-0.08	-0.06	-0.02	0.09	0.11
31	7	-0.01	0.00	-0.05	-0.02	0.04	-0.01	0.00	-0.02	-0.01
32	8	-0.02	-0.05	-0.07	-0.02	0.03	-0.02	-0.03	0.09	-0.00
33	8	0.00	0.03	-0.05	-0.02	0.09	-0.00	0.04	-0.12	-0.06
34	6	-0.02	0.01	-0.02	-0.04	-0.10	-0.04	-0.02	-0.00	0.07
35	6	-0.02	-0.09	-0.12	-0.07	-0.15	-0.07	-0.03	-0.15	-0.07
36	6	-0.01	0.16	0.09	-0.02	-0.09	-0.01	-0.02	0.12	0.16
37	6	-0.01	-0.04	-0.11	-0.08	-0.19	-0.08	-0.04	-0.20	-0.15
38	1	-0.02	-0.21	-0.21	-0.09	-0.17	-0.10	-0.03	-0.25	-0.14
39	6	-0.01	0.21	0.10	-0.02	-0.12	-0.01	-0.03	0.08	0.09
40	1	-0.02	0.24	0.17	0.01	-0.05	0.01	-0.02	0.25	0.29
41	6	-0.00	0.11	0.00	-0.05	-0.17	-0.04	-0.04	-0.08	-0.07
42	1	-0.01	-0.12	-0.19	-0.10	-0.23	-0.10	-0.04	-0.33	-0.27
43	1	0.00	0.33	0.19	-0.00	-0.12	0.01	-0.03	0.18	0.16
44	1	0.00	0.15	0.01	-0.06	-0.21	-0.05	-0.04	-0.12	-0.13

7 8 9

A A A

Frequencies -- 79.0157 85.2623 97.9752

Red. masses -- 3.6902 4.8179 5.9532

Frc consts -- 0.0136 0.0206 0.0337

IR Inten -- 3.4021 0.9287 2.3950

Atom	AN	X	Y	Z	X	Y	Z	X	Y	Z
1	6	-0.03	-0.01	-0.10	-0.01	-0.05	0.00	0.01	-0.05	0.04
2	6	-0.03	-0.03	-0.10	-0.00	-0.03	0.00	0.02	-0.02	0.04
3	6	-0.02	0.00	-0.03	0.00	-0.02	0.01	0.03	-0.01	0.03
4	1	-0.05	0.05	0.03	0.01	-0.01	0.01	0.05	0.02	0.05
5	1	-0.05	-0.04	-0.11	0.01	-0.01	0.01	0.03	0.01	0.06
6	16	-0.04	0.05	-0.10	-0.02	-0.09	0.00	-0.04	-0.13	0.03
7	6	-0.03	-0.03	-0.06	-0.01	-0.02	0.01	0.01	-0.02	0.04
8	6	-0.05	-0.02	-0.01	-0.03	-0.09	-0.11	0.08	0.04	0.09
9	6	0.01	-0.04	-0.04	0.02	0.10	0.15	-0.05	-0.07	-0.07
10	6	-0.02	-0.01	0.07	-0.01	-0.03	-0.08	0.06	0.03	0.02
11	1	-0.08	-0.01	-0.03	-0.06	-0.19	-0.22	0.14	0.09	0.18
12	6	0.04	-0.03	0.04	0.05	0.16	0.19	-0.08	-0.09	-0.15
13	1	0.02	-0.05	-0.08	0.02	0.13	0.22	-0.07	-0.10	-0.09
14	6	0.02	-0.01	0.10	0.03	0.10	0.07	-0.03	-0.04	-0.12
15	1	-0.04	0.00	0.12	-0.03	-0.09	-0.18	0.10	0.08	0.06
16	1	0.07	-0.04	0.06	0.08	0.26	0.31	-0.14	-0.15	-0.26

17	1	0.04	-0.00	0.17	0.05	0.15	0.11	-0.05	-0.06	-0.19
18	7	0.04	-0.02	0.04	-0.00	-0.01	0.01	0.00	-0.01	0.00
19	6	0.10	0.06	0.29	0.01	0.01	0.03	-0.00	-0.02	-0.06
20	1	0.34	0.11	0.34	0.00	-0.03	0.03	-0.19	-0.23	-0.06
21	1	0.05	0.04	0.23	0.05	0.03	0.07	0.09	0.09	0.16
22	1	-0.05	0.11	0.48	0.01	0.05	-0.01	0.08	0.08	-0.32
23	6	0.06	-0.09	-0.08	-0.03	-0.03	-0.02	-0.05	-0.06	-0.07
24	1	0.05	-0.07	-0.09	-0.03	-0.01	-0.04	-0.05	-0.03	-0.13
25	1	0.04	-0.06	-0.17	-0.04	-0.03	-0.03	-0.05	-0.05	-0.09
26	1	0.09	-0.19	-0.07	-0.03	-0.06	-0.01	-0.07	-0.12	-0.06
27	6	-0.01	-0.06	-0.03	0.02	0.01	0.01	0.01	0.00	-0.04
28	6	-0.01	-0.02	-0.01	0.01	0.13	0.07	0.01	0.03	-0.02
29	1	-0.01	-0.12	-0.05	0.03	-0.12	-0.03	-0.00	-0.10	-0.07
30	1	-0.00	0.03	0.01	0.02	0.24	0.11	0.01	0.10	0.00
31	7	-0.00	0.00	-0.00	0.02	0.02	-0.02	0.01	0.09	0.01
32	8	0.01	0.10	0.03	0.00	0.17	0.01	0.05	0.38	0.10
33	8	-0.02	-0.03	-0.01	0.03	-0.15	-0.07	-0.02	-0.13	-0.03
34	6	-0.01	-0.02	-0.01	0.01	0.10	0.04	0.00	-0.00	-0.03
35	6	0.00	-0.01	-0.00	-0.00	0.07	0.02	-0.01	-0.01	-0.03
36	6	-0.01	0.00	0.00	0.00	0.03	-0.01	0.02	0.04	0.01
37	6	0.01	0.03	0.02	-0.03	-0.05	-0.07	-0.00	0.03	0.02
38	1	0.00	-0.03	-0.02	0.00	0.13	0.07	-0.02	-0.03	-0.05
39	6	-0.00	0.04	0.03	-0.02	-0.08	-0.09	0.03	0.08	0.06
40	1	-0.02	-0.01	0.00	0.01	0.06	0.01	0.02	0.04	0.02
41	6	0.01	0.06	0.04	-0.03	-0.13	-0.13	0.02	0.08	0.07
42	1	0.02	0.04	0.03	-0.04	-0.08	-0.09	-0.01	0.03	0.03
43	1	-0.00	0.07	0.05	-0.02	-0.14	-0.14	0.04	0.11	0.10
44	1	0.01	0.10	0.07	-0.05	-0.23	-0.21	0.03	0.12	0.11

	10	11	12
	A	A	A
Frequencies --	108.0252	114.4896	122.6717
Red. masses --	4.5317	5.4994	5.0446
Frc consts --	0.0312	0.0425	0.0447
IR Inten --	2.3040	9.5649	0.4899

Atom	AN	X	Y	Z	X	Y	Z	X	Y	Z
1	6	0.02	0.03	0.04	-0.01	-0.06	-0.04	0.03	0.10	0.01
2	6	0.04	0.09	0.04	-0.04	-0.07	-0.02	0.02	0.26	0.07
3	6	0.04	0.08	0.02	-0.04	-0.05	0.01	-0.00	0.19	0.09
4	1	0.06	0.12	0.05	-0.04	-0.06	-0.00	-0.03	0.20	0.12
5	1	0.06	0.14	0.06	-0.07	-0.09	-0.04	0.02	0.39	0.09
6	16	-0.04	-0.08	0.03	0.05	0.01	-0.03	0.05	-0.06	0.01
7	6	0.02	0.05	0.05	-0.02	-0.07	-0.06	0.01	0.06	-0.03
8	6	0.03	0.07	0.08	-0.01	-0.06	-0.05	-0.05	0.01	-0.06
9	6	0.00	-0.01	-0.02	-0.01	-0.04	-0.02	0.06	0.05	0.01
10	6	0.01	0.02	0.02	0.03	-0.00	0.01	-0.05	-0.02	-0.01
11	1	0.06	0.11	0.14	-0.01	-0.09	-0.08	-0.10	-0.01	-0.11
12	6	-0.03	-0.06	-0.09	0.02	0.02	0.04	0.08	0.04	0.08

13	1	0.01	-0.01	-0.03	-0.03	-0.06	-0.04	0.10	0.07	-0.01
14	6	-0.03	-0.06	-0.08	0.04	0.05	0.07	0.03	0.01	0.08
15	1	0.01	0.03	0.04	0.04	0.01	0.03	-0.09	-0.06	-0.02
16	1	-0.05	-0.11	-0.16	0.02	0.05	0.08	0.13	0.05	0.13
17	1	-0.05	-0.11	-0.14	0.07	0.11	0.13	0.04	0.00	0.13
18	7	0.02	0.05	-0.02	-0.03	-0.03	0.02	-0.00	0.10	0.05
19	6	0.06	0.10	0.07	-0.08	-0.09	-0.08	-0.08	-0.01	-0.01
20	1	0.22	0.23	0.09	-0.22	-0.17	-0.10	-0.24	-0.17	-0.02
21	1	0.02	0.03	-0.07	-0.06	-0.05	0.00	-0.09	0.07	0.14
22	1	-0.03	0.08	0.25	0.02	-0.10	-0.22	0.04	-0.02	-0.19
23	6	-0.01	-0.01	-0.11	-0.01	0.02	0.10	0.04	0.06	-0.02
24	1	-0.01	0.01	-0.17	-0.02	0.01	0.15	0.05	0.04	-0.02
25	1	-0.00	0.02	-0.15	-0.02	-0.00	0.13	0.05	0.10	-0.07
26	1	-0.02	-0.09	-0.10	0.01	0.09	0.09	0.06	-0.00	-0.01
27	6	0.02	-0.12	0.07	0.02	-0.17	0.04	-0.03	-0.13	-0.06
28	6	-0.02	-0.01	0.15	-0.03	-0.10	0.11	-0.03	-0.15	-0.07
29	1	0.09	-0.22	0.06	0.09	-0.33	0.01	-0.04	-0.14	-0.07
30	1	-0.03	0.08	0.18	-0.03	0.05	0.17	-0.02	-0.14	-0.07
31	7	0.02	-0.07	-0.04	0.03	0.10	-0.01	-0.03	-0.07	-0.03
32	8	-0.09	-0.08	-0.10	-0.07	0.18	-0.04	-0.01	0.03	0.01
33	8	0.12	-0.02	-0.09	0.12	0.26	-0.03	-0.05	-0.14	-0.03
34	6	-0.02	0.02	0.12	-0.02	-0.10	0.06	-0.03	-0.13	-0.07
35	6	0.06	0.04	0.09	0.07	-0.05	0.05	0.00	-0.09	-0.05
36	6	-0.10	0.02	0.05	-0.11	-0.07	0.01	-0.04	-0.10	-0.05
37	6	0.05	0.04	-0.03	0.09	0.06	-0.01	0.02	0.01	0.01
38	1	0.12	0.06	0.16	0.14	-0.07	0.09	0.01	-0.13	-0.07
39	6	-0.11	0.01	-0.08	-0.10	0.03	-0.05	-0.02	0.01	0.00
40	1	-0.16	0.02	0.09	-0.18	-0.10	0.03	-0.07	-0.14	-0.08
41	6	-0.03	0.01	-0.12	0.01	0.10	-0.05	0.01	0.07	0.04
42	1	0.12	0.05	-0.06	0.18	0.11	-0.01	0.05	0.05	0.04
43	1	-0.17	-0.01	-0.15	-0.17	0.07	-0.08	-0.03	0.05	0.02
44	1	-0.04	-0.00	-0.23	0.02	0.18	-0.09	0.03	0.17	0.10

	13	14	15
	A	A	A
Frequencies --	133.5889	153.6819	186.2589
Red. masses --	5.0472	3.2942	4.5486
Frc consts --	0.0531	0.0458	0.0930
IR Inten --	4.7885	1.5224	5.1845

Atom	AN	X	Y	Z	X	Y	Z	X	Y	Z
1	6	0.00	-0.00	0.11	-0.00	0.00	-0.00	-0.03	-0.08	0.03
2	6	0.06	-0.09	0.04	0.00	-0.02	-0.01	-0.03	-0.00	0.06
3	6	0.08	-0.03	-0.04	-0.00	-0.02	-0.02	0.01	0.12	0.04
4	1	0.13	-0.01	-0.03	0.00	-0.02	-0.02	0.08	0.19	0.09
5	1	0.11	-0.16	0.05	-0.00	-0.04	-0.02	-0.03	-0.05	0.06
6	16	-0.06	0.10	0.11	-0.01	0.03	-0.00	0.16	0.07	0.08
7	6	0.01	0.03	0.13	-0.00	0.00	0.00	-0.08	-0.17	-0.05
8	6	0.02	-0.03	-0.00	-0.00	-0.00	-0.01	-0.00	-0.12	-0.04

9	6	-0.03	0.10	0.17	-0.00	0.01	0.01	-0.14	-0.13	-0.06
10	6	-0.02	-0.03	-0.15	-0.00	-0.00	-0.01	0.05	0.04	0.03
11	1	0.05	-0.08	-0.03	-0.00	-0.01	-0.01	0.04	-0.16	-0.04
12	6	-0.06	0.12	0.05	-0.00	0.01	0.01	-0.11	0.00	-0.01
13	1	-0.06	0.12	0.25	0.00	0.01	0.01	-0.19	-0.16	-0.05
14	6	-0.06	0.04	-0.12	-0.00	0.01	-0.00	0.00	0.11	0.06
15	1	-0.01	-0.09	-0.28	-0.01	-0.01	-0.02	0.13	0.11	0.08
16	1	-0.10	0.17	0.08	0.00	0.02	0.01	-0.16	0.05	-0.00
17	1	-0.10	0.02	-0.23	-0.00	0.01	-0.01	0.05	0.24	0.14
18	7	0.05	-0.03	-0.08	0.00	-0.01	-0.01	-0.05	0.04	-0.08
19	6	0.11	0.05	0.01	0.01	-0.00	0.00	-0.04	0.04	-0.02
20	1	0.26	0.16	0.02	0.07	0.07	0.00	0.03	0.09	-0.02
21	1	0.12	0.00	-0.09	-0.00	-0.04	-0.07	-0.07	0.02	-0.08
22	1	0.02	0.08	0.15	-0.02	-0.02	0.08	-0.07	0.03	0.05
23	6	0.03	-0.06	-0.13	0.01	0.00	0.02	0.03	-0.03	-0.20
24	1	0.02	-0.04	-0.15	0.01	-0.01	0.02	-0.04	0.06	0.01
25	1	0.02	-0.05	-0.16	0.01	-0.00	0.04	-0.10	-0.04	-0.47
26	1	0.04	-0.11	-0.12	0.00	0.03	0.01	0.33	-0.16	-0.22
27	6	-0.02	-0.12	-0.06	0.01	0.27	0.16	-0.00	0.03	0.02
28	6	-0.02	-0.12	-0.06	-0.02	-0.11	0.02	0.00	0.03	0.02
29	1	-0.02	-0.16	-0.08	0.05	0.61	0.29	-0.00	0.02	0.02
30	1	-0.01	-0.08	-0.04	-0.04	-0.47	-0.13	0.01	0.02	0.02
31	7	-0.01	0.00	-0.01	0.00	0.06	0.02	-0.00	-0.01	0.00
32	8	0.01	0.05	0.01	-0.07	0.05	-0.02	-0.01	-0.02	-0.00
33	8	-0.03	0.08	0.02	0.08	-0.13	-0.07	0.00	-0.05	-0.01
34	6	-0.01	-0.12	-0.06	-0.01	-0.09	0.00	0.00	0.02	0.01
35	6	0.01	-0.09	-0.05	0.04	-0.06	0.00	-0.00	0.01	0.01
36	6	-0.03	-0.08	-0.04	-0.05	-0.08	-0.02	0.01	0.01	0.01
37	6	0.02	-0.00	-0.00	0.05	0.03	0.01	-0.01	-0.00	0.00
38	1	0.02	-0.13	-0.07	0.07	-0.08	0.01	-0.01	0.02	0.01
39	6	-0.01	0.01	0.00	-0.04	-0.02	-0.04	0.00	0.00	0.01
40	1	-0.05	-0.11	-0.06	-0.08	-0.13	-0.05	0.01	0.02	0.01
41	6	0.02	0.05	0.03	0.01	0.05	-0.01	-0.00	-0.01	0.00
42	1	0.04	0.03	0.02	0.10	0.08	0.03	-0.01	-0.01	-0.00
43	1	-0.02	0.04	0.02	-0.08	-0.02	-0.07	0.01	-0.00	0.01
44	1	0.03	0.13	0.08	0.02	0.11	-0.01	-0.01	-0.02	-0.00

	16	17	18
	A	A	A
Frequencies --	189.9209	224.3183	243.5686
Red. masses --	1.4755	3.6699	4.7854
Frc consts --	0.0314	0.1088	0.1673
IR Inten --	0.3607	2.2987	1.0042

Atom	AN	X	Y	Z	X	Y	Z	X	Y	Z
1	6	0.01	0.03	0.01	-0.04	-0.02	-0.01	-0.03	0.05	0.00
2	6	-0.01	0.04	0.02	0.05	0.06	-0.05	-0.03	-0.10	-0.05
3	6	-0.03	-0.02	-0.02	0.06	0.05	-0.09	-0.02	-0.10	-0.14
4	1	-0.02	-0.03	-0.03	0.01	-0.01	-0.13	-0.02	-0.15	-0.20

5	1	0.00	0.08	0.03	0.08	-0.00	-0.04	0.02	-0.17	-0.04
6	16	0.04	-0.03	0.01	-0.05	-0.07	-0.01	0.19	-0.10	0.05
7	6	0.01	0.03	-0.01	-0.08	-0.06	0.01	-0.05	0.15	-0.00
8	6	-0.01	0.02	-0.00	-0.06	-0.04	0.02	-0.14	0.13	0.05
9	6	0.02	0.01	-0.01	-0.13	-0.03	0.00	-0.02	0.11	-0.01
10	6	-0.01	-0.00	0.01	-0.05	0.03	0.03	-0.17	0.02	0.07
11	1	-0.02	0.03	-0.00	-0.04	-0.05	0.02	-0.20	0.19	0.05
12	6	0.02	-0.01	0.00	-0.13	0.02	-0.00	-0.03	0.01	0.02
13	1	0.04	0.02	-0.02	-0.15	-0.04	0.03	0.02	0.12	-0.04
14	6	0.01	-0.01	0.01	-0.09	0.06	0.02	-0.13	-0.04	0.04
15	1	-0.03	-0.01	0.01	0.00	0.06	0.03	-0.23	-0.01	0.08
16	1	0.04	-0.02	0.00	-0.15	0.03	-0.01	0.03	-0.05	0.01
17	1	0.00	-0.03	0.02	-0.07	0.11	0.04	-0.15	-0.13	0.03
18	7	-0.07	-0.06	-0.08	0.14	0.05	0.01	0.02	-0.03	-0.06
19	6	-0.03	-0.01	-0.02	0.06	-0.10	-0.03	0.07	0.05	0.02
20	1	0.31	0.45	-0.02	0.11	0.07	-0.05	-0.01	-0.21	0.05
21	1	-0.16	-0.23	-0.48	-0.10	-0.17	-0.22	0.19	0.18	0.29
22	1	-0.20	-0.19	0.48	0.09	-0.29	0.14	0.07	0.25	-0.21
23	6	-0.03	-0.00	0.02	0.28	0.10	0.08	0.05	-0.01	-0.02
24	1	-0.02	-0.03	0.08	0.37	-0.07	0.00	-0.01	0.06	0.15
25	1	-0.02	-0.01	0.06	0.42	0.19	0.21	-0.05	-0.06	-0.14
26	1	-0.01	0.09	0.00	0.12	0.19	0.09	0.24	-0.00	-0.06
27	6	-0.01	-0.02	-0.00	-0.00	-0.01	0.00	0.02	0.01	-0.02
28	6	0.01	0.01	-0.00	0.00	0.01	0.01	-0.01	-0.03	-0.02
29	1	-0.01	-0.05	-0.02	-0.01	-0.03	-0.00	0.03	0.05	-0.00
30	1	0.02	0.04	0.02	0.01	0.03	0.02	-0.04	-0.07	-0.05
31	7	-0.01	0.01	0.01	-0.01	0.00	0.01	0.03	0.01	-0.01
32	8	-0.00	0.06	0.03	-0.01	0.01	0.01	0.06	0.01	0.00
33	8	-0.01	-0.00	0.01	-0.00	-0.00	0.00	0.01	0.02	0.00
34	6	0.01	-0.00	-0.01	0.01	0.01	-0.00	-0.02	-0.01	0.02
35	6	0.00	-0.01	-0.01	0.00	0.00	-0.00	-0.01	-0.00	0.02
36	6	0.01	-0.00	-0.01	0.01	0.00	-0.00	-0.03	0.00	0.02
37	6	0.00	-0.00	-0.00	0.00	-0.00	0.00	-0.01	0.00	0.00
38	1	-0.00	-0.01	-0.02	-0.00	0.01	-0.00	-0.01	-0.01	0.02
39	6	0.02	0.00	0.01	0.01	-0.00	0.00	-0.04	0.00	-0.00
40	1	0.02	-0.00	-0.01	0.02	0.01	-0.00	-0.04	0.00	0.02
41	6	0.01	0.00	0.01	0.01	-0.00	0.00	-0.03	-0.00	-0.01
42	1	-0.00	-0.01	0.00	-0.00	-0.00	0.00	0.00	0.01	-0.00
43	1	0.02	0.00	0.01	0.02	-0.00	0.01	-0.04	0.00	-0.01
44	1	0.01	0.01	0.02	0.01	-0.00	0.01	-0.03	-0.01	-0.03

	19	20	21
	A	A	A
Frequencies --	257.4536	283.6159	290.5859
Red. masses --	6.7447	1.1497	3.6327
Frc consts --	0.2634	0.0545	0.1807
IR Inten --	0.8930	4.5850	3.4325

Atom AN X Y Z X Y Z X Y Z

1	6	0.00	-0.00	0.00	-0.01	-0.01	-0.01	0.00	0.01	0.00
2	6	0.01	0.04	0.01	0.00	0.03	-0.01	0.01	0.01	-0.00
3	6	0.00	0.02	0.02	-0.00	0.00	-0.01	0.00	-0.01	-0.01
4	1	0.00	0.03	0.03	-0.02	-0.01	-0.02	-0.01	-0.02	-0.01
5	1	0.01	0.06	0.02	0.01	0.05	-0.00	0.01	0.02	0.00
6	16	-0.04	0.02	-0.01	-0.03	-0.01	-0.02	-0.01	-0.01	-0.00
7	6	0.00	-0.04	-0.00	0.01	-0.01	0.02	0.00	0.00	0.00
8	6	0.02	-0.03	-0.02	0.02	0.00	0.02	-0.00	-0.00	-0.00
9	6	-0.01	-0.03	-0.01	0.01	0.01	0.03	-0.00	-0.00	-0.00
10	6	0.03	-0.00	-0.01	0.02	0.00	-0.00	-0.00	-0.00	-0.00
11	1	0.03	-0.05	-0.02	0.03	0.00	0.03	-0.00	-0.00	-0.00
12	6	-0.00	-0.00	-0.01	0.00	0.00	0.01	-0.00	0.00	-0.00
13	1	-0.01	-0.03	-0.00	0.00	0.01	0.04	-0.00	-0.00	-0.00
14	6	0.02	0.02	-0.00	-0.00	-0.01	-0.02	-0.00	0.00	0.00
15	1	0.05	0.01	-0.01	0.02	-0.00	-0.01	-0.00	-0.00	-0.01
16	1	-0.02	0.01	-0.00	-0.01	0.01	0.01	-0.00	0.00	-0.00
17	1	0.03	0.04	0.01	-0.01	-0.02	-0.05	0.00	0.01	0.01
18	7	-0.01	-0.02	-0.02	0.00	-0.01	0.00	0.00	-0.02	-0.01
19	6	-0.02	-0.02	-0.02	-0.00	-0.01	-0.01	0.02	0.00	-0.00
20	1	0.08	0.11	-0.02	-0.00	0.01	-0.01	0.01	-0.01	-0.00
21	1	-0.06	-0.09	-0.15	-0.01	-0.02	-0.02	0.04	0.01	0.02
22	1	-0.05	-0.09	0.13	-0.00	-0.02	-0.00	0.01	0.03	-0.03
23	6	0.01	0.01	0.03	0.03	0.02	0.04	-0.00	-0.00	0.02
24	1	0.03	-0.04	0.01	-0.16	0.28	0.52	-0.05	0.06	0.12
25	1	0.05	0.02	0.08	-0.31	-0.18	-0.34	-0.08	-0.06	-0.05
26	1	-0.04	0.06	0.02	0.59	-0.04	-0.04	0.11	-0.00	0.00
27	6	0.14	0.05	-0.14	-0.00	-0.00	-0.00	0.05	0.04	0.06
28	6	-0.03	-0.10	-0.09	-0.00	-0.02	-0.01	0.02	0.22	0.14
29	1	0.17	0.16	-0.09	-0.00	0.02	0.00	0.06	-0.23	-0.02
30	1	-0.19	-0.22	-0.24	-0.00	-0.03	-0.02	0.05	0.49	0.26
31	7	0.20	0.01	-0.04	-0.00	-0.00	-0.00	0.08	0.01	0.03
32	8	0.40	-0.02	0.06	-0.00	0.00	-0.00	0.06	-0.07	0.00
33	8	0.03	-0.03	0.06	-0.01	0.00	0.00	0.11	-0.02	0.00
34	6	-0.09	-0.03	0.08	0.00	0.00	0.00	-0.03	-0.02	-0.05
35	6	-0.09	0.01	0.10	0.01	0.01	0.01	-0.09	-0.11	-0.12
36	6	-0.18	0.04	0.08	0.00	0.01	0.01	-0.07	-0.11	-0.14
37	6	-0.09	0.04	0.02	0.01	0.00	0.00	-0.08	-0.03	-0.04
38	1	-0.07	0.02	0.12	0.01	0.01	0.01	-0.12	-0.15	-0.18
39	6	-0.20	0.03	-0.03	0.00	0.00	0.00	-0.05	-0.00	-0.03
40	1	-0.24	0.05	0.13	0.01	0.01	0.01	-0.08	-0.17	-0.19
41	6	-0.18	0.00	-0.08	0.00	-0.01	-0.01	-0.05	0.14	0.12
42	1	-0.02	0.06	0.00	0.00	0.00	0.00	-0.08	-0.03	-0.04
43	1	-0.23	0.03	-0.06	0.00	-0.00	0.00	-0.03	0.01	0.00
44	1	-0.19	-0.03	-0.17	0.00	-0.02	-0.02	-0.01	0.33	0.29

22 23 24

A A A

Frequencies -- 312.6674 315.9624 350.3618

Red. masses -- 3.2951 2.9494 4.7744
Frc consts -- 0.1898 0.1735 0.3453
IR Inten -- 16.9543 4.8210 25.6512

Atom	AN	X	Y	Z	X	Y	Z	X	Y	Z
1	6	0.03	0.07	0.06	0.03	0.02	0.01	-0.03	0.02	0.10
2	6	0.09	0.18	0.06	0.03	-0.06	0.02	-0.07	-0.19	0.04
3	6	0.03	-0.07	-0.06	0.07	-0.03	0.12	-0.04	0.00	-0.07
4	1	-0.03	-0.13	-0.10	0.05	-0.02	0.14	0.06	0.00	-0.10
5	1	0.12	0.26	0.10	-0.01	-0.06	0.00	-0.04	-0.37	0.02
6	16	-0.05	0.01	0.05	-0.03	0.01	0.01	-0.11	-0.03	0.15
7	6	-0.02	-0.02	-0.03	-0.01	0.06	-0.06	0.02	0.10	-0.05
8	6	-0.05	-0.06	-0.10	-0.09	0.02	-0.07	-0.02	0.04	-0.15
9	6	-0.04	-0.06	-0.10	-0.02	0.02	-0.11	0.09	-0.01	-0.15
10	6	-0.01	0.01	-0.02	-0.08	0.01	0.01	0.01	-0.01	-0.06
11	1	-0.07	-0.10	-0.15	-0.16	0.03	-0.11	-0.06	0.01	-0.21
12	6	-0.02	-0.02	-0.05	-0.00	-0.00	-0.03	0.13	-0.06	-0.07
13	1	-0.03	-0.07	-0.13	-0.00	0.01	-0.15	0.14	-0.03	-0.25
14	6	0.03	0.07	0.08	-0.02	0.03	0.08	0.14	0.01	0.06
15	1	0.00	0.02	0.01	-0.12	0.00	0.03	-0.08	-0.04	-0.03
16	1	-0.03	-0.03	-0.06	0.04	-0.04	-0.04	0.15	-0.10	-0.09
17	1	0.08	0.16	0.21	0.01	0.05	0.18	0.18	0.04	0.20
18	7	-0.01	-0.16	-0.12	0.11	0.02	0.18	-0.03	0.09	-0.04
19	6	0.13	0.04	0.02	0.10	0.01	-0.14	-0.10	-0.01	0.05
20	1	0.14	-0.18	0.06	-0.00	0.36	-0.24	-0.06	-0.10	0.08
21	1	0.32	0.15	0.28	0.15	-0.16	-0.43	-0.22	0.03	0.09
22	1	0.04	0.33	-0.15	0.16	-0.15	-0.04	-0.07	-0.08	0.07
23	6	-0.05	-0.02	0.09	-0.05	-0.11	-0.01	0.15	0.09	-0.05
24	1	-0.08	0.01	0.20	-0.13	0.08	-0.04	0.15	0.03	0.10
25	1	-0.10	-0.14	0.21	-0.17	-0.16	-0.18	0.14	0.15	-0.19
26	1	-0.02	0.17	0.06	0.04	-0.33	0.01	0.31	0.07	-0.08
27	6	-0.01	-0.00	0.00	0.00	-0.00	-0.00	0.00	-0.01	0.01
28	6	-0.00	-0.01	-0.00	0.00	-0.00	-0.00	0.00	-0.01	0.00
29	1	-0.01	0.01	0.00	0.00	0.00	-0.00	0.00	-0.01	0.01
30	1	-0.00	-0.02	-0.01	-0.00	-0.01	-0.00	0.02	-0.02	0.00
31	7	-0.01	-0.00	-0.01	0.00	0.00	0.00	0.01	-0.00	0.01
32	8	-0.02	-0.00	-0.01	0.00	0.00	0.00	-0.00	0.00	0.01
33	8	-0.01	0.01	-0.00	-0.00	0.00	0.00	0.02	0.00	0.00
34	6	0.00	0.00	0.01	0.00	0.00	0.00	0.00	0.01	-0.01
35	6	0.01	0.01	0.01	0.00	0.00	0.01	-0.01	0.01	-0.00
36	6	0.01	0.01	0.01	0.00	0.00	0.00	0.00	0.01	-0.01
37	6	0.01	0.00	0.00	0.00	0.00	0.00	-0.01	0.00	0.00
38	1	0.02	0.02	0.02	0.00	0.01	0.01	-0.02	0.02	-0.01
39	6	0.01	0.00	0.00	-0.00	0.00	0.00	0.00	0.00	-0.00
40	1	0.01	0.01	0.02	0.00	0.01	0.01	0.01	0.01	-0.01
41	6	0.01	-0.01	-0.01	-0.00	-0.00	-0.00	-0.01	-0.00	0.00
42	1	0.01	0.00	0.00	0.00	0.00	0.00	-0.02	0.00	0.00
43	1	0.00	0.00	0.00	-0.00	-0.00	-0.00	0.01	-0.00	0.00

44	1	0.01	-0.02	-0.02	-0.00	-0.01	-0.01	-0.01	-0.01	0.00

TS1A

Zero-point correction= 0.357544 (Hartree/Particle)
Thermal correction to Energy= 0.379139
Thermal correction to Enthalpy= 0.380083
Thermal correction to Gibbs Free Energy= 0.305984
Sum of electronic and zero-point Energies= -1393.589458
Sum of electronic and thermal Energies= -1393.567864
Sum of electronic and thermal Enthalpies= -1393.566920
Sum of electronic and thermal Free Energies= -1393.641018

Center Number	Atomic Number	Atomic Type	Coordinates (Angstroms) X	Y	Z
1	6	0	-0.000916	-1.263476	-0.144596
2	6	0	0.913164	-1.537071	0.828506
3	6	0	2.314640	-1.225150	0.870982
4	1	0	2.706170	-0.846204	1.811235
5	1	0	0.565996	-1.969179	1.763767
6	16	0	0.404154	-0.240120	-1.492595
7	6	0	-1.418787	-1.692772	0.026856
8	6	0	-2.210997	-1.957727	-1.093567
9	6	0	-1.997438	-1.830874	1.293679
10	6	0	-3.530591	-2.365438	-0.953803
11	1	0	-1.787451	-1.841993	-2.084287
12	6	0	-3.320802	-2.225376	1.433867
13	1	0	-1.430051	-1.592099	2.186243
14	6	0	-4.092993	-2.498391	0.310499
15	1	0	-4.123185	-2.573175	-1.838261
16	1	0	-3.752140	-2.308300	2.425698
17	1	0	-5.127712	-2.804813	0.419745
18	7	0	3.219044	-1.454422	-0.032567
19	6	0	4.618588	-1.082935	0.193939
20	1	0	4.973300	-0.531630	-0.674763
21	1	0	4.693801	-0.436068	1.062950
22	1	0	5.210190	-1.990788	0.323935
23	6	0	2.972557	-2.155599	-1.284122
24	1	0	3.810616	-2.831104	-1.456543
25	1	0	2.047898	-2.722714	-1.220713
26	1	0	2.902592	-1.437678	-2.100884
27	6	0	1.420464	1.542279	0.703330
28	6	0	0.563080	1.632633	-0.414791
29	1	0	1.093442	1.482283	1.728039
30	1	0	1.013044	2.124697	-1.271566
31	7	0	2.778883	1.605085	0.550157
32	8	0	3.282357	1.652602	-0.588300
33	8	0	3.498431	1.572035	1.576921

34	6	0	-0.860191	2.004625	-0.177854
35	6	0	-1.526881	2.792841	-1.117659
36	6	0	-1.558137	1.575933	0.952538
37	6	0	-2.857386	3.144990	-0.933593
38	1	0	-0.998505	3.126913	-2.005549
39	6	0	-2.891342	1.918478	1.133360
40	1	0	-1.067868	0.951154	1.690735
41	6	0	-3.546101	2.705237	0.192052
42	1	0	-3.358688	3.760284	-1.673226
43	1	0	-3.421502	1.564768	2.011278
44	1	0	-4.587792	2.972578	0.333841

IA

Zero-point correction= 0.358333 (Hartree/Particle)
Thermal correction to Energy= 0.380361
Thermal correction to Enthalpy= 0.381305
Thermal correction to Gibbs Free Energy= 0.306416
Sum of electronic and zero-point Energies= -1393.589272
Sum of electronic and thermal Energies= -1393.567245
Sum of electronic and thermal Enthalpies= -1393.566300
Sum of electronic and thermal Free Energies= -1393.641190

Center Number	Atomic Number	Atomic Type	Coordinates (Angstroms) X	Y	Z
1	6	0	0.032017	-1.253333	0.108381
2	6	0	-0.877919	-1.544480	-0.848148
3	6	0	-2.281910	-1.189528	-0.891489
4	1	0	-2.662740	-0.761135	-1.814406
5	1	0	-0.547974	-2.031522	-1.763010
6	16	0	-0.407333	-0.201060	1.452644
7	6	0	1.448079	-1.690540	-0.022280
8	6	0	2.221303	-1.920469	1.118690
9	6	0	2.042299	-1.875007	-1.275887
10	6	0	3.539517	-2.342826	1.011611
11	1	0	1.787068	-1.766400	2.099574
12	6	0	3.363745	-2.284957	-1.382920
13	1	0	1.487620	-1.662064	-2.183081
14	6	0	4.117439	-2.524956	-0.239228
15	1	0	4.118514	-2.522812	1.910931
16	1	0	3.808853	-2.405552	-2.364656
17	1	0	5.151049	-2.842715	-0.322983
18	7	0	-3.186679	-1.465033	-0.006272
19	6	0	-4.590843	-1.100212	-0.224329
20	1	0	-4.964278	-0.621247	0.678041
21	1	0	-4.663524	-0.390399	-1.042881
22	1	0	-5.161337	-2.008326	-0.427334
23	6	0	-2.939469	-2.207288	1.223152
24	1	0	-3.763253	-2.907793	1.359432
25	1	0	-2.001155	-2.750358	1.155044
26	1	0	-2.901804	-1.514658	2.063184
27	6	0	-1.449415	1.475349	-0.662675
28	6	0	-0.559807	1.522142	0.478621
29	1	0	-1.115711	1.494560	-1.687052
30	1	0	-1.006490	2.083697	1.298431
31	7	0	-2.786184	1.567438	-0.499131
32	8	0	-3.285754	1.574975	0.652573
33	8	0	-3.528910	1.585673	-1.522181

34	6	0	0.839866	1.984392	0.188996
35	6	0	1.482621	2.834154	1.089636
36	6	0	1.533482	1.567227	-0.947820
37	6	0	2.787177	3.255043	0.863781
38	1	0	0.956948	3.164830	1.980552
39	6	0	2.840921	1.979098	-1.171529
40	1	0	1.059686	0.901271	-1.660610
41	6	0	3.472944	2.825318	-0.267130
42	1	0	3.269324	3.918202	1.574315
43	1	0	3.367862	1.634791	-2.055203
44	1	0	4.493739	3.147794	-0.442507

TS2A

Zero-point correction= 0.358180 (Hartree/Particle)
Thermal correction to Energy= 0.379548
Thermal correction to Enthalpy= 0.380492
Thermal correction to Gibbs Free Energy= 0.306396
Sum of electronic and zero-point Energies= -1393.588481
Sum of electronic and thermal Energies= -1393.567113
Sum of electronic and thermal Enthalpies= -1393.566169
Sum of electronic and thermal Free Energies= -1393.640265

Center Number	Atomic Number	Atomic Type	Coordinates (Angstroms) X	Y	Z
1	6	0	0.265954	-1.203885	0.063740
2	6	0	-0.589737	-1.417066	-0.952801
3	6	0	-2.014337	-1.103396	-1.021903
4	1	0	-2.381336	-0.727906	-1.970615
5	1	0	-0.202146	-1.815442	-1.888356
6	16	0	-0.265728	-0.304889	1.489449
7	6	0	1.694351	-1.601164	-0.028040
8	6	0	2.369582	-2.066399	1.103261
9	6	0	2.390884	-1.523202	-1.237723
10	6	0	3.697665	-2.463119	1.023306
11	1	0	1.851581	-2.123750	2.054585
12	6	0	3.722423	-1.909467	-1.314820
13	1	0	1.902595	-1.130488	-2.122856
14	6	0	4.379793	-2.384939	-0.185821
15	1	0	4.201983	-2.829853	1.910793
16	1	0	4.249970	-1.828207	-2.259075
17	1	0	5.420381	-2.684839	-0.245933
18	7	0	-2.943158	-1.634394	-0.256872
19	6	0	-4.362010	-1.490226	-0.586725
20	1	0	-4.881752	-1.023146	0.247797
21	1	0	-4.479708	-0.855632	-1.459567
22	1	0	-4.782412	-2.480609	-0.775424
23	6	0	-2.681785	-2.447587	0.921953
24	1	0	-3.388396	-3.278536	0.920209
25	1	0	-1.669652	-2.840640	0.896999
26	1	0	-2.822175	-1.854513	1.825107
27	6	0	-1.688339	1.209828	-0.478240
28	6	0	-0.721567	1.350389	0.620883
29	1	0	-1.447881	1.545397	-1.475266
30	1	0	-1.189692	1.853327	1.468200
31	7	0	-3.025023	1.306018	-0.193979
32	8	0	-3.421609	1.096327	0.969462
33	8	0	-3.828684	1.499567	-1.131835

34	6	0	0.560752	2.041485	0.234653
35	6	0	1.157179	2.931921	1.127766
36	6	0	1.191635	1.797200	-0.985995
37	6	0	2.355904	3.559443	0.813429
38	1	0	0.680161	3.131110	2.082878
39	6	0	2.394422	2.417811	-1.299898
40	1	0	0.748579	1.111823	-1.699863
41	6	0	2.981562	3.301325	-0.401634
42	1	0	2.802396	4.251095	1.520027
43	1	0	2.873266	2.209132	-2.250842
44	1	0	3.919516	3.787670	-0.647394

4

Zero-point correction= 0.360616 (Hartree/Particle)
Thermal correction to Energy= 0.381946
Thermal correction to Enthalpy= 0.382891
Thermal correction to Gibbs Free Energy= 0.309074
Sum of electronic and zero-point Energies= -1393.631505
Sum of electronic and thermal Energies= -1393.610174
Sum of electronic and thermal Enthalpies= -1393.609230
Sum of electronic and thermal Free Energies= -1393.683046

Center Number	Atomic Number	Atomic Type	Coordinates (Angstroms) X	Y	Z
1	6	0	1.764434	-0.019682	-0.172490
2	6	0	1.456394	-1.307087	-0.344902
3	6	0	0.096533	-1.938504	-0.475145
4	1	0	0.103102	-2.508576	-1.410469
5	1	0	2.269207	-2.027303	-0.363528
6	16	0	0.570167	1.282973	-0.031927
7	6	0	3.173483	0.447863	-0.110188
8	6	0	3.601296	1.327207	0.888366
9	6	0	4.099100	-0.005039	-1.053867
10	6	0	4.929974	1.727249	0.951777
11	1	0	2.895958	1.684907	1.630728
12	6	0	5.427085	0.397332	-0.989417
13	1	0	3.769884	-0.665839	-1.848819
14	6	0	5.846517	1.263877	0.014042
15	1	0	5.250200	2.401267	1.738992
16	1	0	6.133607	0.039480	-1.730625
17	1	0	6.882813	1.580537	0.062588
18	7	0	-0.082008	-2.919871	0.574219
19	6	0	-0.933703	-4.062604	0.305961
20	1	0	-1.999754	-3.888541	0.511690
21	1	0	-0.830778	-4.368745	-0.736916
22	1	0	-0.613938	-4.900625	0.932702
23	6	0	-0.123954	-2.469640	1.949776
24	1	0	0.137460	-3.303566	2.607492
25	1	0	0.613810	-1.680324	2.109823
26	1	0	-1.112257	-2.098895	2.260144
27	6	0	-1.001226	-0.848017	-0.687207
28	6	0	-0.960196	0.328552	0.286891
29	1	0	-0.923368	-0.490356	-1.713647
30	1	0	-0.911904	-0.037005	1.309910
31	7	0	-2.336263	-1.539868	-0.637095
32	8	0	-2.950279	-1.541628	0.409382
33	8	0	-2.696209	-2.100494	-1.651615

34	6	0	-2.121673	1.289509	0.155094
35	6	0	-2.832140	1.667841	1.292171
36	6	0	-2.491951	1.816639	-1.082419
37	6	0	-3.899234	2.552489	1.196649
38	1	0	-2.554080	1.259327	2.258612
39	6	0	-3.562342	2.697034	-1.180014
40	1	0	-1.942913	1.547924	-1.979425
41	6	0	-4.268242	3.067601	-0.040772
42	1	0	-4.445131	2.835962	2.089976
43	1	0	-3.842613	3.096474	-2.148628
44	1	0	-5.103115	3.755748	-0.117609

MCB

Zero-point correction= 0.357004 (Hartree/Particle)
Thermal correction to Energy= 0.380403
Thermal correction to Enthalpy= 0.381347
Thermal correction to Gibbs Free Energy= 0.302049
Sum of electronic and zero-point Energies= -1393.627197
Sum of electronic and thermal Energies= -1393.603798
Sum of electronic and thermal Enthalpies= -1393.602854
Sum of electronic and thermal Free Energies= -1393.682152

Center Number	Atomic Number	Atomic Type	Coordinates (Angstroms) X	Y	Z
1	6	0	-0.284807	-1.668744	-0.460592
2	6	0	-1.397129	-1.428937	0.370734
3	6	0	-2.658808	-1.164646	-0.135074
4	1	0	-2.785364	-1.147988	-1.211911
5	1	0	-1.240339	-1.420748	1.442291
6	16	0	-0.350530	-1.845587	-2.142652
7	6	0	1.041621	-1.730708	0.219703
8	6	0	2.043085	-2.597096	-0.227027
9	6	0	1.325597	-0.882343	1.295871
10	6	0	3.287058	-2.619988	0.387173
11	1	0	1.835769	-3.257648	-1.060667
12	6	0	2.577712	-0.887111	1.894216
13	1	0	0.579085	-0.173612	1.636415
14	6	0	3.561719	-1.759192	1.444932
15	1	0	4.047680	-3.308461	0.034312
16	1	0	2.788003	-0.196788	2.703844
17	1	0	4.540430	-1.765166	1.913119
18	7	0	-3.741104	-0.897610	0.572004
19	6	0	-4.980419	-0.485622	-0.070481
20	1	0	-5.181681	0.562345	0.160810
21	1	0	-4.889157	-0.595097	-1.149000
22	1	0	-5.807114	-1.103995	0.284161
23	6	0	-3.698532	-0.853994	2.025890
24	1	0	-3.046998	-0.045149	2.366220
25	1	0	-4.702470	-0.675528	2.404580
26	1	0	-3.335059	-1.802282	2.426419
27	6	0	-0.987952	2.194455	0.140076
28	6	0	-0.067480	1.780225	-0.731524
29	6	0	1.376598	1.836957	-0.528525
30	6	0	2.188887	1.034076	-1.335635
31	6	0	1.969863	2.617206	0.471796
32	6	0	3.559861	0.987588	-1.130155
33	1	0	1.732116	0.412752	-2.099077

34	6	0	3.340403	2.576393	0.668577
35	1	0	1.363139	3.267350	1.093104
36	6	0	4.137026	1.756014	-0.126790
37	1	0	4.175652	0.344177	-1.748233
38	1	0	3.791759	3.185518	1.444006
39	1	0	5.208772	1.719810	0.036461
40	7	0	-2.391981	2.030260	-0.148027
41	8	0	-3.164090	2.265061	0.771451
42	8	0	-2.748091	1.665514	-1.255693
43	1	0	-0.822460	2.612611	1.121210
44	1	0	-0.420066	1.316928	-1.648517

Harmonic frequencies (cm**-1), IR intensities (KM/Mole), Raman scattering activities (A**4/AMU), depolarization ratios for plane and unpolarized incident light, reduced masses (AMU), force constants (mDyne/A), and normal coordinates:

		1			2			3	
		A			A			A	
Frequencies --		17.7135			33.0568			49.5862	
Red. masses --		6.1772			6.3090			5.8274	
Frc consts --		0.0011			0.0041			0.0084	
IR Inten --		0.6080			1.6245			2.7585	
Atom	AN	X	Y	Z	X	Y	Z	X	Y	Z
1	6	0.07	-0.01	-0.00	0.06	-0.03	-0.00	0.01	0.11	0.03
2	6	0.09	0.03	0.01	0.03	0.00	-0.04	0.01	0.05	0.04
3	6	0.09	0.04	0.02	0.06	0.03	-0.08	-0.00	0.01	0.04
4	1	0.06	-0.01	0.03	0.10	0.07	-0.09	-0.01	0.02	0.04
5	1	0.11	0.05	0.01	0.00	0.01	-0.04	0.01	0.02	0.04
6	16	0.05	-0.03	0.00	0.10	-0.09	0.00	0.02	0.19	0.02
7	6	0.08	-0.05	-0.01	0.04	-0.01	0.03	0.01	0.10	0.03
8	6	0.04	-0.09	-0.01	0.04	-0.03	0.10	0.01	0.10	0.01
9	6	0.12	-0.05	-0.02	0.01	0.04	0.00	0.01	0.08	0.04
10	6	0.05	-0.13	-0.02	0.03	-0.01	0.14	0.01	0.09	0.02
11	1	0.02	-0.09	-0.01	0.06	-0.07	0.13	0.01	0.12	0.00
12	6	0.12	-0.10	-0.03	-0.00	0.07	0.03	0.00	0.07	0.04
13	1	0.15	-0.02	-0.03	0.01	0.06	-0.05	0.00	0.07	0.05
14	6	0.08	-0.14	-0.02	0.01	0.04	0.10	0.00	0.07	0.04
15	1	0.02	-0.17	-0.01	0.03	-0.03	0.19	0.01	0.09	0.02
16	1	0.15	-0.10	-0.03	-0.02	0.11	-0.00	0.01	0.06	0.05
17	1	0.08	-0.18	-0.02	-0.00	0.06	0.12	0.00	0.06	0.04
18	7	0.11	0.09	0.04	0.03	0.00	-0.12	-0.01	-0.03	0.05
19	6	0.08	0.01	0.05	0.06	0.05	-0.14	-0.02	-0.06	0.05
20	1	0.04	0.01	0.00	0.03	0.02	-0.06	-0.02	-0.05	0.01
21	1	0.05	-0.05	0.05	0.11	0.14	-0.15	-0.04	-0.10	0.05
22	1	0.11	-0.01	0.10	0.04	0.01	-0.24	-0.01	-0.05	0.08
23	6	0.15	0.16	0.04	-0.04	-0.08	-0.11	-0.01	-0.03	0.05
24	1	0.23	0.13	-0.02	-0.09	-0.07	-0.03	-0.02	-0.01	0.04

25	1	0.17	0.28	0.05	-0.06	-0.15	-0.15	-0.01	-0.05	0.05
26	1	0.08	0.15	0.08	-0.02	-0.09	-0.15	0.02	-0.02	0.05
27	6	-0.12	0.04	-0.04	-0.02	0.04	0.09	0.00	-0.09	-0.04
28	6	-0.10	0.02	-0.01	-0.07	0.05	0.03	0.00	0.02	-0.10
29	6	-0.10	0.00	0.03	-0.06	0.00	-0.03	-0.00	-0.03	-0.07
30	6	-0.09	0.04	-0.00	-0.12	0.02	-0.10	-0.01	0.04	-0.15
31	6	-0.12	-0.06	0.08	0.00	-0.06	-0.02	-0.00	-0.19	0.05
32	6	-0.09	0.03	0.02	-0.11	-0.04	-0.15	-0.02	-0.05	-0.10
33	1	-0.07	0.09	-0.05	-0.16	0.07	-0.11	-0.01	0.16	-0.24
34	6	-0.12	-0.08	0.11	0.01	-0.11	-0.07	-0.01	-0.27	0.10
35	1	-0.12	-0.09	0.11	0.05	-0.07	0.04	0.01	-0.24	0.12
36	6	-0.11	-0.03	0.07	-0.05	-0.10	-0.14	-0.02	-0.21	0.02
37	1	-0.08	0.06	-0.01	-0.15	-0.03	-0.21	-0.02	0.01	-0.17
38	1	-0.13	-0.12	0.15	0.06	-0.17	-0.06	-0.01	-0.40	0.19
39	1	-0.11	-0.05	0.09	-0.04	-0.15	-0.18	-0.03	-0.28	0.06
40	7	-0.11	0.08	-0.08	-0.04	0.09	0.14	-0.00	-0.06	-0.06
41	8	-0.13	0.14	-0.12	0.01	0.03	0.19	0.00	-0.10	-0.05
42	8	-0.08	0.04	-0.08	-0.09	0.18	0.12	-0.00	-0.01	-0.07
43	1	-0.14	0.05	-0.04	0.03	-0.01	0.10	0.00	-0.21	0.01
44	1	-0.08	0.03	-0.02	-0.12	0.10	0.03	0.00	0.14	-0.16

4 5 6

A A A

Frequencies -- 52.9831 60.3697 73.9184

Red. masses -- 5.7151 4.4663 3.1933

Frc consts -- 0.0095 0.0096 0.0103

IR Inten -- 2.9508 5.0907 0.9116

Atom	AN	X	Y	Z	X	Y	Z	X	Y	Z
1	6	-0.01	-0.07	-0.01	-0.04	-0.05	0.01	0.00	0.01	-0.03
2	6	-0.01	-0.05	-0.01	-0.03	0.00	0.01	-0.00	0.02	-0.04
3	6	-0.00	-0.02	-0.01	-0.03	-0.01	0.00	-0.01	-0.01	-0.04
4	1	-0.01	-0.03	-0.01	-0.03	-0.06	0.00	-0.04	-0.12	-0.04
5	1	-0.01	-0.03	-0.01	-0.03	0.05	0.01	-0.00	0.04	-0.04
6	16	-0.04	-0.16	0.00	-0.04	-0.12	0.02	0.02	0.01	-0.04
7	6	0.00	0.02	-0.03	-0.03	-0.03	0.00	-0.01	0.01	-0.01
8	6	0.09	0.17	-0.11	-0.06	-0.08	0.05	0.01	0.03	-0.01
9	6	-0.06	-0.03	0.03	0.00	0.06	-0.07	-0.03	-0.01	0.01
10	6	0.12	0.27	-0.16	-0.04	-0.04	0.02	-0.00	0.03	0.02
11	1	0.15	0.20	-0.16	-0.09	-0.16	0.12	0.04	0.05	-0.03
12	6	-0.05	0.07	-0.00	0.02	0.11	-0.12	-0.05	-0.02	0.04
13	1	-0.12	-0.12	0.09	0.01	0.08	-0.11	-0.04	-0.02	0.01
14	6	0.04	0.22	-0.10	0.00	0.06	-0.07	-0.03	0.00	0.04
15	1	0.19	0.39	-0.24	-0.06	-0.09	0.07	0.01	0.04	0.02
16	1	-0.11	0.03	0.04	0.06	0.19	-0.20	-0.07	-0.04	0.06
17	1	0.06	0.30	-0.13	0.02	0.10	-0.10	-0.04	-0.01	0.07
18	7	0.01	0.04	-0.01	-0.03	0.03	-0.01	0.03	0.09	-0.03
19	6	0.02	0.06	-0.01	-0.03	0.00	-0.03	-0.03	-0.07	-0.03
20	1	0.03	0.06	-0.00	-0.03	0.01	-0.07	-0.08	-0.05	-0.15

21	1	0.01	0.07	-0.01	-0.03	-0.04	-0.03	-0.07	-0.21	-0.02
22	1	0.00	0.08	-0.01	-0.03	0.02	-0.01	0.03	-0.07	0.09
23	6	0.02	0.06	-0.01	-0.03	0.09	-0.01	0.10	0.28	-0.04
24	1	0.06	0.04	-0.03	-0.01	0.09	-0.05	0.33	0.15	-0.18
25	1	0.03	0.12	-0.01	-0.03	0.14	-0.02	0.16	0.61	-0.02
26	1	-0.02	0.05	0.01	-0.06	0.10	0.03	-0.14	0.24	0.09
27	6	-0.01	0.01	0.02	0.02	-0.03	0.04	0.00	-0.08	0.05
28	6	-0.00	-0.05	0.05	0.04	0.00	0.05	-0.01	-0.03	0.01
29	6	-0.00	-0.07	0.06	0.04	0.02	0.05	-0.01	-0.01	-0.01
30	6	-0.02	-0.08	0.06	0.05	0.17	-0.08	-0.01	-0.03	0.00
31	6	0.01	-0.08	0.07	0.02	-0.10	0.15	-0.01	0.01	-0.02
32	6	-0.02	-0.10	0.06	0.06	0.20	-0.12	-0.01	-0.02	0.00
33	1	-0.02	-0.07	0.06	0.06	0.26	-0.15	-0.01	-0.04	0.02
34	6	0.00	-0.10	0.07	0.03	-0.07	0.12	-0.00	0.02	-0.03
35	1	0.01	-0.08	0.07	0.01	-0.22	0.26	-0.01	0.02	-0.04
36	6	-0.01	-0.11	0.06	0.05	0.09	-0.02	-0.00	0.00	-0.02
37	1	-0.03	-0.11	0.07	0.08	0.33	-0.23	-0.01	-0.03	0.01
38	1	0.01	-0.11	0.07	0.02	-0.17	0.21	-0.00	0.04	-0.05
39	1	-0.01	-0.13	0.07	0.05	0.11	-0.05	-0.00	0.01	-0.02
40	7	-0.01	0.04	-0.00	0.03	-0.04	0.00	-0.00	-0.08	0.06
41	8	-0.02	0.11	-0.03	-0.00	-0.04	-0.02	0.01	-0.15	0.09
42	8	0.01	0.02	0.00	0.06	-0.05	-0.00	-0.02	-0.01	0.04
43	1	-0.01	0.03	0.01	-0.01	-0.04	0.05	0.01	-0.13	0.07
44	1	-0.00	-0.06	0.06	0.05	0.02	0.03	-0.02	0.01	-0.01

	7	8	9
	A	A	A
Frequencies --	80.6676	83.3481	90.0381
Red. masses --	4.8835	5.3708	3.5768
Frc consts --	0.0187	0.0220	0.0171
IR Inten --	1.9269	1.0297	0.7277

Atom	AN	X	Y	Z	X	Y	Z	X	Y	Z
1	6	-0.00	-0.03	-0.12	0.00	-0.00	0.04	-0.00	-0.06	-0.01
2	6	-0.00	-0.08	-0.11	-0.02	-0.05	0.03	-0.01	-0.10	-0.01
3	6	-0.03	-0.08	-0.04	-0.01	-0.04	0.00	0.01	0.04	0.00
4	1	-0.11	-0.08	-0.03	0.02	-0.05	-0.00	0.01	0.15	0.00
5	1	0.03	-0.10	-0.11	-0.05	-0.09	0.04	-0.02	-0.19	-0.01
6	16	0.03	-0.01	-0.12	0.04	0.11	0.03	0.02	0.00	-0.02
7	6	-0.02	0.01	-0.07	-0.00	-0.03	0.05	-0.01	-0.05	0.00
8	6	0.00	-0.01	0.03	0.02	0.04	-0.02	0.03	-0.00	0.00
9	6	-0.10	0.05	-0.08	-0.03	-0.11	0.12	-0.06	-0.06	0.03
10	6	-0.05	-0.00	0.13	0.03	0.04	-0.04	0.02	0.03	0.03
11	1	0.06	-0.04	0.04	0.04	0.11	-0.08	0.07	0.01	-0.02
12	6	-0.15	0.06	0.01	-0.02	-0.11	0.11	-0.08	-0.03	0.06
13	1	-0.12	0.07	-0.17	-0.04	-0.16	0.18	-0.10	-0.10	0.03
14	6	-0.12	0.03	0.13	0.01	-0.04	0.03	-0.04	0.01	0.06
15	1	-0.03	-0.03	0.22	0.05	0.10	-0.10	0.05	0.07	0.03
16	1	-0.20	0.09	0.00	-0.04	-0.18	0.17	-0.12	-0.05	0.08

17	1	-0.16	0.03	0.21	0.01	-0.04	0.02	-0.05	0.04	0.08
18	7	0.04	-0.07	0.06	-0.02	-0.01	-0.03	0.04	0.09	0.03
19	6	-0.02	-0.06	0.18	-0.01	-0.04	-0.08	0.07	0.24	0.09
20	1	0.03	-0.05	0.15	-0.03	-0.03	-0.11	0.11	0.21	0.27
21	1	-0.14	-0.12	0.17	0.03	-0.05	-0.08	0.08	0.43	0.07
22	1	-0.00	-0.02	0.29	-0.01	-0.04	-0.09	0.04	0.21	-0.03
23	6	0.18	-0.04	0.05	-0.06	0.05	-0.03	0.06	-0.07	0.04
24	1	0.27	-0.08	-0.03	-0.00	0.01	-0.05	0.16	-0.19	0.12
25	1	0.23	0.04	0.15	-0.05	0.14	-0.07	0.08	0.02	0.06
26	1	0.16	-0.06	0.04	-0.14	0.03	0.02	-0.06	-0.16	-0.06
27	6	0.01	0.09	-0.01	-0.02	0.01	0.06	-0.00	-0.14	-0.01
28	6	0.01	0.03	0.02	-0.01	-0.11	0.13	-0.01	-0.07	-0.04
29	6	0.02	0.03	0.01	-0.01	-0.08	0.07	-0.01	-0.05	-0.02
30	6	-0.00	0.03	-0.01	-0.03	-0.00	-0.03	0.03	0.01	-0.04
31	6	0.03	-0.00	0.02	0.04	-0.09	0.06	-0.06	-0.05	0.00
32	6	-0.00	0.00	-0.02	-0.00	0.09	-0.17	0.04	0.09	-0.04
33	1	-0.01	0.06	-0.03	-0.06	-0.00	-0.01	0.07	0.01	-0.06
34	6	0.03	-0.04	0.02	0.06	0.00	-0.08	-0.05	0.03	-0.00
35	1	0.05	-0.01	0.05	0.05	-0.16	0.15	-0.09	-0.09	0.01
36	6	0.02	-0.04	0.00	0.04	0.10	-0.20	-0.00	0.11	-0.03
37	1	-0.01	0.01	-0.03	-0.02	0.16	-0.26	0.08	0.15	-0.06
38	1	0.05	-0.07	0.04	0.09	0.00	-0.10	-0.09	0.04	0.01
39	1	0.01	-0.06	0.00	0.06	0.18	-0.32	-0.00	0.17	-0.04
40	7	0.01	0.06	0.00	-0.01	0.02	0.01	-0.01	-0.04	-0.03
41	8	0.01	0.17	-0.03	-0.07	0.21	-0.09	0.01	-0.07	-0.01
42	8	0.02	-0.09	0.05	0.04	-0.15	0.04	-0.03	0.08	-0.06
43	1	0.01	0.18	-0.05	-0.06	0.13	0.01	0.01	-0.23	0.03
44	1	0.01	-0.04	0.06	-0.02	-0.19	0.18	0.00	0.01	-0.09

	10	11	12
	A	A	A
Frequencies --	104.9134	113.3648	122.5105
Red. masses --	4.5170	4.7193	6.0140
Frc consts --	0.0293	0.0357	0.0532
IR Inten --	1.8458	5.8122	5.1711

Atom	AN	X	Y	Z	X	Y	Z	X	Y	Z
1	6	-0.01	-0.04	0.01	-0.02	-0.08	0.03	-0.00	0.01	0.01
2	6	-0.02	-0.10	0.01	-0.04	-0.15	0.04	-0.01	0.01	0.01
3	6	0.00	-0.01	-0.01	-0.03	-0.13	0.03	-0.01	-0.04	-0.00
4	1	0.03	0.02	-0.01	-0.02	-0.14	0.03	-0.01	-0.08	-0.01
5	1	-0.03	-0.18	0.01	-0.04	-0.20	0.04	-0.01	0.05	0.01
6	16	0.05	0.14	-0.01	0.01	0.05	0.02	-0.00	-0.02	0.01
7	6	-0.02	-0.06	0.02	-0.03	-0.08	0.04	0.00	0.01	0.00
8	6	-0.04	-0.12	0.09	-0.01	-0.06	0.03	0.00	0.03	-0.02
9	6	0.02	0.06	-0.09	-0.03	-0.05	0.01	0.01	-0.02	0.03
10	6	-0.01	-0.03	0.03	0.01	0.00	-0.01	0.00	0.01	-0.03
11	1	-0.07	-0.23	0.18	-0.01	-0.09	0.05	0.00	0.05	-0.04
12	6	0.06	0.18	-0.17	-0.01	0.02	-0.03	0.00	-0.04	0.03

13	1	0.02	0.08	-0.14	-0.05	-0.07	0.02	0.01	-0.03	0.05
14	6	0.05	0.14	-0.12	0.01	0.05	-0.05	0.00	-0.03	0.01
15	1	-0.02	-0.07	0.09	0.03	0.03	-0.01	0.00	0.02	-0.05
16	1	0.10	0.31	-0.29	-0.01	0.05	-0.06	0.00	-0.07	0.05
17	1	0.08	0.23	-0.19	0.03	0.11	-0.09	0.00	-0.05	0.01
18	7	0.01	0.05	-0.02	-0.03	-0.10	0.02	-0.03	-0.04	-0.02
19	6	0.03	0.08	-0.04	-0.04	-0.18	0.00	-0.02	-0.07	-0.05
20	1	0.00	0.06	0.05	0.03	-0.11	-0.24	-0.04	-0.06	-0.08
21	1	0.08	0.19	-0.04	-0.11	-0.45	0.02	-0.01	-0.10	-0.04
22	1	0.02	0.04	-0.13	-0.05	-0.05	0.20	-0.02	-0.06	-0.03
23	6	-0.01	0.02	-0.02	0.00	0.01	0.02	-0.04	0.04	-0.02
24	1	0.14	-0.12	0.00	0.22	-0.12	-0.07	-0.12	0.12	-0.06
25	1	0.02	0.21	-0.04	0.06	0.31	0.02	-0.06	-0.03	-0.04
26	1	-0.21	-0.06	-0.02	-0.23	-0.04	0.10	0.05	0.10	0.03
27	6	-0.01	-0.07	0.03	0.02	-0.01	0.00	0.08	-0.13	-0.09
28	6	-0.02	-0.09	0.03	0.02	0.01	-0.02	0.01	-0.07	-0.19
29	6	-0.02	-0.08	0.02	0.02	-0.03	0.01	0.00	-0.04	-0.15
30	6	-0.01	-0.11	0.06	0.03	-0.02	0.01	0.11	0.03	-0.11
31	6	-0.03	-0.02	-0.02	0.01	-0.01	0.00	-0.12	-0.05	-0.07
32	6	-0.01	-0.08	0.07	0.03	0.02	-0.00	0.10	0.09	0.03
33	1	0.01	-0.16	0.10	0.03	-0.03	0.02	0.21	0.04	-0.17
34	6	-0.03	0.01	-0.01	0.01	0.02	-0.01	-0.14	-0.00	0.08
35	1	-0.04	0.01	-0.06	0.00	-0.01	0.00	-0.20	-0.08	-0.12
36	6	-0.02	-0.03	0.04	0.03	0.04	-0.02	-0.03	0.06	0.13
37	1	0.00	-0.11	0.11	0.04	0.03	-0.01	0.19	0.14	0.06
38	1	-0.04	0.05	-0.04	0.01	0.04	-0.02	-0.25	-0.01	0.15
39	1	-0.02	-0.01	0.04	0.03	0.08	-0.04	-0.05	0.09	0.24
40	7	-0.02	-0.03	0.03	0.01	0.13	-0.04	0.04	0.03	0.07
41	8	-0.01	0.08	0.01	0.02	0.04	-0.01	0.17	0.26	0.12
42	8	-0.02	-0.11	0.06	-0.01	0.34	-0.10	-0.13	-0.05	0.15
43	1	0.00	-0.05	0.02	0.03	-0.12	0.04	0.20	-0.21	-0.07
44	1	-0.02	-0.10	0.03	0.02	0.10	-0.06	-0.01	0.01	-0.23

	13	14	15
	A	A	A
Frequencies --	130.0548	152.4635	153.4919
Red. masses --	3.0869	1.7593	2.6581
Frc consts --	0.0308	0.0241	0.0369
IR Inten --	2.3946	0.9508	3.1421

Atom	AN	X	Y	Z	X	Y	Z	X	Y	Z
1	6	-0.01	-0.08	0.04	-0.00	-0.04	-0.00	-0.00	-0.03	0.00
2	6	-0.03	-0.14	0.05	-0.00	-0.08	0.01	-0.01	-0.03	-0.01
3	6	0.00	-0.05	0.02	-0.01	-0.09	0.02	-0.02	-0.04	-0.00
4	1	0.03	-0.02	0.02	-0.02	-0.13	0.02	-0.03	-0.06	-0.00
5	1	-0.05	-0.23	0.05	0.01	-0.04	0.01	-0.01	-0.01	-0.01
6	16	0.00	0.01	0.03	0.01	0.02	-0.01	0.04	0.04	-0.01
7	6	-0.01	-0.07	0.03	-0.00	-0.03	-0.00	-0.01	-0.04	0.02
8	6	0.02	-0.02	-0.01	0.01	-0.02	-0.00	0.00	-0.03	0.01

9	6	-0.03	-0.08	0.04	-0.01	-0.02	-0.01	-0.02	-0.04	0.02
10	6	0.03	0.02	-0.03	0.01	0.00	0.00	0.01	0.01	-0.01
11	1	0.03	-0.01	-0.02	0.03	-0.02	-0.00	0.01	-0.03	0.02
12	6	-0.02	-0.05	0.02	-0.02	-0.01	-0.00	-0.01	-0.00	-0.00
13	1	-0.05	-0.10	0.05	-0.03	-0.03	-0.01	-0.03	-0.05	0.02
14	6	0.01	0.00	-0.01	-0.00	0.01	0.00	0.01	0.03	-0.02
15	1	0.05	0.06	-0.06	0.02	0.01	0.01	0.02	0.03	-0.02
16	1	-0.03	-0.06	0.03	-0.03	-0.00	-0.00	-0.02	0.01	-0.01
17	1	0.02	0.03	-0.03	-0.00	0.02	0.01	0.02	0.06	-0.04
18	7	0.00	0.03	-0.01	-0.01	-0.05	0.01	-0.01	-0.02	0.01
19	6	0.02	0.04	-0.04	0.04	0.06	-0.01	0.00	0.03	0.01
20	1	-0.01	0.02	0.02	0.13	0.07	-0.01	0.03	0.03	0.05
21	1	0.08	0.12	-0.04	0.05	0.06	-0.01	0.01	0.08	0.01
22	1	0.02	-0.00	-0.12	-0.02	0.13	-0.03	-0.01	0.04	-0.01
23	6	-0.03	-0.00	-0.00	-0.01	0.10	0.01	0.01	0.07	0.00
24	1	0.28	-0.27	0.02	-0.38	0.43	-0.08	-0.24	0.30	-0.06
25	1	0.03	0.41	-0.04	-0.09	-0.35	0.00	-0.04	-0.24	0.01
26	1	-0.42	-0.15	0.01	0.43	0.30	0.09	0.31	0.21	0.05
27	6	0.01	0.13	-0.05	-0.00	-0.08	0.06	-0.00	0.23	-0.14
28	6	0.01	0.15	-0.06	-0.00	0.06	-0.01	-0.01	-0.07	0.00
29	6	0.01	0.14	-0.07	-0.00	0.06	-0.01	-0.01	-0.05	0.00
30	6	-0.02	0.12	-0.06	-0.02	0.04	-0.01	0.02	-0.03	0.00
31	6	0.02	0.09	-0.03	0.01	0.05	-0.02	-0.03	-0.06	0.02
32	6	-0.03	0.01	-0.01	-0.03	-0.01	0.00	0.02	0.02	-0.01
33	1	-0.04	0.17	-0.10	-0.04	0.05	-0.01	0.04	-0.04	-0.00
34	6	0.01	-0.02	0.02	0.01	0.01	-0.01	-0.03	-0.02	0.02
35	1	0.04	0.12	-0.04	0.03	0.07	-0.03	-0.04	-0.10	0.05
36	6	-0.02	-0.08	0.05	-0.01	-0.03	0.01	-0.00	0.03	0.00
37	1	-0.05	-0.03	0.00	-0.04	-0.03	0.01	0.04	0.06	-0.02
38	1	0.03	-0.08	0.06	0.03	0.00	-0.01	-0.05	-0.03	0.05
39	1	-0.03	-0.18	0.11	-0.01	-0.07	0.02	0.00	0.06	-0.00
40	7	0.01	-0.00	-0.00	0.00	-0.02	0.01	-0.00	0.03	-0.02
41	8	0.02	-0.03	0.02	-0.03	0.02	-0.02	0.06	-0.11	0.07
42	8	0.01	-0.10	0.03	0.03	-0.00	-0.00	-0.06	0.01	0.01
43	1	0.01	0.16	-0.07	-0.01	-0.20	0.11	0.00	0.50	-0.25
44	1	0.00	0.11	-0.05	-0.00	0.18	-0.06	-0.00	-0.34	0.14

	16	17	18
	A	A	A
Frequencies --	175.9462	214.5569	236.0835
Red. masses --	4.8948	2.6583	1.5664
Frc consts --	0.0893	0.0721	0.0514
IR Inten --	3.2684	1.5483	2.5719

Atom	AN	X	Y	Z	X	Y	Z	X	Y	Z
1	6	-0.02	0.01	0.02	-0.00	0.05	0.01	-0.01	0.01	-0.00
2	6	-0.08	0.13	-0.10	-0.04	-0.05	-0.01	-0.02	-0.05	-0.00
3	6	-0.09	0.10	-0.10	-0.06	-0.14	-0.02	-0.03	-0.06	0.01
4	1	-0.15	0.10	-0.10	-0.07	-0.17	-0.02	-0.04	-0.09	0.01

5	1	-0.13	0.16	-0.10	-0.04	0.00	-0.01	-0.01	-0.04	-0.00
6	16	0.20	-0.04	0.02	0.06	-0.03	0.02	0.06	-0.02	0.00
7	6	-0.07	-0.08	0.12	0.00	0.13	0.01	-0.01	0.06	-0.00
8	6	-0.08	-0.07	0.06	-0.04	0.09	-0.03	-0.03	0.05	-0.02
9	6	-0.04	-0.11	0.13	0.06	0.10	0.01	0.02	0.05	-0.00
10	6	-0.01	0.02	-0.08	-0.05	-0.02	-0.03	-0.03	-0.01	-0.02
11	1	-0.12	-0.07	0.08	-0.10	0.13	-0.04	-0.05	0.06	-0.03
12	6	0.02	-0.04	0.01	0.07	0.00	0.00	0.02	0.00	-0.01
13	1	-0.04	-0.14	0.20	0.10	0.12	0.05	0.03	0.06	0.01
14	6	0.05	0.06	-0.13	0.01	-0.07	-0.00	-0.01	-0.04	-0.00
15	1	-0.00	0.07	-0.16	-0.10	-0.07	-0.05	-0.06	-0.03	-0.03
16	1	0.06	-0.04	-0.01	0.13	-0.03	0.02	0.04	-0.02	0.00
17	1	0.11	0.17	-0.26	0.00	-0.17	0.01	-0.02	-0.08	0.01
18	7	-0.06	0.03	-0.02	-0.06	-0.12	-0.01	-0.01	0.00	0.01
19	6	-0.15	-0.00	0.14	0.01	0.15	0.01	-0.03	-0.03	0.03
20	1	-0.12	0.01	0.11	0.35	0.25	-0.16	-0.29	-0.18	0.46
21	1	-0.32	-0.08	0.13	-0.13	-0.09	0.02	0.16	0.50	-0.01
22	1	-0.10	0.02	0.30	-0.15	0.48	0.21	0.06	-0.38	-0.37
23	6	0.07	-0.04	-0.02	0.01	-0.00	-0.02	-0.02	0.04	0.01
24	1	0.01	0.01	-0.03	0.19	-0.11	-0.12	-0.01	0.04	-0.01
25	1	0.08	-0.16	0.08	0.06	0.24	0.01	-0.02	0.07	-0.00
26	1	0.20	-0.02	-0.10	-0.16	-0.04	0.05	-0.05	0.04	0.05
27	6	-0.00	-0.04	0.02	0.00	0.02	0.01	-0.02	-0.02	-0.02
28	6	0.00	0.06	-0.02	-0.01	-0.03	0.02	0.01	0.01	-0.01
29	6	0.00	0.04	-0.02	-0.01	-0.01	-0.00	0.02	0.00	0.02
30	6	-0.01	0.03	-0.02	-0.01	-0.01	-0.00	0.02	-0.00	0.02
31	6	0.01	0.04	-0.02	-0.02	-0.01	-0.00	0.03	-0.00	0.02
32	6	-0.01	-0.01	-0.00	-0.01	-0.00	-0.00	0.02	-0.01	0.01
33	1	-0.01	0.05	-0.03	-0.01	-0.01	0.00	0.01	-0.00	0.02
34	6	0.01	0.00	-0.01	-0.02	0.00	0.00	0.04	-0.00	-0.00
35	1	0.02	0.05	-0.03	-0.02	-0.01	-0.00	0.04	-0.00	0.02
36	6	-0.00	-0.02	0.01	-0.02	0.00	0.00	0.04	-0.00	-0.01
37	1	-0.02	-0.02	0.00	-0.01	0.00	0.00	0.01	-0.01	0.00
38	1	0.02	-0.00	-0.01	-0.02	0.00	0.00	0.05	0.00	-0.01
39	1	-0.01	-0.05	0.02	-0.02	0.00	0.01	0.04	0.00	-0.02
40	7	-0.00	-0.01	-0.00	0.01	-0.00	0.01	-0.03	0.01	-0.01
41	8	-0.01	0.02	-0.02	0.00	-0.01	0.00	-0.01	0.01	-0.00
42	8	0.01	-0.03	0.00	0.02	-0.00	0.00	-0.06	0.03	-0.01
43	1	-0.00	-0.12	0.06	0.00	0.06	-0.01	-0.02	-0.04	-0.01
44	1	0.00	0.14	-0.06	-0.02	-0.07	0.04	0.03	0.03	-0.03

	19	20	21
	A	A	A
Frequencies --	257.3103	259.4782	284.3711
Red. masses --	4.5155	3.9895	4.6229
Frc consts --	0.1761	0.1583	0.2203
IR Inten --	2.2970	1.2921	0.3492

Atom	AN	X	Y	Z	X	Y	Z	X	Y	Z

1	6	-0.01	0.02	0.05	-0.01	0.01	0.02	0.01	-0.02	-0.04
2	6	-0.01	0.03	-0.01	-0.01	-0.00	-0.00	-0.06	0.04	-0.09
3	6	0.02	-0.08	-0.14	0.00	-0.03	-0.03	-0.13	-0.03	-0.06
4	1	0.05	-0.14	-0.14	0.01	-0.06	-0.03	-0.19	-0.07	-0.06
5	1	-0.07	0.10	-0.01	-0.02	0.02	-0.00	-0.08	0.08	-0.09
6	16	-0.16	0.03	0.06	-0.03	0.01	0.02	-0.09	0.02	-0.04
7	6	-0.02	0.01	0.08	-0.02	0.02	0.03	0.10	-0.07	-0.02
8	6	-0.05	-0.02	0.08	-0.04	0.01	0.02	0.19	-0.03	0.05
9	6	0.00	-0.01	0.10	-0.01	0.01	0.04	0.13	-0.05	-0.03
10	6	-0.02	-0.00	0.01	-0.03	-0.00	-0.01	0.18	-0.00	0.11
11	1	-0.09	-0.02	0.09	-0.07	0.01	0.02	0.28	-0.07	0.05
12	6	0.04	-0.00	0.04	0.01	0.00	0.01	0.11	-0.01	0.02
13	1	0.02	-0.02	0.15	-0.00	0.00	0.06	0.12	-0.05	-0.04
14	6	0.04	0.03	-0.04	-0.01	0.00	-0.02	0.16	0.00	0.11
15	1	-0.04	-0.00	-0.02	-0.04	-0.01	-0.03	0.20	-0.00	0.13
16	1	0.07	-0.00	0.03	0.03	-0.01	0.01	0.05	0.03	0.01
17	1	0.08	0.07	-0.11	0.01	0.01	-0.05	0.14	0.02	0.15
18	7	0.04	-0.10	-0.12	0.02	-0.02	-0.02	-0.14	-0.05	-0.03
19	6	0.04	0.03	-0.01	0.00	-0.05	0.01	-0.19	0.06	0.11
20	1	0.07	-0.01	0.19	-0.19	-0.16	0.34	-0.16	0.02	0.34
21	1	0.03	0.25	-0.04	0.13	0.36	-0.02	-0.27	0.29	0.08
22	1	0.03	-0.02	-0.12	0.08	-0.32	-0.29	-0.16	-0.00	0.05
23	6	0.27	-0.04	-0.14	0.09	0.00	-0.03	-0.10	0.04	-0.03
24	1	0.32	-0.01	-0.28	0.10	0.01	-0.08	-0.01	-0.00	-0.10
25	1	0.34	-0.05	0.03	0.11	0.00	0.02	-0.07	0.18	-0.03
26	1	0.36	-0.01	-0.16	0.12	0.01	-0.03	-0.20	0.03	0.03
27	6	-0.03	-0.02	-0.05	0.08	0.05	0.11	0.01	0.00	0.01
28	6	0.01	0.04	-0.03	-0.02	-0.06	0.06	0.00	0.00	0.00
29	6	0.02	0.02	0.03	-0.05	-0.04	-0.06	-0.00	-0.00	-0.00
30	6	0.02	0.00	0.03	-0.05	-0.01	-0.08	-0.00	-0.00	-0.00
31	6	0.04	-0.00	0.03	-0.12	0.01	-0.07	-0.01	-0.00	-0.00
32	6	0.02	-0.00	0.01	-0.06	0.02	-0.02	-0.01	0.00	-0.00
33	1	0.00	0.00	0.04	-0.03	-0.01	-0.09	-0.01	-0.00	-0.00
34	6	0.05	-0.00	0.00	-0.14	0.01	0.00	-0.01	0.00	0.00
35	1	0.06	-0.00	0.05	-0.16	0.00	-0.11	-0.01	-0.00	-0.01
36	6	0.05	0.01	-0.02	-0.13	-0.01	0.04	-0.01	0.00	0.00
37	1	0.00	-0.01	-0.00	-0.01	0.04	0.00	-0.00	0.00	0.00
38	1	0.07	-0.00	-0.00	-0.17	0.00	0.02	-0.01	0.00	-0.00
39	1	0.05	0.02	-0.05	-0.14	-0.03	0.10	-0.01	0.00	0.00
40	7	-0.05	-0.01	-0.02	0.14	0.03	0.04	0.01	0.01	-0.00
41	8	-0.00	0.01	0.01	0.03	-0.01	-0.04	0.00	0.00	-0.01
42	8	-0.11	-0.01	0.00	0.28	0.05	-0.01	0.02	0.01	-0.01
43	1	-0.03	-0.06	-0.02	0.10	0.11	0.08	0.01	-0.01	0.01
44	1	0.06	0.09	-0.08	-0.14	-0.13	0.14	-0.01	0.01	0.00

22 23 24

A A A

Frequencies -- 290.9888 311.2069 352.2840

Red. masses -- 3.5929 3.2142 5.3520
Frc consts -- 0.1792 0.1834 0.3913
IR Inten -- 3.5042 7.4788 17.5984

Atom	AN	X	Y	Z	X	Y	Z	X	Y	Z
1	6	0.01	0.01	-0.01	0.03	0.08	-0.05	-0.01	-0.05	0.13
2	6	0.02	0.03	0.00	0.08	0.23	-0.01	-0.01	0.14	0.09
3	6	0.00	-0.01	0.02	0.02	-0.04	0.06	0.01	0.04	-0.02
4	1	-0.00	-0.03	0.02	-0.02	-0.14	0.06	0.07	0.01	-0.03
5	1	0.02	0.03	0.00	0.12	0.29	-0.01	-0.07	0.16	0.10
6	16	0.01	-0.01	-0.01	0.01	-0.01	-0.05	0.07	0.04	0.19
7	6	-0.00	-0.01	-0.01	-0.01	-0.05	-0.01	0.02	-0.14	-0.08
8	6	-0.00	-0.01	0.00	-0.02	-0.08	0.03	0.11	-0.02	-0.18
9	6	-0.01	-0.01	-0.00	-0.06	-0.08	0.02	-0.04	0.00	-0.22
10	6	-0.00	0.00	-0.00	-0.01	0.01	0.00	0.05	0.02	-0.05
11	1	-0.00	-0.02	0.00	-0.02	-0.12	0.06	0.20	0.04	-0.24
12	6	-0.02	-0.00	-0.00	-0.06	-0.02	0.02	-0.12	0.04	-0.11
13	1	-0.01	-0.00	-0.02	-0.06	-0.09	0.02	-0.08	0.03	-0.37
14	6	-0.01	0.01	-0.01	-0.01	0.07	-0.05	-0.14	-0.07	0.08
15	1	0.00	0.01	-0.00	0.02	0.04	0.01	0.13	0.09	-0.00
16	1	-0.02	-0.00	-0.00	-0.09	-0.03	0.03	-0.17	0.10	-0.15
17	1	-0.00	0.02	-0.01	0.02	0.16	-0.11	-0.23	-0.15	0.26
18	7	-0.01	-0.04	0.02	-0.03	-0.21	0.06	-0.06	-0.10	-0.06
19	6	0.02	0.00	-0.01	0.10	0.04	-0.03	-0.06	0.03	0.02
20	1	0.03	-0.00	0.04	0.21	0.02	0.15	0.01	0.01	0.15
21	1	0.06	0.06	-0.01	0.26	0.27	-0.04	-0.09	0.16	0.00
22	1	-0.00	0.00	-0.07	-0.03	0.08	-0.28	-0.08	0.03	-0.03
23	6	-0.02	0.01	0.02	-0.06	0.03	0.06	0.08	-0.00	-0.08
24	1	0.02	-0.01	-0.01	0.13	-0.06	-0.06	0.16	-0.01	-0.21
25	1	-0.01	0.08	-0.00	-0.03	0.36	-0.01	0.14	0.07	0.03
26	1	-0.07	0.01	0.06	-0.30	0.02	0.25	0.09	0.01	-0.04
27	6	0.04	0.04	-0.05	-0.01	-0.01	0.01	-0.00	0.00	0.01
28	6	0.01	0.22	-0.15	-0.00	-0.03	0.02	-0.00	0.00	0.01
29	6	-0.03	-0.02	0.05	0.00	0.00	-0.01	0.00	-0.01	-0.00
30	6	-0.08	-0.12	0.11	0.01	0.02	-0.02	0.01	-0.01	-0.00
31	6	-0.06	-0.12	0.13	0.01	0.02	-0.02	0.00	-0.00	-0.01
32	6	-0.08	-0.05	0.03	0.01	0.01	-0.01	0.01	0.00	0.00
33	1	-0.11	-0.16	0.16	0.02	0.03	-0.03	0.01	-0.01	0.00
34	6	-0.05	-0.01	0.02	0.01	0.00	-0.00	-0.00	-0.00	-0.00
35	1	-0.07	-0.18	0.18	0.01	0.03	-0.03	-0.00	-0.00	-0.01
36	6	-0.04	0.13	-0.12	0.01	-0.02	0.02	0.01	0.00	0.00
37	1	-0.08	-0.05	0.04	0.01	0.01	-0.01	0.01	0.00	0.00
38	1	-0.03	0.02	-0.01	0.01	0.00	-0.00	-0.01	-0.00	0.00
39	1	-0.01	0.32	-0.30	0.00	-0.05	0.05	0.00	0.00	0.00
40	7	0.07	0.02	-0.02	-0.02	-0.00	0.00	-0.00	0.00	0.00
41	8	0.10	-0.01	0.01	-0.02	0.00	-0.00	-0.01	-0.00	-0.00
42	8	0.06	-0.06	0.01	-0.02	0.01	-0.00	0.00	-0.00	0.00
43	1	0.07	-0.24	0.06	-0.02	0.02	-0.00	-0.00	0.00	0.01

44	1	0.02	0.51	-0.29	-0.00	-0.07	0.04	-0.01	0.00	0.01

TS1B

Zero-point correction= 0.357387 (Hartree/Particle)
Thermal correction to Energy= 0.379124
Thermal correction to Enthalpy= 0.380068
Thermal correction to Gibbs Free Energy= 0.305882
Sum of electronic and zero-point Energies= -1393.573123
Sum of electronic and thermal Energies= -1393.551385
Sum of electronic and thermal Enthalpies= -1393.550441
Sum of electronic and thermal Free Energies= -1393.624628

Center Number	Atomic Number	Atomic Type	Coordinates (Angstroms) X	Y	Z
1	6	0	-0.019828	0.001287	-0.018013
2	6	0	-0.010227	-0.076873	1.337024
3	6	0	1.209679	-0.134191	2.085579
4	1	0	2.119695	0.195423	1.596610
5	1	0	-0.942252	-0.036428	1.890247
6	16	0	1.510262	-0.204748	-0.871366
7	6	0	-1.269809	0.173399	-0.793172
8	6	0	-1.247965	0.760889	-2.061631
9	6	0	-2.501120	-0.250891	-0.277486
10	6	0	-2.420809	0.940713	-2.781903
11	1	0	-0.306567	1.085707	-2.489328
12	6	0	-3.671253	-0.080747	-1.002851
13	1	0	-2.551130	-0.756127	0.680916
14	6	0	-3.637128	0.520862	-2.256346
15	1	0	-2.381451	1.406217	-3.760603
16	1	0	-4.611837	-0.429889	-0.590955
17	1	0	-4.551976	0.652083	-2.823763
18	7	0	1.280240	-0.303694	3.378396
19	6	0	2.496326	0.051527	4.109277
20	1	0	2.669607	-0.670418	4.902043
21	1	0	3.345938	0.020263	3.431034
22	1	0	2.385457	1.051865	4.536337
23	6	0	0.123479	-0.752268	4.145082
24	1	0	-0.289010	-1.652065	3.687255
25	1	0	0.442409	-0.988050	5.156573
26	1	0	-0.645891	0.022799	4.189458
27	6	0	1.872977	-2.273590	1.054292
28	6	0	1.906053	-1.980379	-0.384586
29	6	0	1.025402	-2.905240	-1.190395
30	6	0	1.536445	-3.528821	-2.327068
31	6	0	-0.305404	-3.138166	-0.838866
32	6	0	0.734854	-4.358411	-3.103266
33	1	0	2.570419	-3.359817	-2.612751

34	6	0	-1.110385	-3.959813	-1.616879
35	1	0	-0.723668	-2.669134	0.045653
36	6	0	-0.592930	-4.573289	-2.753249
37	1	0	1.150276	-4.836103	-3.984382
38	1	0	-2.145072	-4.121818	-1.333472
39	1	0	-1.220790	-5.216557	-3.360431
40	7	0	2.986435	-2.164129	1.813425
41	8	0	2.965692	-2.622571	2.981184
42	8	0	3.987865	-1.531786	1.384981
43	1	0	1.114570	-2.907254	1.485597
44	1	0	2.935611	-2.051011	-0.739254

IB

Zero-point correction= 0.357770 (Hartree/Particle)
Thermal correction to Energy= 0.379332
Thermal correction to Enthalpy= 0.380276
Thermal correction to Gibbs Free Energy= 0.306218
Sum of electronic and zero-point Energies= -1393.598766
Sum of electronic and thermal Energies= -1393.577204
Sum of electronic and thermal Enthalpies= -1393.576260
Sum of electronic and thermal Free Energies= -1393.650318

Center Number	Atomic Number	Atomic Type	Coordinates (Angstroms) X	Y	Z
1	6	0	0.364385	-1.169643	-0.635927
2	6	0	-0.682338	-1.639296	0.088696
3	6	0	-2.039931	-1.269435	-0.201443
4	1	0	-2.250701	-0.925777	-1.209002
5	1	0	-0.503075	-2.349736	0.888463
6	16	0	0.069263	0.066871	-1.847596
7	6	0	1.755595	-1.609050	-0.374242
8	6	0	2.685968	-1.689663	-1.414042
9	6	0	2.167645	-1.950334	0.918782
10	6	0	3.984251	-2.117192	-1.172311
11	1	0	2.390869	-1.421907	-2.422589
12	6	0	3.468898	-2.368264	1.161553
13	1	0	1.478442	-1.861493	1.751704
14	6	0	4.381405	-2.457051	0.116181
15	1	0	4.688983	-2.181311	-1.994227
16	1	0	3.772658	-2.615194	2.173087
17	1	0	5.398576	-2.782100	0.305987
18	7	0	-3.082999	-1.671262	0.497248
19	6	0	-4.418975	-1.692657	-0.090226
20	1	0	-5.128906	-1.226119	0.589235
21	1	0	-4.417804	-1.134144	-1.023560
22	1	0	-4.718529	-2.726951	-0.281678
23	6	0	-2.948251	-2.199092	1.846103
24	1	0	-2.162245	-1.666370	2.380881
25	1	0	-3.886101	-2.046940	2.377095
26	1	0	-2.719785	-3.268418	1.831007
27	6	0	-1.687689	1.026168	0.227601
28	6	0	-0.625529	1.469409	-0.670908
29	6	0	0.562391	2.085427	0.018373
30	6	0	1.136599	3.243487	-0.504554
31	6	0	1.123123	1.517413	1.163794
32	6	0	2.247886	3.820149	0.098870
33	1	0	0.712939	3.696240	-1.396017

34	6	0	2.238570	2.087019	1.763205
35	1	0	0.695777	0.616519	1.590667
36	6	0	2.805305	3.241464	1.233350
37	1	0	2.679576	4.722775	-0.320503
38	1	0	2.666080	1.626786	2.647744
39	1	0	3.675197	3.687781	1.703106
40	7	0	-3.002723	1.247633	-0.106664
41	8	0	-3.860317	1.119561	0.786269
42	8	0	-3.318375	1.465614	-1.293969
43	1	0	-1.535771	0.963341	1.293656
44	1	0	-1.030646	2.133918	-1.433155

TS2B

Zero-point correction= 0.357778 (Hartree/Particle)
Thermal correction to Energy= 0.379337
Thermal correction to Enthalpy= 0.380282
Thermal correction to Gibbs Free Energy= 0.306242
Sum of electronic and zero-point Energies= -1393.598759
Sum of electronic and thermal Energies= -1393.577199
Sum of electronic and thermal Enthalpies= -1393.576255
Sum of electronic and thermal Free Energies= -1393.650294

Center Number	Atomic Number	Atomic Type	Coordinates (Angstroms) X	Y	Z
1	6	0	0.364521	-1.169582	-0.635895
2	6	0	-0.682137	-1.639310	0.088780
3	6	0	-2.039793	-1.269558	-0.201226
4	1	0	-2.250710	-0.925918	-1.208759
5	1	0	-0.502759	-2.349717	0.888552
6	16	0	0.069314	0.066828	-1.847655
7	6	0	1.755774	-1.608851	-0.374206
8	6	0	2.686112	-1.689550	-1.414030
9	6	0	2.167905	-1.949892	0.918857
10	6	0	3.984441	-2.116931	-1.172282
11	1	0	2.390951	-1.421979	-2.422608
12	6	0	3.469203	-2.367671	1.161643
13	1	0	1.478727	-1.860964	1.751791
14	6	0	4.381675	-2.456549	0.116248
15	1	0	4.689146	-2.181122	-1.994216
16	1	0	3.773025	-2.614409	2.173205
17	1	0	5.398882	-2.781481	0.306067
18	7	0	-3.082745	-1.671517	0.497571
19	6	0	-4.418807	-1.692932	-0.089708
20	1	0	-5.128655	-1.226515	0.589931
21	1	0	-4.417817	-1.134286	-1.022963
22	1	0	-4.718326	-2.727219	-0.281252
23	6	0	-2.947792	-2.199396	1.846385
24	1	0	-2.161747	-1.666658	2.381089
25	1	0	-3.885583	-2.047321	2.377504
26	1	0	-2.719267	-3.268709	1.831216
27	6	0	-1.687951	1.026123	0.227224
28	6	0	-0.625647	1.469377	-0.671103
29	6	0	0.562162	2.085389	0.018369
30	6	0	1.136255	3.243637	-0.504266
31	6	0	1.122910	1.517172	1.163679
32	6	0	2.247433	3.820303	0.099357
33	1	0	0.712586	3.696541	-1.395648

34	6	0	2.238253	2.086778	1.763282
35	1	0	0.695686	0.616090	1.590277
36	6	0	2.804861	3.241426	1.233735
37	1	0	2.679031	4.723081	-0.319783
38	1	0	2.665784	1.626383	2.647727
39	1	0	3.674670	3.687745	1.703641
40	7	0	-3.002925	1.247549	-0.107303
41	8	0	-3.860707	1.119285	0.785419
42	8	0	-3.318340	1.465455	-1.294685
43	1	0	-1.536224	0.963282	1.293305
44	1	0	-1.030649	2.133887	-1.433411

5

Zero-point correction= 0.360334 (Hartree/Particle)
Thermal correction to Energy= 0.381878
Thermal correction to Enthalpy= 0.382822
Thermal correction to Gibbs Free Energy= 0.307532
Sum of electronic and zero-point Energies= -1393.637414
Sum of electronic and thermal Energies= -1393.615870
Sum of electronic and thermal Enthalpies= -1393.614926
Sum of electronic and thermal Free Energies= -1393.690216

Center Number	Atomic Number	Atomic Type	Coordinates (Angstroms) X	Y	Z
1	6	0	1.663250	0.212568	-0.172782
2	6	0	1.438151	-1.073190	-0.452341
3	6	0	0.113497	-1.792220	-0.497110
4	1	0	-0.153635	-1.949965	-1.547492
5	1	0	2.301333	-1.691222	-0.684921
6	16	0	0.370228	1.389622	0.137641
7	6	0	3.033857	0.776227	-0.080493
8	6	0	3.369803	1.981491	-0.703080
9	6	0	4.017764	0.087917	0.634936
10	6	0	4.666035	2.475234	-0.627225
11	1	0	2.619087	2.525825	-1.265827
12	6	0	5.313142	0.583330	0.709470
13	1	0	3.758128	-0.832867	1.146787
14	6	0	5.641119	1.778280	0.078045
15	1	0	4.915037	3.406711	-1.124098
16	1	0	6.065320	0.039401	1.270691
17	1	0	6.651707	2.167480	0.139438
18	7	0	0.123795	-3.116617	0.121528
19	6	0	0.675337	-4.142193	-0.747023
20	1	0	0.531010	-5.120563	-0.283464
21	1	0	0.149373	-4.142789	-1.703957
22	1	0	1.754420	-4.021403	-0.942110
23	6	0	0.727820	-3.158733	1.444133
24	1	0	0.303443	-2.389167	2.091280
25	1	0	0.513843	-4.126712	1.901790
26	1	0	1.820884	-3.022240	1.428269
27	6	0	-1.021071	-0.960374	0.103374
28	6	0	-1.077061	0.455167	-0.479788
29	6	0	-2.336306	1.209568	-0.124655
30	6	0	-3.176560	1.660832	-1.139872
31	6	0	-2.690830	1.440800	1.205724
32	6	0	-4.354081	2.333683	-0.833582
33	1	0	-2.911944	1.480098	-2.176873

34	6	0	-3.866567	2.112315	1.511846
35	1	0	-2.047130	1.096768	2.009093
36	6	0	-4.700761	2.560839	0.492560
37	1	0	-5.000402	2.678530	-1.633291
38	1	0	-4.132589	2.284973	2.548940
39	1	0	-5.619460	3.084838	0.733050
40	7	0	-2.313362	-1.673263	-0.201848
41	8	0	-3.001854	-2.015604	0.733591
42	8	0	-2.594267	-1.844998	-1.371653
43	1	0	-0.979357	-0.927044	1.189574
44	1	0	-0.985993	0.390132	-1.565233

MCC

Zero-point correction= 0.356939 (Hartree/Particle)
Thermal correction to Energy= 0.380292
Thermal correction to Enthalpy= 0.381237
Thermal correction to Gibbs Free Energy= 0.301568
Sum of electronic and zero-point Energies= -1393.610071
Sum of electronic and thermal Energies= -1393.586717
Sum of electronic and thermal Enthalpies= -1393.585773
Sum of electronic and thermal Free Energies= -1393.665441

Center Number	Atomic Number	Atomic Type	Coordinates (Angstroms) X	Y	Z
1	6	0	1.524983	-0.804312	0.062181
2	6	0	0.862737	-0.704420	-1.170130
3	6	0	-0.450674	-0.986527	-1.549218
4	1	0	-0.828797	-0.420389	-2.397679
5	1	0	1.425107	-0.225034	-1.966259
6	16	0	0.824102	-1.055317	1.578751
7	6	0	2.996605	-0.529872	0.011456
8	6	0	3.896625	-1.430862	0.585093
9	6	0	3.495818	0.619847	-0.605784
10	6	0	5.264045	-1.197469	0.527578
11	1	0	3.517101	-2.321631	1.073805
12	6	0	4.863933	0.863682	-0.641114
13	1	0	2.814256	1.349462	-1.030332
14	6	0	5.753311	-0.046480	-0.082421
15	1	0	5.949939	-1.914793	0.966070
16	1	0	5.233354	1.772112	-1.105279
17	1	0	6.821175	0.141784	-0.116488
18	7	0	-1.299171	-1.898958	-1.112881
19	6	0	-2.669949	-1.929940	-1.607636
20	1	0	-3.361961	-1.903807	-0.763877
21	1	0	-2.863502	-1.065114	-2.238052
22	1	0	-2.838563	-2.845012	-2.180047
23	6	0	-0.923929	-3.005475	-0.253466
24	1	0	-1.144256	-2.789122	0.793092
25	1	0	-1.482686	-3.886012	-0.574036
26	1	0	0.142815	-3.197377	-0.350923
27	6	0	-1.669002	1.714279	-0.234812
28	6	0	-0.642705	1.956623	0.576368
29	6	0	-2.900223	1.025498	0.149310
30	6	0	-4.040447	1.184429	-0.644841
31	6	0	-2.960343	0.181626	1.265332
32	6	0	-5.223878	0.536087	-0.318683
33	1	0	-3.998083	1.823539	-1.521298

34	6	0	-4.141671	-0.470787	1.584682
35	1	0	-2.070463	0.004126	1.859081
36	6	0	-5.276690	-0.291873	0.797351
37	1	0	-6.102853	0.672163	-0.938797
38	1	0	-4.175492	-1.129554	2.445330
39	1	0	-6.197984	-0.805600	1.049376
40	7	0	0.560888	2.573897	0.064640
41	8	0	0.669370	2.788129	-1.133089
42	8	0	1.423968	2.838035	0.883228
43	1	0	-1.585646	2.051961	-1.263667
44	1	0	-0.569079	1.735164	1.629059

TSC

Thermal correction to Energy= 0.378764
Thermal correction to Enthalpy= 0.379708
Thermal correction to Gibbs Free Energy= 0.305805
Sum of electronic and zero-point Energies= -1393.570939
Sum of electronic and thermal Energies= -1393.549292
Sum of electronic and thermal Enthalpies= -1393.548348
Sum of electronic and thermal Free Energies= -1393.622251

Center Number	Atomic Number	Atomic Type	Coordinates (Angstroms) X	Y	Z
1	6	0	1.478254	0.650084	-0.062437
2	6	0	0.678541	1.034007	0.967773
3	6	0	-0.743145	1.255313	0.993977
4	1	0	-1.219943	1.080183	1.952419
5	1	0	1.132017	1.088865	1.955285
6	16	0	0.768224	0.065656	-1.532311
7	6	0	2.946503	0.505277	0.130165
8	6	0	3.827107	0.951688	-0.858111
9	6	0	3.476044	-0.060088	1.293348
10	6	0	5.201451	0.848897	-0.684060
11	1	0	3.431868	1.390686	-1.768642
12	6	0	4.850257	-0.167072	1.465220
13	1	0	2.808445	-0.452617	2.051717
14	6	0	5.718334	0.290186	0.479636
15	1	0	5.869574	1.207003	-1.460157
16	1	0	5.243513	-0.620949	2.368804
17	1	0	6.791293	0.204830	0.614670
18	7	0	-1.434572	2.077294	0.205701
19	6	0	-2.792642	2.467818	0.566401
20	1	0	-3.451158	2.382099	-0.297687
21	1	0	-3.179841	1.820084	1.348792
22	1	0	-2.793705	3.505653	0.914437
23	6	0	-0.875439	2.801042	-0.921371
24	1	0	-1.140411	2.327329	-1.868710
25	1	0	-1.277920	3.815954	-0.906662
26	1	0	0.207333	2.853443	-0.839923
27	6	0	-1.201916	-1.008635	0.464039
28	6	0	-0.392679	-1.398742	-0.640044
29	6	0	-2.624464	-0.784703	0.270488
30	6	0	-3.509313	-0.903125	1.355740
31	6	0	-3.154867	-0.376294	-0.963760
32	6	0	-4.860177	-0.627934	1.213032
33	1	0	-3.121390	-1.214379	2.321664
34	6	0	-4.510639	-0.109588	-1.105415

35	1	0	-2.501129	-0.244567	-1.820328
36	6	0	-5.371103	-0.225456	-0.019640
37	1	0	-5.521512	-0.729319	2.067634
38	1	0	-4.894662	0.205723	-2.070432
39	1	0	-6.428364	-0.010699	-0.131411
40	7	0	0.629918	-2.420172	-0.299929
41	8	0	1.210468	-2.330459	0.765249
42	8	0	0.828823	-3.298168	-1.118652
43	1	0	-0.874279	-1.343689	1.439506
44	1	0	-0.893720	-1.743024	-1.537092

6

Zero-point correction= 0.360374 (Hartree/Particle)
Thermal correction to Energy= 0.381707
Thermal correction to Enthalpy= 0.382651
Thermal correction to Gibbs Free Energy= 0.308862
Sum of electronic and zero-point Energies= -1393.623966
Sum of electronic and thermal Energies= -1393.602633
Sum of electronic and thermal Enthalpies= -1393.601689
Sum of electronic and thermal Free Energies= -1393.675478

Center Number	Atomic Number	Atomic Type	Coordinates (Angstroms) X	Y	Z
1	6	0	-1.899461	-0.130250	0.141743
2	6	0	-1.143834	-1.222182	0.253881
3	6	0	0.357563	-1.320527	0.270443
4	1	0	0.610837	-1.911089	1.157989
5	1	0	-1.653694	-2.180308	0.299180
6	16	0	-1.274729	1.520868	-0.042517
7	6	0	-3.384051	-0.177670	0.175943
8	6	0	-4.155026	0.481345	-0.785498
9	6	0	-4.028937	-0.905989	1.178756
10	6	0	-5.541239	0.397444	-0.752617
11	1	0	-3.669590	1.045361	-1.574772
12	6	0	-5.415143	-0.988727	1.208986
13	1	0	-3.437764	-1.398142	1.943656
14	6	0	-6.174987	-0.337340	0.243503
15	1	0	-6.127268	0.905197	-1.511017
16	1	0	-5.902513	-1.556006	1.994598
17	1	0	-7.257586	-0.398733	0.269481
18	7	0	0.780632	-2.131166	-0.856626
19	6	0	1.982657	-2.928558	-0.713777
20	1	0	2.912272	-2.385463	-0.935334
21	1	0	2.054011	-3.315235	0.304964
22	1	0	1.925169	-3.785101	-1.393663
23	6	0	0.598006	-1.594801	-2.186082
24	1	0	1.397487	-0.904073	-2.500383
25	1	0	0.580818	-2.414891	-2.909977
26	1	0	-0.360553	-1.075361	-2.257577
27	6	0	1.043725	0.059519	0.533002
28	6	0	0.487918	1.166393	-0.357992
29	6	0	2.554839	-0.058947	0.475275
30	6	0	3.214919	-0.578911	1.588976
31	6	0	3.307075	0.268885	-0.651214
32	6	0	4.586176	-0.791801	1.571308
33	1	0	2.644769	-0.826412	2.479920

34	6	0	4.682899	0.066260	-0.669386
35	1	0	2.836146	0.678611	-1.537830
36	6	0	5.326135	-0.472533	0.437252
37	1	0	5.079086	-1.202135	2.446170
38	1	0	5.250151	0.327716	-1.556151
39	1	0	6.398371	-0.635007	0.420702
40	7	0	1.203476	2.476622	-0.081253
41	8	0	1.360951	2.806592	1.072243
42	8	0	1.566706	3.113157	-1.048165
43	1	0	0.773224	0.333885	1.557230
44	1	0	0.636865	0.997476	-1.419395

MCD

Zero-point correction= 0.357520 (Hartree/Particle)
Thermal correction to Energy= 0.380576
Thermal correction to Enthalpy= 0.381520
Thermal correction to Gibbs Free Energy= 0.303808
Sum of electronic and zero-point Energies= -1393.611397
Sum of electronic and thermal Energies= -1393.588340
Sum of electronic and thermal Enthalpies= -1393.587396
Sum of electronic and thermal Free Energies= -1393.665109

Center Number	Atomic Number	Atomic Type	Coordinates (Angstroms) X	Y	Z
1	6	0	0.145960	-1.384182	0.061053
2	6	0	-0.539199	-1.079740	-1.125708
3	6	0	-1.901517	-0.932811	-1.394287
4	1	0	-2.159569	-0.261276	-2.210093
5	1	0	0.080579	-0.806722	-1.975169
6	16	0	-0.503440	-1.401426	1.619798
7	6	0	1.622235	-1.589248	-0.083767
8	6	0	2.244641	-2.673377	0.541364
9	6	0	2.407429	-0.708700	-0.831209
10	6	0	3.610863	-2.879826	0.408339
11	1	0	1.646829	-3.359016	1.131374
12	6	0	3.778866	-0.901758	-0.943926
13	1	0	1.956447	0.161188	-1.295046
14	6	0	4.385676	-1.991209	-0.331298
15	1	0	4.073493	-3.735191	0.889609
16	1	0	4.373055	-0.189344	-1.506780
17	1	0	5.455608	-2.145361	-0.423734
18	7	0	-2.949231	-1.542912	-0.870215
19	6	0	-4.301741	-1.116182	-1.210916
20	1	0	-4.267738	-0.297582	-1.925110
21	1	0	-4.857546	-1.954378	-1.635379
22	1	0	-4.810330	-0.765466	-0.310739
23	6	0	-2.859218	-2.730579	-0.041372
24	1	0	-3.681024	-3.394176	-0.314142
25	1	0	-1.911238	-3.231939	-0.226006
26	1	0	-2.920420	-2.482830	1.019099
27	6	0	-0.874880	2.131223	-0.120510
28	6	0	-1.705256	1.761143	0.853853
29	1	0	-1.311773	2.316157	-1.097127
30	1	0	-1.463671	1.530481	1.878509
31	6	0	0.568263	2.307635	-0.002849
32	6	0	1.263826	2.828606	-1.100122
33	6	0	1.284836	1.956114	1.148141

34	6	0	2.640114	3.003324	-1.048538
35	1	0	0.719040	3.096451	-2.000102
36	6	0	2.658325	2.128609	1.196341
37	1	0	0.774584	1.521127	1.999378
38	6	0	3.339061	2.653234	0.100915
39	1	0	3.166131	3.408105	-1.906001
40	1	0	3.204282	1.839743	2.087278
41	1	0	4.415362	2.780168	0.142014
42	7	0	-3.105215	1.561682	0.566459
43	8	0	-3.790847	1.118428	1.473550
44	8	0	-3.541107	1.827526	-0.544216

TSD

Zero-point correction= 0.357387 (Hartree/Particle)
Thermal correction to Energy= 0.379124
Thermal correction to Enthalpy= 0.380068
Thermal correction to Gibbs Free Energy= 0.305882
Sum of electronic and zero-point Energies= -1393.573123
Sum of electronic and thermal Energies= -1393.551385
Sum of electronic and thermal Enthalpies= -1393.550441
Sum of electronic and thermal Free Energies= -1393.624628

Center Number	Atomic Number	Atomic Type	Coordinates (Angstroms) X	Y	Z
1	6	0	0.860380	1.003583	0.377636
2	6	0	0.228010	0.303502	1.359223
3	6	0	-1.180672	0.160398	1.612107
4	1	0	-1.457324	-0.781065	2.081170
5	1	0	0.837662	-0.382931	1.940534
6	16	0	-0.017465	1.801890	-0.873052
7	6	0	2.343354	0.927490	0.252530
8	6	0	3.081984	2.072261	-0.057617
9	6	0	3.026249	-0.277645	0.444198
10	6	0	4.465693	2.017239	-0.165379
11	1	0	2.569212	3.017132	-0.205182
12	6	0	4.409699	-0.333520	0.329847
13	1	0	2.471283	-1.185604	0.654222
14	6	0	5.134901	0.813585	0.027227
15	1	0	5.022161	2.918594	-0.399244
16	1	0	4.920658	-1.280318	0.469703
17	1	0	6.215071	0.769112	-0.061678
18	7	0	-2.047982	1.148037	1.865089
19	6	0	-3.429262	0.811693	2.160076
20	1	0	-3.513200	-0.250056	2.392711
21	1	0	-3.772437	1.386591	3.022833
22	1	0	-4.069015	1.035384	1.303687
23	6	0	-1.746837	2.562967	1.777561
24	1	0	-2.223149	3.066039	2.622491
25	1	0	-0.672721	2.719481	1.838270
26	1	0	-2.120862	2.996891	0.847986
27	6	0	-1.642708	-0.771422	-0.368612
28	6	0	-1.368170	0.176042	-1.374247
29	1	0	-2.665570	-0.784163	-0.011785
30	1	0	-0.826255	-0.124364	-2.260494
31	6	0	-0.904204	-2.033048	-0.310864
32	6	0	-1.429937	-3.107814	0.420188
33	6	0	0.352371	-2.199637	-0.911164

34	6	0	-0.731490	-4.301411	0.543922
35	1	0	-2.403795	-3.004134	0.891102
36	6	0	1.047375	-3.394867	-0.789107
37	1	0	0.807893	-1.383888	-1.463786
38	6	0	0.512638	-4.452860	-0.059950
39	1	0	-1.162855	-5.118460	1.113188
40	1	0	2.018961	-3.496525	-1.262021
41	1	0	1.059287	-5.384742	0.035571
42	7	0	-2.510739	1.010552	-1.802067
43	8	0	-2.484578	1.418629	-2.947628
44	8	0	-3.393018	1.264802	-1.005753

7

Zero-point correction= 0.360572 (Hartree/Particle)
Thermal correction to Energy= 0.381640
Thermal correction to Enthalpy= 0.382584
Thermal correction to Gibbs Free Energy= 0.309394
Sum of electronic and zero-point Energies= -1393.628550
Sum of electronic and thermal Energies= -1393.607482
Sum of electronic and thermal Enthalpies= -1393.606537
Sum of electronic and thermal Free Energies= -1393.679728

Center Number	Atomic Number	Atomic Type	Coordinates (Angstroms) X	Y	Z
1	6	0	1.384957	0.217426	-0.089770
2	6	0	0.733045	-0.259651	-1.150047
3	6	0	-0.728641	-0.570215	-1.297129
4	1	0	-1.120403	0.205641	-1.965762
5	1	0	1.308142	-0.442193	-2.054032
6	16	0	0.620963	0.504477	1.488507
7	6	0	2.827193	0.563871	-0.131600
8	6	0	3.705988	0.100453	0.851429
9	6	0	3.327742	1.345861	-1.175110
10	6	0	5.059985	0.400467	0.780261
11	1	0	3.328974	-0.516527	1.659893
12	6	0	4.682678	1.646174	-1.243153
13	1	0	2.647115	1.728097	-1.928702
14	6	0	5.551992	1.174414	-0.265899
15	1	0	5.733704	0.024865	1.542886
16	1	0	5.057862	2.257111	-2.057165
17	1	0	6.609253	1.411456	-0.317067
18	7	0	-0.888147	-1.806254	-2.056242
19	6	0	-2.224592	-1.984035	-2.589377
20	1	0	-2.540243	-1.081290	-3.117962
21	1	0	-2.221993	-2.811963	-3.302364
22	1	0	-2.984599	-2.213952	-1.821975
23	6	0	-0.398702	-3.009314	-1.410117
24	1	0	-0.340418	-3.814867	-2.145857
25	1	0	0.604656	-2.841999	-1.015288
26	1	0	-1.045025	-3.361999	-0.588824
27	6	0	-1.583409	-0.461699	0.006748
28	6	0	-0.803975	-0.558683	1.338898
29	1	0	-2.268787	-1.312220	0.022291
30	1	0	-1.474319	-0.296350	2.156425
31	6	0	-2.440833	0.790293	0.043142
32	6	0	-3.768501	0.701060	0.459383
33	6	0	-1.929839	2.039766	-0.310542

34	6	0	-4.572610	1.833463	0.519676
35	1	0	-4.179947	-0.265718	0.735549
36	6	0	-2.731711	3.173249	-0.251409
37	1	0	-0.898415	2.134489	-0.635672
38	6	0	-4.055072	3.073736	0.164272
39	1	0	-5.605142	1.744641	0.840199
40	1	0	-2.319997	4.137278	-0.530521
41	1	0	-4.680667	3.958788	0.208124
42	7	0	-0.475381	-2.030323	1.637310
43	8	0	0.669068	-2.412492	1.647759
44	8	0	-1.445898	-2.732344	1.844028

TS1E

Zero-point correction= 0.355297 (Hartree/Particle)
Thermal correction to Energy= 0.376405
Thermal correction to Enthalpy= 0.377349
Thermal correction to Gibbs Free Energy= 0.303632
Sum of electronic and zero-point Energies= -1393.570456
Sum of electronic and thermal Energies= -1393.549348
Sum of electronic and thermal Enthalpies= -1393.548404
Sum of electronic and thermal Free Energies= -1393.622121

Center Number	Atomic Number	Atomic Type	Coordinates (Angstroms) X	Y	Z
1	6	0	-1.678485	0.008362	-0.161830
2	6	0	-1.422497	1.274118	-0.508584
3	6	0	-0.082080	1.923893	-0.452744
4	1	0	-0.026478	2.706998	-1.204822
5	1	0	-2.243816	1.891060	-0.860429
6	16	0	-0.395581	-1.090558	0.363848
7	6	0	-3.053085	-0.547634	-0.135487
8	6	0	-3.334851	-1.814341	-0.654587
9	6	0	-4.095708	0.207063	0.409729
10	6	0	-4.634892	-2.302336	-0.651031
11	1	0	-2.534743	-2.411514	-1.077809
12	6	0	-5.394694	-0.284700	0.416275
13	1	0	-3.882012	1.177111	0.846090
14	6	0	-5.668075	-1.539824	-0.116169
15	1	0	-4.841336	-3.282004	-1.068239
16	1	0	-6.192911	0.310141	0.847000
17	1	0	-6.681847	-1.925477	-0.108798
18	7	0	0.271408	2.613022	0.887584
19	6	0	0.400335	4.070501	0.793236
20	1	0	0.839251	4.449049	1.716364
21	1	0	1.053958	4.313460	-0.041893
22	1	0	-0.579168	4.536403	0.646304
23	6	0	-0.511988	2.194839	2.052208
24	1	0	-0.486151	1.109444	2.144119
25	1	0	-0.064108	2.635368	2.942592
26	1	0	-1.550755	2.525041	1.969759
27	6	0	1.162673	1.041229	-0.535115
28	6	0	1.055217	-0.467417	-0.588828
29	6	0	2.240093	-1.219459	-0.013851
30	6	0	2.706023	-2.363859	-0.656822
31	6	0	2.840757	-0.824271	1.179678
32	6	0	3.762046	-3.094960	-0.125802
33	1	0	2.245667	-2.679013	-1.588522

34	6	0	3.897707	-1.551929	1.712290
35	1	0	2.485001	0.063709	1.691731
36	6	0	4.362290	-2.689949	1.061022
37	1	0	4.118439	-3.979855	-0.642517
38	1	0	4.360256	-1.228628	2.638988
39	1	0	5.188945	-3.256815	1.476006
40	7	0	2.127621	1.547074	-1.467706
41	8	0	2.170952	2.761170	-1.691160
42	8	0	2.932963	0.766421	-1.967794
43	1	0	1.294514	1.834138	0.663786
44	1	0	0.897364	-0.797036	-1.620323

IE

Zero-point correction= 0.361816 (Hartree/Particle)
Thermal correction to Energy= 0.382868
Thermal correction to Enthalpy= 0.383812
Thermal correction to Gibbs Free Energy= 0.310790
Sum of electronic and zero-point Energies= -1393.611540
Sum of electronic and thermal Energies= -1393.590488
Sum of electronic and thermal Enthalpies= -1393.589543
Sum of electronic and thermal Free Energies= -1393.662566

Center Number	Atomic Number	Atomic Type	Coordinates (Angstroms) X	Y	Z
1	6	0	-1.631106	-0.107949	-0.234830
2	6	0	-1.431398	1.151209	-0.637706
3	6	0	-0.119104	1.863646	-0.652405
4	1	0	-0.087043	2.543970	-1.509734
5	1	0	-2.287957	1.719213	-0.986787
6	16	0	-0.306262	-1.171170	0.238116
7	6	0	-2.990804	-0.692126	-0.126369
8	6	0	-3.271248	-1.976659	-0.599660
9	6	0	-4.018769	0.054285	0.456751
10	6	0	-4.558229	-2.491328	-0.513190
11	1	0	-2.482580	-2.566586	-1.053231
12	6	0	-5.303928	-0.464303	0.546916
13	1	0	-3.802829	1.039339	0.857158
14	6	0	-5.577261	-1.737901	0.060029
15	1	0	-4.765431	-3.484910	-0.895499
16	1	0	-6.090666	0.123885	1.006832
17	1	0	-6.580146	-2.144660	0.132393
18	7	0	-0.044434	2.873256	0.523448
19	6	0	-1.031400	3.968393	0.410426
20	1	0	-0.795713	4.733133	1.147958
21	1	0	-0.975152	4.396065	-0.589084
22	1	0	-2.031172	3.578255	0.594236
23	6	0	-0.070235	2.236432	1.856546
24	1	0	0.694281	1.464854	1.888626
25	1	0	0.130909	2.993424	2.611983
26	1	0	-1.050628	1.792082	2.021903
27	6	0	1.116160	1.029995	-0.661426
28	6	0	1.110341	-0.467988	-0.724951
29	6	0	2.314618	-1.182392	-0.140119
30	6	0	2.796536	-2.330474	-0.761552
31	6	0	2.904423	-0.762129	1.050806
32	6	0	3.857668	-3.042899	-0.211390
33	1	0	2.344035	-2.668414	-1.689394

34	6	0	3.963357	-1.468585	1.601617
35	1	0	2.544422	0.138208	1.537844
36	6	0	4.444404	-2.613893	0.972132
37	1	0	4.225545	-3.932789	-0.711391
38	1	0	4.419497	-1.122988	2.523492
39	1	0	5.273703	-3.165368	1.402417
40	7	0	2.234307	1.705530	-0.972886
41	8	0	2.214099	2.991715	-0.879548
42	8	0	3.285819	1.135806	-1.330803
43	1	0	0.900734	3.258513	0.332095
44	1	0	0.963773	-0.820195	-1.752549

TS2E

Zero-point correction= 0.358918 (Hartree/Particle)
Thermal correction to Energy= 0.380331
Thermal correction to Enthalpy= 0.381275
Thermal correction to Gibbs Free Energy= 0.307114
Sum of electronic and zero-point Energies= -1393.602359
Sum of electronic and thermal Energies= -1393.580946
Sum of electronic and thermal Enthalpies= -1393.580002
Sum of electronic and thermal Free Energies= -1393.654163

Center Number	Atomic Number	Atomic Type	Coordinates (Angstroms) X	Y	Z
1	6	0	-1.620088	-0.119546	-0.373348
2	6	0	-1.383830	1.137251	-0.782433
3	6	0	-0.058264	1.740879	-0.837364
4	1	0	0.005142	2.684535	-1.364683
5	1	0	-2.223865	1.760415	-1.068822
6	16	0	-0.321686	-1.223829	0.067225
7	6	0	-2.990519	-0.652208	-0.196257
8	6	0	-3.330073	-1.941394	-0.616921
9	6	0	-3.972725	0.146710	0.398000
10	6	0	-4.628603	-2.410122	-0.466995
11	1	0	-2.579957	-2.570769	-1.082590
12	6	0	-5.269087	-0.326132	0.550615
13	1	0	-3.712707	1.136358	0.758754
14	6	0	-5.600935	-1.605162	0.116637
15	1	0	-4.881429	-3.407736	-0.809119
16	1	0	-6.019167	0.302484	1.018079
17	1	0	-6.613170	-1.975433	0.237718
18	7	0	-0.040704	2.815820	0.884397
19	6	0	-0.959530	3.944765	0.829907
20	1	0	-0.846082	4.604671	1.695949
21	1	0	-0.774230	4.520983	-0.078017
22	1	0	-1.985468	3.571419	0.807651
23	6	0	-0.209498	1.963982	2.051229
24	1	0	0.489833	1.130499	1.992139
25	1	0	-0.040099	2.512704	2.983065
26	1	0	-1.224411	1.561519	2.056623
27	6	0	1.096388	0.959700	-0.887749
28	6	0	1.148690	-0.542152	-0.829133
29	6	0	2.330285	-1.155527	-0.098540
30	6	0	2.818730	-2.389405	-0.520592
31	6	0	2.904416	-0.550027	1.017834
32	6	0	3.862822	-3.009256	0.157562
33	1	0	2.382622	-2.868889	-1.392348

34	6	0	3.950176	-1.163191	1.693877
35	1	0	2.545928	0.418772	1.350717
36	6	0	4.431869	-2.397437	1.267593
37	1	0	4.233690	-3.969033	-0.186308
38	1	0	4.393756	-0.674228	2.554815
39	1	0	5.249489	-2.875948	1.796076
40	7	0	2.254293	1.623891	-1.288513
41	8	0	2.309918	2.864646	-1.177825
42	8	0	3.202004	0.970228	-1.737033
43	1	0	0.918519	3.135011	0.797785
44	1	0	1.116735	-0.963400	-1.838953

3

Zero-point correction= 0.262282 (Hartree/Particle)
Thermal correction to Energy= 0.279336
Thermal correction to Enthalpy= 0.280280
Thermal correction to Gibbs Free Energy= 0.214462
Sum of electronic and zero-point Energies= -1258.548201
Sum of electronic and thermal Energies= -1258.531147
Sum of electronic and thermal Enthalpies= -1258.530203
Sum of electronic and thermal Free Energies= -1258.596021

Center Number	Atomic Number	Atomic Type	Coordinates (Angstroms) X	Y	Z
1	6	0	-1.693354	0.465355	-0.086150
2	6	0	-1.388927	1.780050	-0.074618
3	6	0	-0.039151	2.271939	-0.017868
4	1	0	0.109234	3.325143	0.189410
5	1	0	-2.191805	2.508045	-0.079201
6	16	0	-0.454526	-0.777910	-0.065782
7	6	0	-3.081157	-0.038523	-0.007412
8	6	0	-3.500842	-1.125597	-0.780765
9	6	0	-3.998882	0.580614	0.848218
10	6	0	-4.815056	-1.568941	-0.713404
11	1	0	-2.803631	-1.609657	-1.455440
12	6	0	-5.310229	0.131719	0.915759
13	1	0	-3.675514	1.401359	1.479148
14	6	0	-5.722201	-0.942751	0.133947
15	1	0	-5.130780	-2.405216	-1.327338
16	1	0	-6.009830	0.616119	1.588017
17	1	0	-6.746892	-1.293826	0.188795
18	6	0	1.049972	1.520031	-0.236230
19	6	0	1.062314	0.082786	-0.687051
20	6	0	2.218319	-0.747686	-0.164825
21	6	0	2.891634	-1.610981	-1.023530
22	6	0	2.593606	-0.688279	1.176916
23	6	0	3.934975	-2.399658	-0.552131
24	1	0	2.605953	-1.660848	-2.069704
25	6	0	3.641253	-1.468977	1.646509
26	1	0	2.065907	-0.027623	1.858482
27	6	0	4.313895	-2.328085	0.783033
28	1	0	4.454521	-3.067031	-1.231215
29	1	0	3.931345	-1.408609	2.689854
30	1	0	5.130955	-2.939285	1.150860
31	7	0	2.328450	2.221297	-0.283490
32	8	0	2.477295	3.226619	0.388847
33	8	0	3.178398	1.766341	-1.029868

34	1	0	1.051249	0.037128	-1.778936

TSF

Zero-point correction= 0.512458 (Hartree/Particle)
Thermal correction to Energy= 0.541561
Thermal correction to Enthalpy= 0.542505
Thermal correction to Gibbs Free Energy= 0.449387
Sum of electronic and zero-point Energies= -1652.556430
Sum of electronic and thermal Energies= -1652.527327
Sum of electronic and thermal Enthalpies= -1652.526382
Sum of electronic and thermal Free Energies= -1652.619500

Center Number	Atomic Number	Atomic Type	Coordinates (Angstroms) X	Y	Z
1	6	0	-2.711433	-0.370714	0.053687
2	6	0	-1.913204	-1.168828	0.765445
3	6	0	-0.407637	-1.125984	0.886162
4	1	0	-0.164613	-1.183516	1.949736
5	1	0	-2.381013	-2.000791	1.283229
6	16	0	-2.095512	1.108508	-0.703423
7	6	0	-4.156557	-0.644233	-0.138040
8	6	0	-5.113464	0.369026	-0.032919
9	6	0	-4.580440	-1.945442	-0.424413
10	6	0	-6.463329	0.081556	-0.188919
11	1	0	-4.801438	1.383517	0.189754
12	6	0	-5.930496	-2.230869	-0.580824
13	1	0	-3.843765	-2.733518	-0.540592
14	6	0	-6.876120	-1.218056	-0.462656
15	1	0	-7.195385	0.876269	-0.093820
16	1	0	-6.243101	-3.244746	-0.806421
17	1	0	-7.930402	-1.439478	-0.589141
18	6	0	-0.026279	-3.519501	1.054043
19	1	0	0.535095	-4.346405	0.618404
20	1	0	0.333213	-3.367783	2.073336
21	1	0	-1.085459	-3.810171	1.088031
22	6	0	-0.177098	-2.524215	-1.124684
23	1	0	0.000536	-1.623868	-1.714985
24	1	0	0.439734	-3.320867	-1.543115
25	1	0	-1.234227	-2.801251	-1.232859
26	6	0	0.245776	0.169321	0.374735
27	6	0	-0.688068	1.391824	0.424881
28	6	0	-0.019240	2.695326	0.062050
29	6	0	0.083530	3.702204	1.018270
30	6	0	0.513471	2.907030	-1.210687
31	6	0	0.711917	4.904366	0.711188
32	1	0	-0.326544	3.545731	2.011179
33	6	0	1.137118	4.107678	-1.518650

34	1	0	0.447569	2.129472	-1.965445
35	6	0	1.238453	5.109421	-0.557691
36	1	0	0.788070	5.679797	1.465583
37	1	0	1.548149	4.261332	-2.510442
38	1	0	1.728279	6.046580	-0.799050
39	7	0	1.444212	0.531643	1.238259
40	8	0	2.414199	0.983342	0.672395
41	8	0	1.334707	0.428782	2.443519
42	1	0	0.667565	0.059085	-0.620280
43	1	0	-1.107685	1.461366	1.431293
44	7	0	0.215450	-2.316369	0.264535
45	1	0	2.500063	-1.430310	-0.639605
46	1	0	2.536596	-1.990420	1.373693
47	1	0	2.520823	-3.434030	-0.126486
48	5	0	2.559201	-2.279763	0.207720
49	6	0	5.145530	-0.984650	0.636609
50	8	0	4.625795	-2.241666	0.177094
51	6	0	5.153323	-2.528826	-1.130791
52	6	0	6.030769	-1.337086	-1.520510
53	6	0	5.491133	-0.217995	-0.627072
54	1	0	4.376681	-0.512205	1.245810
55	1	0	6.033874	-1.173576	1.250244
56	1	0	5.703772	-3.470694	-1.084232
57	1	0	4.303214	-2.649601	-1.807026
58	1	0	7.079499	-1.536395	-1.283483
59	1	0	5.962402	-1.109808	-2.585342
60	1	0	6.215011	0.578967	-0.449819
61	1	0	4.584483	0.221304	-1.050895

IF

Zero-point correction= 0.395776 (Hartree/Particle)
Thermal correction to Energy= 0.418299
Thermal correction to Enthalpy= 0.419243
Thermal correction to Gibbs Free Energy= 0.343173
Sum of electronic and zero-point Energies= -1420.262484
Sum of electronic and thermal Energies= -1420.239961
Sum of electronic and thermal Enthalpies= -1420.239017
Sum of electronic and thermal Free Energies= -1420.315087

Center Number	Atomic Number	Atomic Type	Coordinates (Angstroms) X	Y	Z
1	6	0	-1.649093	-0.276512	-0.058071
2	6	0	-1.404803	0.914825	-0.612891
3	6	0	-0.148406	1.738380	-0.498395
4	1	0	0.045751	2.178689	-1.476847
5	1	0	-2.197519	1.361373	-1.202832
6	16	0	-0.353708	-1.199076	0.717019
7	6	0	-2.987390	-0.912294	-0.090550
8	6	0	-3.142410	-2.275019	-0.359302
9	6	0	-4.125183	-0.135216	0.146678
10	6	0	-4.409497	-2.840528	-0.412329
11	1	0	-2.269521	-2.890600	-0.545657
12	6	0	-5.390998	-0.703560	0.096354
13	1	0	-4.015365	0.916472	0.389658
14	6	0	-5.536373	-2.057629	-0.184994
15	1	0	-4.516408	-3.896876	-0.633388
16	1	0	-6.264254	-0.089519	0.287542
17	1	0	-6.524674	-2.502904	-0.221099
18	6	0	-1.472631	3.765222	-0.138675
19	1	0	-1.537779	4.680027	0.444498
20	1	0	-1.247330	4.020037	-1.173192
21	1	0	-2.423794	3.236871	-0.082000
22	6	0	-0.736694	2.529177	1.790739
23	1	0	0.070164	1.957438	2.243120
24	1	0	-0.890494	3.430897	2.380049
25	1	0	-1.649192	1.932258	1.783236
26	6	0	1.123034	0.959121	-0.107994
27	6	0	1.012638	-0.559159	-0.315484
28	6	0	2.282576	-1.307117	0.008645
29	6	0	2.939916	-2.006556	-1.000192
30	6	0	2.819214	-1.302077	1.297169
31	6	0	4.120257	-2.689836	-0.728960
32	1	0	2.530558	-2.014424	-2.005519
33	6	0	3.994896	-1.987330	1.568699

34	1	0	2.322737	-0.758602	2.095368
35	6	0	4.648707	-2.682219	0.555594
36	1	0	4.625076	-3.227885	-1.523752
37	1	0	4.403801	-1.976434	2.572999
38	1	0	5.569053	-3.214998	0.768663
39	7	0	2.294301	1.405874	-0.970423
40	8	0	3.360508	1.557800	-0.423449
41	8	0	2.104298	1.500896	-2.167155
42	1	0	1.448666	1.167667	0.905504
43	1	0	0.725695	-0.748894	-1.351995
44	7	0	-0.364705	2.939678	0.414137
45	1	0	1.826358	3.291282	1.083427
46	1	0	1.286794	4.086748	-0.693618
47	1	0	0.636645	4.900887	1.042407
48	5	0	0.970439	3.885448	0.464741

TS1G

Zero-point correction= 0.510445 (Hartree/Particle)
Thermal correction to Energy= 0.540377
Thermal correction to Enthalpy= 0.541321
Thermal correction to Gibbs Free Energy= 0.446521
Sum of electronic and zero-point Energies= -1652.551395
Sum of electronic and thermal Energies= -1652.521464
Sum of electronic and thermal Enthalpies= -1652.520519
Sum of electronic and thermal Free Energies= -1652.615319

Center Number	Atomic Number	Atomic Type	Coordinates (Angstroms) X	Y	Z
1	6	0	3.180254	0.203409	-0.208602
2	6	0	2.902045	-1.070395	-0.496399
3	6	0	1.553938	-1.745746	-0.487417
4	1	0	1.232291	-1.880697	-1.525700
5	1	0	3.733050	-1.711904	-0.777257
6	16	0	1.943374	1.418158	0.179785
7	6	0	4.571204	0.721460	-0.169496
8	6	0	4.920135	1.921098	-0.795679
9	6	0	5.560438	-0.005749	0.498382
10	6	0	6.234074	2.371225	-0.770401
11	1	0	4.165044	2.495144	-1.321899
12	6	0	6.873463	0.446290	0.522455
13	1	0	5.291825	-0.922154	1.013401
14	6	0	7.214058	1.635768	-0.112551
15	1	0	6.492951	3.298732	-1.269585
16	1	0	7.629964	-0.127253	1.047152
17	1	0	8.238682	1.990963	-0.090596
18	6	0	1.995033	-4.115428	-0.789213
19	1	0	1.837846	-5.090874	-0.324038
20	1	0	1.412058	-4.085412	-1.711996
21	1	0	3.064216	-4.037061	-1.048557
22	6	0	2.217991	-3.158732	1.403992
23	1	0	1.872830	-2.373255	2.078575
24	1	0	1.980964	-4.117792	1.868690
25	1	0	3.313566	-3.078455	1.324120
26	6	0	0.484863	-0.883315	0.179869
27	6	0	0.439926	0.543059	-0.384592
28	6	0	-0.782479	1.327107	0.032302
29	6	0	-1.640710	1.832554	-0.941499
30	6	0	-1.087473	1.528029	1.379296
31	6	0	-2.786417	2.530734	-0.577120
32	1	0	-1.416199	1.674930	-1.991683
33	6	0	-2.232504	2.222046	1.743345

34	1	0	-0.430445	1.140399	2.151283
35	6	0	-3.083561	2.726306	0.765418
36	1	0	-3.446833	2.919996	-1.344500
37	1	0	-2.461680	2.368669	2.793041
38	1	0	-3.976099	3.271759	1.052652
39	7	0	-0.850466	-1.529216	-0.069313
40	8	0	-1.537023	-1.764121	0.917135
41	8	0	-1.194047	-1.719607	-1.208620
42	1	0	0.580478	-0.866245	1.262690
43	1	0	0.482393	0.486512	-1.473781
44	7	0	1.540986	-3.075668	0.118683
45	6	0	-6.173643	-0.899457	-1.033430
46	6	0	-6.076637	0.417433	0.894839
47	6	0	-6.391212	1.328255	-0.294253
48	6	0	-5.855299	0.520392	-1.477122
49	1	0	-5.524353	-1.656773	-1.475299
50	1	0	-7.218508	-1.156807	-1.252223
51	1	0	-6.857569	0.435273	1.659855
52	1	0	-5.121648	0.679563	1.359956
53	1	0	-7.470320	1.476073	-0.394980
54	1	0	-5.924473	2.309841	-0.197263
55	1	0	-6.324558	0.780931	-2.427557
56	1	0	-4.773898	0.649149	-1.571003
57	8	0	-5.964219	-0.914463	0.377825
58	1	0	-3.754417	-1.486912	1.860697
59	1	0	-3.413642	-0.576798	0.030291
60	1	0	-3.819016	-2.607782	0.115831
61	5	0	-3.596948	-1.570129	0.674811

I1G

Zero-point correction= 0.392000 (Hartree/Particle)
Thermal correction to Energy= 0.415750
Thermal correction to Enthalpy= 0.416694
Thermal correction to Gibbs Free Energy= 0.336718
Sum of electronic and zero-point Energies= -1420.238394
Sum of electronic and thermal Energies= -1420.214644
Sum of electronic and thermal Enthalpies= -1420.213699
Sum of electronic and thermal Free Energies= -1420.293675

Center Number	Atomic Number	Atomic Type	Coordinates (Angstroms) X	Y	Z
1	6	0	-1.890579	-0.150511	-0.183946
2	6	0	-1.544724	1.106134	-0.473780
3	6	0	-0.153868	1.679901	-0.562976
4	1	0	0.107570	1.779005	-1.622459
5	1	0	-2.344450	1.810649	-0.685100
6	16	0	-0.722238	-1.457263	0.106372
7	6	0	-3.308243	-0.572009	-0.053157
8	6	0	-3.782403	-1.732439	-0.670762
9	6	0	-4.195006	0.208846	0.693254
10	6	0	-5.119627	-2.091582	-0.559091
11	1	0	-3.108207	-2.346640	-1.257964
12	6	0	-5.531408	-0.152520	0.803485
13	1	0	-3.828287	1.094883	1.200771
14	6	0	-5.997481	-1.303410	0.177091
15	1	0	-5.476703	-2.989210	-1.052166
16	1	0	-6.207680	0.461541	1.388324
17	1	0	-7.040387	-1.587690	0.266201
18	6	0	-0.426360	4.073790	-0.863215
19	1	0	-0.152062	5.035883	-0.426121
20	1	0	0.076751	3.987657	-1.828370
21	1	0	-1.515272	4.081420	-1.035427
22	6	0	-0.554494	3.154102	1.355334
23	1	0	-0.209801	2.357903	2.017396
24	1	0	-0.216699	4.101023	1.780042
25	1	0	-1.655385	3.147870	1.361860
26	6	0	0.883719	0.735748	0.047480
27	6	0	0.808037	-0.684085	-0.527866
28	6	0	1.998685	-1.539711	-0.165823
29	6	0	2.809571	-2.048306	-1.177857
30	6	0	2.328249	-1.793824	1.166825
31	6	0	3.936399	-2.800096	-0.864996
32	1	0	2.563438	-1.851412	-2.216480
33	6	0	3.453944	-2.543428	1.478446

34	1	0	1.705569	-1.406993	1.967345
35	6	0	4.260294	-3.047960	0.462990
36	1	0	4.561585	-3.188911	-1.661058
37	1	0	3.702986	-2.732638	2.516659
38	1	0	5.140715	-3.631713	0.708317
39	7	0	2.229462	1.314732	-0.266278
40	8	0	2.941679	1.565802	0.732069
41	8	0	2.573564	1.483517	-1.394816
42	1	0	0.841844	0.715667	1.133565
43	1	0	0.720816	-0.617732	-1.613638
44	7	0	0.011762	3.007008	0.022325
45	1	0	4.815130	2.259165	1.636974
46	1	0	4.999212	1.295977	-0.154968
47	1	0	4.281050	3.186494	-0.102493
48	5	0	4.427068	2.143091	0.499572

TS2G

Zero-point correction= 0.387315 (Hartree/Particle)
Thermal correction to Energy= 0.410466
Thermal correction to Enthalpy= 0.411411
Thermal correction to Gibbs Free Energy= 0.333839
Sum of electronic and zero-point Energies= -1420.180500
Sum of electronic and thermal Energies= -1420.157349
Sum of electronic and thermal Enthalpies= -1420.156405
Sum of electronic and thermal Free Energies= -1420.233976

Center Number	Atomic Number	Atomic Type	Coordinates (Angstroms) X	Y	Z
1	6	0	-1.829966	0.074552	-0.149436
2	6	0	-1.419581	1.332855	-0.338908
3	6	0	-0.023668	1.821078	-0.149713
4	1	0	0.145940	2.674511	-0.800403
5	1	0	-2.146616	2.075497	-0.651514
6	16	0	-0.707508	-1.217391	0.300768
7	6	0	-3.256277	-0.319755	-0.238620
8	6	0	-3.648141	-1.486430	-0.900701
9	6	0	-4.234932	0.491512	0.342748
10	6	0	-4.992573	-1.819240	-0.999931
11	1	0	-2.898750	-2.125762	-1.354107
12	6	0	-5.578530	0.153994	0.246113
13	1	0	-3.937761	1.381312	0.887686
14	6	0	-5.960785	-1.000984	-0.427383
15	1	0	-5.284547	-2.720998	-1.526998
16	1	0	-6.327198	0.789687	0.706224
17	1	0	-7.009896	-1.266310	-0.500610
18	7	0	0.346417	2.287211	1.272899
19	6	0	0.633664	3.722226	1.370880
20	1	0	1.068116	3.929354	2.348443
21	1	0	1.346063	4.003176	0.598155
22	1	0	-0.284415	4.308242	1.257249
23	6	0	-0.534222	1.828499	2.350462
24	1	0	-0.641483	0.745298	2.311733
25	1	0	-0.077339	2.102103	3.300998
26	1	0	-1.522616	2.290737	2.279905
27	6	0	1.113449	0.804199	-0.301350
28	6	0	0.848259	-0.672060	-0.515440
29	6	0	1.924902	-1.596825	0.018120
30	6	0	2.340389	-2.681886	-0.749652
31	6	0	2.474686	-1.411458	1.284929
32	6	0	3.300233	-3.562430	-0.265404
33	1	0	1.918784	-2.832591	-1.738771

34	6	0	3.436311	-2.288792	1.769693
35	1	0	2.155715	-0.575348	1.898829
36	6	0	3.852174	-3.366719	0.995403
37	1	0	3.620129	-4.399379	-0.876692
38	1	0	3.861599	-2.129585	2.754735
39	1	0	4.604542	-4.050574	1.373283
40	7	0	2.190682	1.262192	-1.096171
41	8	0	2.956722	0.506904	-1.638472
42	1	0	1.309962	1.376847	0.948389
43	1	0	0.711649	-0.863022	-1.584001
44	8	0	2.360956	2.535440	-1.131774
45	1	0	3.456221	4.318650	-1.717029
46	1	0	4.615843	2.690106	-1.340970
47	1	0	3.546629	2.792025	-3.057356
48	5	0	3.623217	3.129138	-1.891088

I2G

Zero-point correction= 0.394476 (Hartree/Particle)
Thermal correction to Energy= 0.417578
Thermal correction to Enthalpy= 0.418523
Thermal correction to Gibbs Free Energy= 0.341418
Sum of electronic and zero-point Energies= -1420.234525
Sum of electronic and thermal Energies= -1420.211422
Sum of electronic and thermal Enthalpies= -1420.210478
Sum of electronic and thermal Free Energies= -1420.287583

Center Number	Atomic Number	Atomic Type	Coordinates (Angstroms) X	Y	Z
1	6	0	-1.791132	-0.020390	-0.192418
2	6	0	-1.418938	1.224088	-0.512045
3	6	0	-0.028610	1.766343	-0.425805
4	1	0	0.108084	2.515176	-1.208252
5	1	0	-2.174788	1.911101	-0.877683
6	16	0	-0.623529	-1.255115	0.280834
7	6	0	-3.213061	-0.440885	-0.187093
8	6	0	-3.610364	-1.664536	-0.732680
9	6	0	-4.181078	0.402232	0.366081
10	6	0	-4.951634	-2.023486	-0.746414
11	1	0	-2.869490	-2.327872	-1.164680
12	6	0	-5.520999	0.038185	0.356308
13	1	0	-3.878480	1.339214	0.821657
14	6	0	-5.909620	-1.174571	-0.202278
15	1	0	-5.249095	-2.969941	-1.184382
16	1	0	-6.261289	0.699009	0.793847
17	1	0	-6.955858	-1.460309	-0.208413
18	7	0	0.142885	2.617890	0.855372
19	6	0	-0.726072	3.821196	0.853755
20	1	0	-0.432952	4.462653	1.681990
21	1	0	-0.594311	4.352847	-0.086571
22	1	0	-1.763432	3.515881	0.972819
23	6	0	0.005349	1.844350	2.112398
24	1	0	0.698349	1.008184	2.091470
25	1	0	0.237191	2.497955	2.950405
26	1	0	-1.016107	1.477659	2.192070
27	6	0	1.083778	0.770417	-0.540827
28	6	0	0.880134	-0.713904	-0.644519
29	6	0	1.986550	-1.592291	-0.091097
30	6	0	2.292637	-2.784746	-0.740571
31	6	0	2.656899	-1.272278	1.087919
32	6	0	3.258122	-3.642859	-0.225313
33	1	0	1.776474	-3.043696	-1.660307

34	6	0	3.623531	-2.124331	1.602177
35	1	0	2.440157	-0.339606	1.598595
36	6	0	3.926386	-3.314556	0.947519
37	1	0	3.489496	-4.566494	-0.744930
38	1	0	4.145948	-1.856589	2.514304
39	1	0	4.683053	-3.979924	1.349136
40	7	0	2.273391	1.265481	-0.853381
41	8	0	3.238839	0.622013	-1.258049
42	1	0	1.114183	2.937399	0.762587
43	1	0	0.700456	-0.991258	-1.688497
44	8	0	2.390948	2.595157	-0.654840
45	1	0	3.327820	4.504767	-1.004848
46	1	0	4.647103	2.995348	-0.738747
47	1	0	3.630141	3.155175	-2.485020
48	5	0	3.610061	3.347264	-1.278260

TS3G

Zero-point correction= 0.389613 (Hartree/Particle)
Thermal correction to Energy= 0.413501
Thermal correction to Enthalpy= 0.414445
Thermal correction to Gibbs Free Energy= 0.334936
Sum of electronic and zero-point Energies= -1420.217570
Sum of electronic and thermal Energies= -1420.193682
Sum of electronic and thermal Enthalpies= -1420.192738
Sum of electronic and thermal Free Energies= -1420.272247

Center Number	Atomic Number	Atomic Type	Coordinates (Angstroms) X	Y	Z
1	6	0	-1.768972	-0.084174	-0.463864
2	6	0	-1.369966	1.159434	-0.812913
3	6	0	-0.001131	1.582012	-0.810064
4	1	0	0.202267	2.594224	-1.123304
5	1	0	-2.118896	1.899262	-1.069157
6	16	0	-0.635663	-1.351240	-0.050830
7	6	0	-3.194109	-0.447407	-0.318383
8	6	0	-3.672799	-1.689485	-0.747571
9	6	0	-4.087673	0.464205	0.255265
10	6	0	-5.019466	-2.002927	-0.622287
11	1	0	-2.995173	-2.401784	-1.204368
12	6	0	-5.431462	0.144470	0.384248
13	1	0	-3.724365	1.418320	0.621256
14	6	0	-5.900883	-1.088897	-0.055946
15	1	0	-5.380797	-2.963958	-0.970864
16	1	0	-6.112058	0.856619	0.837391
17	1	0	-6.951362	-1.338216	0.046003
18	7	0	0.032930	2.594948	1.276117
19	6	0	-0.636420	3.881899	1.200345
20	1	0	-0.620205	4.424524	2.154501
21	1	0	-0.156613	4.500639	0.439994
22	1	0	-1.680400	3.727926	0.913720
23	6	0	-0.509033	1.705937	2.286428
24	1	0	0.060990	0.776509	2.304350
25	1	0	-0.496853	2.149677	3.290440
26	1	0	-1.544017	1.460268	2.033652
27	6	0	1.061351	0.696242	-0.820171
28	6	0	0.951427	-0.806419	-0.829676
29	6	0	2.021701	-1.558449	-0.060725
30	6	0	2.538565	-2.738520	-0.586533
31	6	0	2.463111	-1.117789	1.185848
32	6	0	3.490387	-3.466485	0.119126
33	1	0	2.202080	-3.088242	-1.557815

34	6	0	3.418734	-1.838441	1.887865
35	1	0	2.067894	-0.198720	1.607058
36	6	0	3.934044	-3.017036	1.356612
37	1	0	3.888430	-4.382895	-0.302908
38	1	0	3.762674	-1.479231	2.851667
39	1	0	4.680640	-3.580539	1.905588
40	7	0	2.317284	1.223633	-1.058973
41	8	0	3.245716	0.540558	-1.432148
42	1	0	1.030698	2.718817	1.394635
43	1	0	0.937886	-1.169192	-1.861432
44	8	0	2.451370	2.494210	-0.848284
45	1	0	3.566960	4.351419	-0.971712
46	1	0	4.691173	2.720877	-0.524182
47	1	0	4.021489	2.993448	-2.414426
48	5	0	3.817107	3.190569	-1.229797

I3G

Zero-point correction= 0.294030 (Hartree/Particle)
Thermal correction to Energy= 0.313143
Thermal correction to Enthalpy= 0.314087
Thermal correction to Gibbs Free Energy= 0.244184
Sum of electronic and zero-point Energies= -1285.151373
Sum of electronic and thermal Energies= -1285.132260
Sum of electronic and thermal Enthalpies= -1285.131316
Sum of electronic and thermal Free Energies= -1285.201219

Center Number	Atomic Number	Atomic Type	Coordinates (Angstroms) X	Y	Z
1	6	0	-1.831530	0.448275	-0.124781
2	6	0	-1.392787	1.734294	-0.109931
3	6	0	-0.015495	2.082758	-0.065711
4	1	0	0.234532	3.120651	0.115672
5	1	0	-2.118675	2.537749	-0.090527
6	16	0	-0.746725	-0.913787	-0.143468
7	6	0	-3.260895	0.097536	-0.014658
8	6	0	-3.806360	-0.949852	-0.764965
9	6	0	-4.090447	0.823718	0.847865
10	6	0	-5.157892	-1.250747	-0.666538
11	1	0	-3.179574	-1.510743	-1.448918
12	6	0	-5.438571	0.513777	0.947844
13	1	0	-3.671776	1.614516	1.460379
14	6	0	-5.975658	-0.521830	0.189395
15	1	0	-5.572714	-2.055356	-1.263151
16	1	0	-6.070153	1.077054	1.625577
17	1	0	-7.030200	-0.762029	0.268348
18	6	0	1.003340	1.212366	-0.269752
19	6	0	0.870608	-0.220725	-0.719957
20	6	0	1.916173	-1.173291	-0.171676
21	6	0	2.528433	-2.087288	-1.023350
22	6	0	2.247935	-1.167766	1.182640
23	6	0	3.470987	-2.982296	-0.530246
24	1	0	2.276445	-2.093968	-2.079260
25	6	0	3.195769	-2.055393	1.673185
26	1	0	1.766853	-0.466005	1.857483
27	6	0	3.808407	-2.965815	0.817696
28	1	0	3.945137	-3.688972	-1.202464
29	1	0	3.454993	-2.037647	2.726016
30	1	0	4.547667	-3.660042	1.202098
31	7	0	2.318731	1.751564	-0.322134
32	8	0	3.187925	1.192151	-0.934872
33	1	0	0.877370	-0.266939	-1.811911

34	8	0	2.509938	2.848540	0.293141
35	1	0	3.743512	4.575749	0.797976
36	1	0	4.753768	2.814990	0.626259
37	1	0	4.101996	3.761582	-1.037100
38	5	0	3.928462	3.569440	0.151087

TS4G

Zero-point correction= 0.412276 (Hartree/Particle)
Thermal correction to Energy= 0.437782
Thermal correction to Enthalpy= 0.438726
Thermal correction to Gibbs Free Energy= 0.352711
Sum of electronic and zero-point Energies= -1517.459271
Sum of electronic and thermal Energies= -1517.433766
Sum of electronic and thermal Enthalpies= -1517.432821
Sum of electronic and thermal Free Energies= -1517.518836

Center Number	Atomic Number	Atomic Type	Coordinates (Angstroms) X	Y	Z
1	6	0	-3.283441	0.482368	-0.117378
2	6	0	-2.817464	1.735029	-0.308341
3	1	0	-3.510958	2.566887	-0.276682
4	16	0	-2.236745	-0.926604	-0.133594
5	6	0	-4.692406	0.197533	0.228759
6	6	0	-5.371425	-0.885833	-0.338006
7	6	0	-5.367694	1.025747	1.131912
8	6	0	-6.702828	-1.121971	-0.022258
9	1	0	-4.864689	-1.530450	-1.047373
10	6	0	-6.696944	0.783482	1.447965
11	1	0	-4.840894	1.847661	1.604319
12	6	0	-7.368326	-0.289448	0.870420
13	1	0	-7.221692	-1.957987	-0.478033
14	1	0	-7.207132	1.428903	2.154426
15	1	0	-8.406848	-0.478436	1.119352
16	6	0	-0.503314	1.101539	-0.807994
17	6	0	-0.725907	-0.369986	-1.044329
18	6	0	0.412239	-1.234883	-0.532698
19	6	0	0.945295	-2.236182	-1.337989
20	6	0	0.941017	-1.027088	0.741099
21	6	0	2.000901	-3.017255	-0.880613
22	1	0	0.544390	-2.397753	-2.333751
23	6	0	2.001340	-1.799593	1.194220
24	1	0	0.529700	-0.251156	1.379555
25	6	0	2.533265	-2.797703	0.384297
26	1	0	2.412478	-3.792249	-1.517938
27	1	0	2.413431	-1.619451	2.181268
28	1	0	3.364295	-3.399260	0.736816
29	7	0	0.838363	1.565296	-1.103913
30	8	0	1.457889	0.980805	-1.963140
31	1	0	-0.881892	-0.564464	-2.107753
32	6	0	6.065011	0.690345	-0.390433
33	6	0	5.397229	0.632791	1.845547

34	6	0	5.692491	-0.802408	1.396505
35	6	0	5.544197	-0.713924	-0.123552
36	1	0	5.673188	1.141110	-1.303199
37	1	0	7.162566	0.703663	-0.431408
38	1	0	6.044584	0.958617	2.664570
39	1	0	4.354874	0.753272	2.155737
40	1	0	6.714694	-1.087398	1.661412
41	1	0	5.013277	-1.525126	1.851788
42	1	0	6.109172	-1.481117	-0.656426
43	1	0	4.493468	-0.789425	-0.413909
44	8	0	5.618249	1.478953	0.711502
45	1	0	3.239518	2.984711	0.973299
46	1	0	3.130503	1.102826	0.115952
47	1	0	3.797453	2.710459	-1.004543
48	5	0	3.319439	2.279150	0.006131
49	6	0	-1.426595	2.029164	-0.516462
50	1	0	-1.110538	3.064459	-0.460134
51	8	0	1.287535	2.514521	-0.455503
